# A Versatile Viral System for Expression and Depletion of Proteins in Mammalian Cells

**DOI:** 10.1371/journal.pone.0006529

**Published:** 2009-08-06

**Authors:** Eric Campeau, Victoria E. Ruhl, Francis Rodier, Corey L. Smith, Brittany L. Rahmberg, Jill O. Fuss, Judith Campisi, Paul Yaswen, Priscilla K. Cooper, Paul D. Kaufman

**Affiliations:** 1 Program in Gene Function and Expression, University of Massachusetts Medical School, Worcester, Massachusetts, United States of America; 2 Life Sciences Division, Lawrence Berkeley National Laboratory, Berkeley, California, United States of America; 3 Buck Institute for Age Research, Novato, California, United States of America; University of Toronto, Canada

## Abstract

The ability to express or deplete proteins in living cells is crucial for the study of biological processes. Viral vectors are often useful to deliver DNA constructs to cells that are difficult to transfect by other methods. Lentiviruses have the additional advantage of being able to integrate into the genomes of non-dividing mammalian cells. However, existing viral expression systems generally require different vector backbones for expression of cDNA, small hairpin RNA (shRNA) or microRNA (miRNA) and provide limited drug selection markers. Furthermore, viral backbones are often recombinogenic in bacteria, complicating the generation and maintenance of desired clones. Here, we describe a collection of 59 vectors that comprise an integrated system for constitutive or inducible expression of cDNAs, shRNAs or miRNAs, and use a wide variety of drug selection markers. These vectors are based on the Gateway technology (Invitrogen) whereby the cDNA, shRNA or miRNA of interest is cloned into an Entry vector and then recombined into a Destination vector that carries the chosen viral backbone and drug selection marker. This recombination reaction generates the desired product with >95% efficiency and greatly reduces the frequency of unwanted recombination in bacteria. We generated Destination vectors for the production of both retroviruses and lentiviruses. Further, we characterized each vector for its viral titer production as well as its efficiency in expressing or depleting proteins of interest. We also generated multiple types of vectors for the production of fusion proteins and confirmed expression of each. We demonstrated the utility of these vectors in a variety of functional studies. First, we show that the FKBP12 Destabilization Domain system can be used to either express or deplete the protein of interest in mitotically-arrested cells. Also, we generate primary fibroblasts that can be induced to senesce in the presence or absence of DNA damage. Finally, we determined that both isoforms of the AT-Rich Interacting Domain 4B (ARID4B) protein could induce G1 arrest when overexpressed. As new technologies emerge, the vectors in this collection can be easily modified and adapted without the need for extensive recloning.

## Introduction

The abilities to express and deplete proteins in mammalian cells are invaluable tools for understanding diverse biological processes, both normal and pathological. Traditionally, transfection and electroporation of plasmids into cells have been used to manipulate gene expression. However, not all cell types can be efficiently transfected by these methods, and primary or non-dividing cell types are particularly difficult in this regard [1]. The advent of recombinant retroviruses and lentiviruses [1], [2], [3], [4], [5] greatly simplified this task, especially since lentiviruses can easily transduce non-dividing cells. For RNA interference (RNAi), plasmid-based systems used to express shRNAs or miRNAs allowed long term depletion of the protein of interest, compared to the transfection of small interfering RNAs (siRNAs) which results in only transient depletion. Several laboratories and companies have therefore developed viral vectors for the delivery of cDNAs and/or shRNAs or miRNAs to a wide variety of cells [6], [7], [8], [9], [10], [11], [12], [13], [14], [15], [16]. Even though these vectors have proven extremely useful, there are limited drug selection markers available, and different vector backbones can be required to express cDNAs or shRNAs/miRNAs. We sought to extend the versatility of viral vectors by developing third generation (Tat-independent), self-inactivating (SIN) lentiviral vectors with an expanded range of drug selection markers, promoters, and epitope tags. Furthermore, we wanted to improve their flexibility, allowing the investigator to rapidly change promoters and/or drug selection markers without extensive recloning. We report here a series of 59 vectors to express cDNAs, shRNAs and miRNAs, either constitutively or inducibly, in mammalian cells. These vectors are based on the Gateway System [17] (Invitrogen) whereby a cDNA, shRNA or miRNA is cloned into an Entry vector which is recombined *in vitro* with the viral Destination vector of choice. This system offers two main advantages: First, the recombination is very efficient and rapid (1–3 h), eliminating most of the stability problems that often arise during traditional cloning in a bacterial host [18], [19]. Second, the extensive Destination vector library can be further modified to accommodate future technologies without the need for recloning cDNAs, shRNAs or miRNAs. We used our vectors to demonstrate that a protein of interest can be overexpressed or depleted in mitotically-arrested cells, generate stable cell lines where the senescence secretory associated phenotype (SASP) can be induced, and show that the chromo domain of the AT-Rich Interacting Domain 4B (ARID4B) protein is not required for the G1 arrest upon overexpression.

## Results and Discussion

### Overview of the vectors

**Figure 1:**
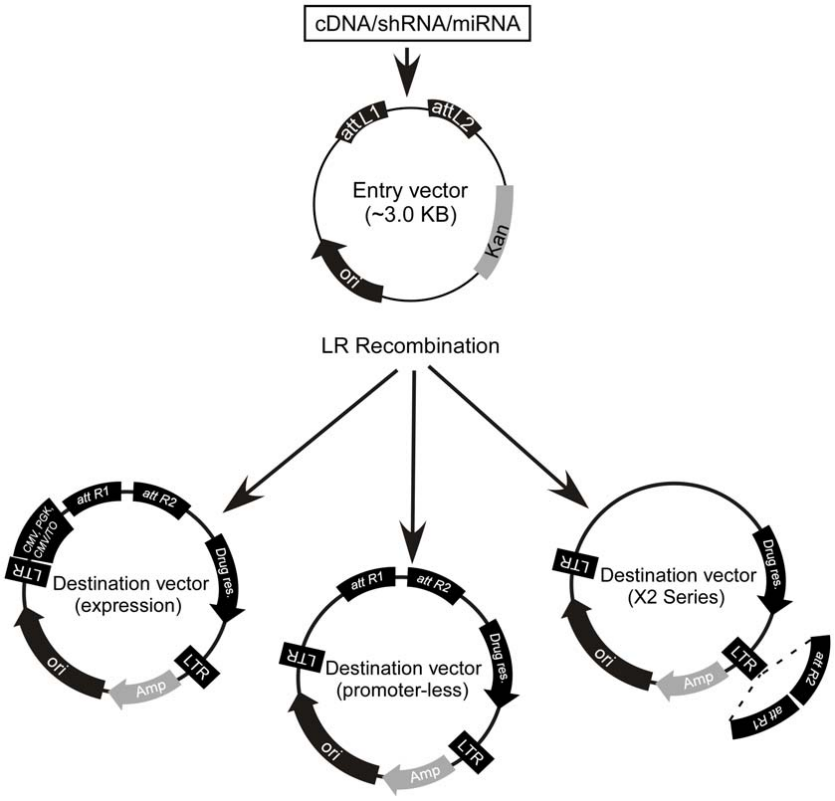
Overview of the viral system. A cDNA/shRNA/miRNA is cloned into an Entry vector between the attL1 and attL2 sites. In the presence of the LR clonase, recombination occurs between attL1-attR1 and attL2-attR2 to transfer the insert from the Entry vector into the Destination vector of choice. All Entry vectors contain the kanamycin resistance gene whereas all Destination vectors carry the ampicillin resistance gene.

cDNAs, shRNAs or miRNAs are initially cloned into Entry vectors (Figure 1). The insert from the Entry vector clone can then be recombined efficiently via an attL-attR (LR) reaction [17] into a variety of Destination vectors to provide the desired viral backbone and drug selection marker. The recombination reaction is transformed into *Escherichia coli* and there is a dual selection for the desired vector. First, the Entry and Destination vectors carry different drug resistance genes for selection in *E.coli*, kanamycin for the Entry vector, and ampicillin for the Destination vector. The transformation is plated onto ampicillin plates, selecting against any unrecombined Entry vector. Second, the Destination vector contain the ccdB killer gene [20], which is toxic to most *E.coli* strains. The ccdB gene is removed from the Destination vector during the recombination reaction and a susceptible *E.coli* strain is used for the transformation, thereby selecting against the unrecombined Destination vector. In our hands, the efficiency of recombination into the Destination vector is >95% as determined by restriction digestion and analysis of the resulting construct. Because the Entry vectors do not contain any viral-derived repetitive sequences, they can be easily manipulated without significant levels of unwanted deletions which often occur at repetitive sequences propagated in bacteria [18], [19], [21].

### Entry vectors

#### i. for expression of shRNAs or miRNAs

**Figure 2:**
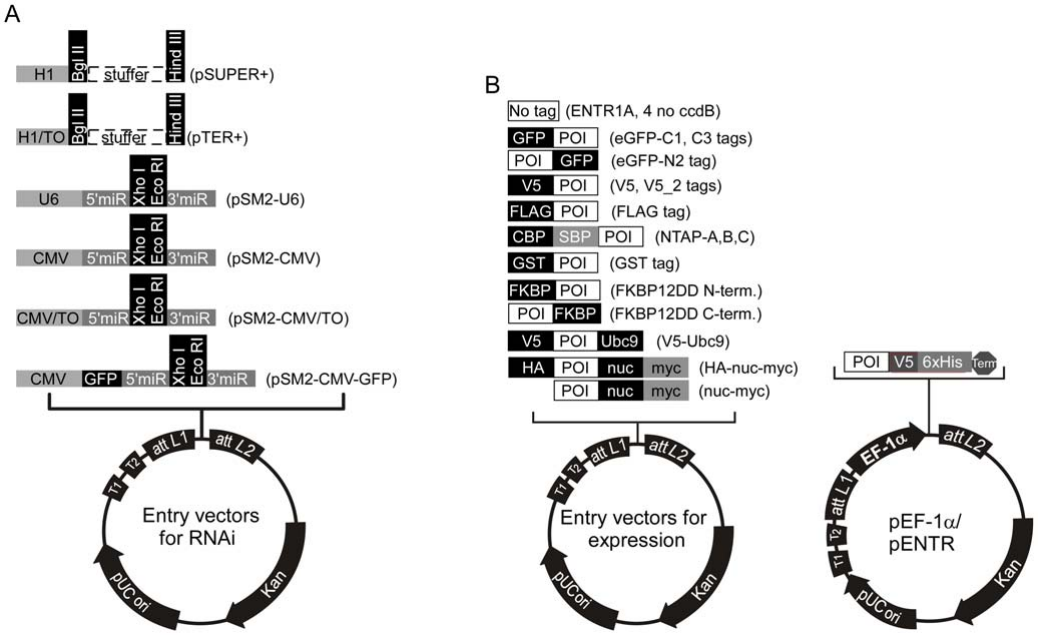
Maps of the Entry vectors for either RNAi-mediated protein depletion or protein overexpression. A) shRNAs or miRNAs can be expressed with either constitutive (H1, U6, CMV) or inducible (H1/TO, CMV/TO) promoters. B) cDNAs encoding the protein of interest (POI) can be cloned into Entry vectors with no tags or different tags for either detection or purification. (Abbreviations: CBP, calcium-binding peptide; SBP, S-binding peptide; GST, glutathione-S-transferase; FKBP12DD, FKBP destabilizing domain. LEFT: Promoter-less Entry vectors. RIGHT: Entry vectors for EF-1α promoter-driven protein expression. In this case, Entry vectors containing a promoter should be recombined with a promoter-less Destination vector (see Figure 4).

Six different Entry vectors were generated for RNAi experiments, and are suitable for insertion of either shRNAs or miRNAs (Figure 2A):

1. The pENTR/pSUPER+ plasmid is based on the pSUPER plasmid [22], which allows the expression of shRNAs under the control of the H1 RNA polymerase III promoter. We inserted a 750 bp stuffer (denoted by the +, derived from the pTER+ plasmid [23]) between the *Bgl* II and *Hin*d III sites to facilitate cloning of shRNAs downstream of the promoter.
2. The pENTR/pTER+ plasmid is derived from the pTER+ plasmid [23]; this is similar to pSUPER except that a Tetracycline Operator (TO) was inserted at the end of the H1 promoter. The TO allows experimental control of the expression of the shRNA in a “T-REx” cell line (Invitrogen) that expresses the tetracycline repressor (TetR), because the transcription-blocking TetR-TO interaction is relieved by addition of doxycycline or tetracycline [24].
3. The pENTR/pSM2 plasmids are based on the pSM2c plasmid [14], [25] for expression of miRNAs, and include the 5′ and 3′ flanking sequences from the miR-30 miRNA. The original U6 promoter was retained to allow constitutive expression under the control of RNA polymerase III in the pENTR/pSM2(U6) version of the vector. In the pENTR/pSM2(CMV) and pENTR/pSM2(CMV/TO) versions, the U6 promoter was replaced by either a constitutive CMV or a doxycycline-inducible CMV/TO promoter to allow expression under the control of RNA polymerase II. The CMV promoter was reported to generate better depletions in some cases [26] and lower variability than the U6-based promoter when the virus is integrated in the genome [27].
4. Finally, we generated the vector pENTR/pSM2(CMV-GFP), in which a GFP cDNA was inserted between the promoter and the 5′miR30 sequence, to further enhance the depletion of the target protein [8] and facilitate tracking and sorting of cells expressing the miRNA of interest.

#### ii. for expression of cDNAs

A large variety of Entry vectors were generated to clone cDNAs encoding proteins of interest (POI) (Figure 2B). The original pENTR1A and pENTR4 plasmids contain the ccdB killer gene. To clone a cDNA into these vectors, two restriction endonucleases which cut on opposite sides of the killer gene have to be used in order to replace the ccdB sequence with the cDNA of interest and thereby select against the parental plasmid. However, we removed the ccdB gene so that all of the multiple cloning sites could be exploited, generating constructs termed “pENTR1A no ccdB” and “pENTR4 no ccdB”. We also generated a variety of Entry vectors that encode epitope tags at their N-terminus (GFP, V5, FLAG) or C-terminus (GFP, V5-His) in different reading frames to facilitate detection of the proteins of interest. For tandem affinity purification (TAP) experiments, we created Entry vectors encoding tandem N-terminal epitope tags in three reading frames, based on the pNTAP vector from Stratagene (NTAP,[28]). Additionally, we created an Entry vector encoding an N-terminal Glutathione-S-Transferase (GST) followed by a PreScission protease cleavage site (GE Healthcare Life Sciences), which can also be used for purification of the protein of interest via glutathione affinity supports. Other Entry vectors include either an N-terminal or C-terminal 12 kDa FK506 Binding Protein (FKBP12) Destabilization Domain (DD) based on the Wandless lab design [29], [30] (also commercialized by Clontech under the name ProteoTuner). This domain targets the protein of interest to the proteosome for rapid degradation upon removal of the protective ligand Shield-1 (Shld1), with faster kinetics than RNAi-mediated depletion [30]. For the pENTR-V5-Ubc9 plasmid, the SUMO E2 ligase Ubc9 was inserted at the C-terminus of the pENTR-V5 vector so that cDNAs could be inserted between the V5 epitope and Ubc9, generating a fusion protein that facilitates detection of the sumoylated target protein, thereby bypassing the requirement for an E3 SUMO ligase [31]. Finally, we made two vectors to target protein domains to the nucleus, the pENTR-HA-nuc-myc and pENTR-nuc-myc, which are derived from the pCMV/myc/nuc (Invitrogen). These vectors have three tandem copies of the SV40 Large T antigen nuclear targeting sequence [32].

For cases where the CMV promoter might not be optimal for cDNA expression, we also developed Entry vectors that contain the human EF-1α promoter (Figure 2B). These vectors encode a C-terminal V5-His epitope tag and were derived from the pEF-TRACER A, B, C plasmids (Invitrogen). We also expanded the variety of promoters available by creating Destination vectors containing the human phosphoglycerate kinase (PGK) promoter (see below).

### Lentiviral Destination vectors

**Figure 3:**
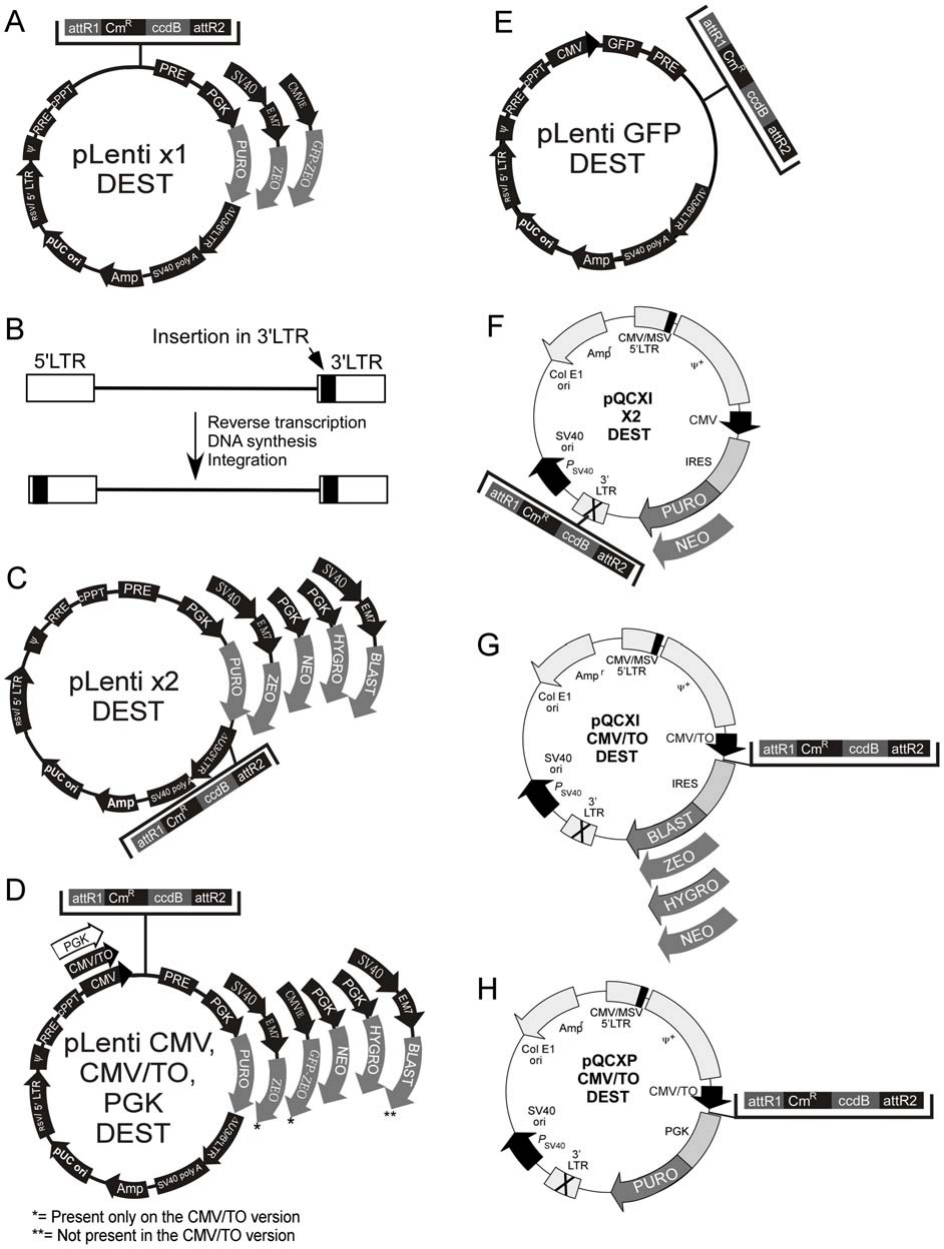
Maps of the Destination vectors. (A, C) The pLenti X1 and X2 series are promoter-less and require that the expression promoter come from the Entry vector. (B) The pLenti X2 series is designed for shRNA insertion into the 3′ LTR, resulting in insert duplication in the final integrated form of the viral genome. (D) The CMV and PGK series provide the promoters for constitutive expression, and the CMV/TO promoter for regulation of expression by doxycycline. (E) The pLenti GFP DEST allows insertion of an expression cassette after the Woodchuck post-transcriptional element (PRE) and expresses GFP to make recipient cells fluorescent. (F) Retroviral Destination vectors for RNAi where the Destination cassette was inserted in the 3′ LTR, similar to the pLenti X2 series. (G, H). Retroviral Destination vectors for cDNA expression under the control of the CMV promoter. See text, Table 1 and Figure 4 for details and compatibilities between each vector.

**Figure 4:**
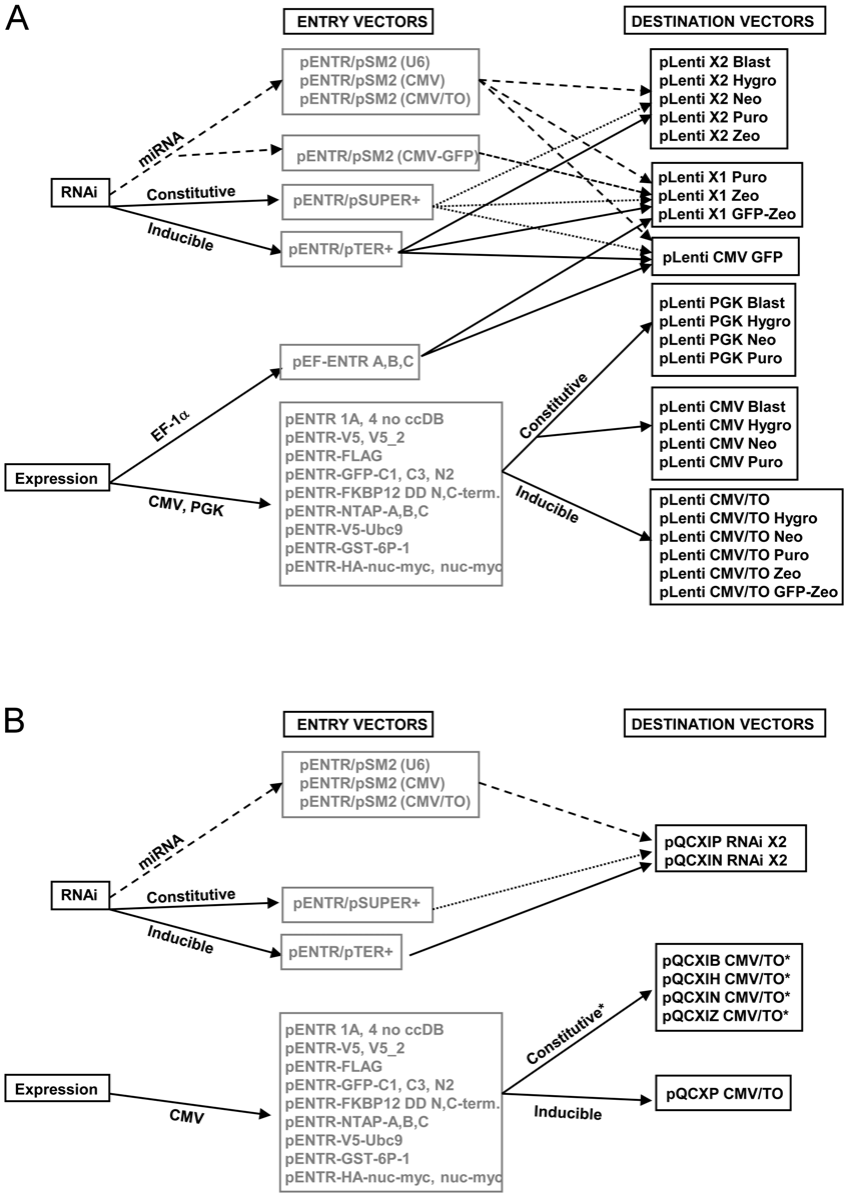
Overview of the lentiviral (A) or retroviral (B) vectors for protein expression or depletion. Entry vectors can be recombined using the LR clonase into the Destination vectors connected by arrows. For RNAi-mediated depletion, miRNA-based and shRNA-based vectors are available for either constitutive or inducible depletions (see Figure 2A for details). For expression, various tags are available (see Figure 2B for details). The RNAi cassettes can be inserted in the middle of the lentiviral backbone (pLenti X1 series), in the 3′ LTR (pLenti X2 series) or in a backbone expressing GFP (pLenti CMV GFP). For RNAi studies using retroviruses, all the Destination cassettes have been inserted in the 3′ LTR of the vectors. For protein expression, the EF-1α, PGK or CMV promoters are available for lentiviruses. CMV-driven constructs can be either constitutive or inducible. For the retroviral vectors, no constitutive expression of cDNAs can be attained in T-REx cell lines using the pQCXI series vectors because they all contain the inducible CMV/TO promoter. Similarly, the drug resistance gene will be repressed in a T-REx cell line since it is after an IRES element under the control of the CMV/TO promoter. However, the pQCXP CMV/TO can be used for inducible expression of cDNAs under constitutive puromycin selection in T-REx cell lines. See Table 2 for details.

Using the Gateway technology, each Entry vector can be recombined into multiple Destination vectors. Every lentiviral Destination vector contains the Woodchuck post-transcriptional regulatory element (WPRE) and the central polypurine tract (cPPT) because these elements increase transduction efficiencies [33], [34], [35], [36]. The maps of the Destination vectors are shown in Figure 3. However, not all combinations of Entry and Destination vectors are meant to be recombined. For example, if an Entry vector already contains a promoter (such as the vectors used for RNAi (Figure 2A) or the pEF-ENTR series (Figure 2B)), recombination with a Destination vector such as the pLenti CMV, CMV/TO or PGK series would juxtapose the two promoters and possibly cause interference. Also, recombination of a promoter-less Entry vector for expression with a promoter-less Destination vector would result in a construct with no promoter to drive expression of the cDNA. Some Destination vectors insert the Entry cassette in their 3′LTR (see below), but this imposes an insert size limit, usually ∼800 bp [37], because larger inserts inactivate the LTR and impair viral integration. Also, some sequences can be detrimental to the function of the 3′LTR [38]. Finally, the pENTR/pSM2 (CMV-GFP) vector encodes GFP and therefore should not be recombined with the pLenti CMV GFP or pLenti X1 GFP-Zeo Destination vectors since they also encode the GFP protein. The compatibilities of each Entry vector for various Destination vectors are summarized in Table 1 as well as represented schematically in Figures 4A for lentiviruses, and Figure 4B for retroviruses.

**Table 1:** Compatibles Entry to Destination vector recombinations.

Entry vector type	Compatible Destination vectors	Incompatible Destination vectors
pENTR/pSM2 Series (non-GFP)	pLenti X1 Series	pLenti PGK Series^b^
	pLenti X2 Series	pLenti CMV Series^b^
	pLenti CMV GFP	pLenti CMV/TO Series^b^
	pQCXI RNAi X2 Series^a^	pQCXI CMV/TO Series^b^
pENTR/pSM2(CMV-GFP)	pLenti X1 Series	pLenti X2 Series^c^
		pLenti CMV GFP^d^
		pQCXI CMV/TO Series^b^
		pQCXI RNAi X2 Series^c^
pENTR/pTER+ pENTR/pSUPER+	pLenti X1 Series	pLenti PGK Series^b^
	pLenti X2 Series	pLenti CMV Series^b^
	pLenti CMV GFP	pLenti CMV/TO Series^b^
	pQCXI RNAi X2 Series	pQCXI CMV/TO Series^b^
pEF-ENTR Series	pLenti X1 Series	pLenti X2 Series^c^
	pLenti CMV GFP	pLenti PGK Series^b^
		pLenti CMV Series^b^
		pLenti CMV/TO Series^b^
		pQCXI CMV/TO Series^b^
		pQCXI RNAi X2 Series^c^
pENTR-Fusion Series	pLenti PGK Series	pLenti X1 Series^e^
	pLenti CMV Series	pLenti X2 Seriesc,e
	pLenti CMV/TO Series	pLenti CMV GFP^e^
	pQCXI CMV/TO Series	pQCXI RNAi X2 Seriesc,e

a Viral titers will be lower by ∼100 fold.

b Probable promoter interference.

c Insert would be too big for the 3′LTR.

d Entry vector contains already a GFP.

e No promoter to drive expression of the cDNA.

#### i. pLenti X1 Destination series

The Gateway Destination cassette was inserted into a promoter-less lentiviral backbone (Figure 3A) because some of the Entry vectors provide the promoter for the expression of shRNA, miRNA or cDNA. One version of the vector confers resistance to puromycin and two others confer resistance to zeocin. Additionally, the pLenti X1 GFP-Zeo vector encodes a GFP-zeocin fusion protein to allow detection of transduced cells by fluorescence.

#### ii. pLenti X2 Destination series

Lentiviruses are part of the retrovirus family and have an RNA genome that is reverse-transcribed into DNA before integration into the host genome. During this process, there is a duplication of the 3′ LTR to generate the final 5′ LTR sequence that is found in the integrated viral genome. Deletions in the U3 region of the 3′ LTR (ΔU3) have been used to generate self-inactivating (SIN) vectors for increased biosafety [39], [40], and several groups have found that these truncated 3′ LTRs tolerate insertion of a small cassette, such as a small promoter (U6, H1, CMV) and an shRNA or miRNA [41], [42], [43]. Therefore, inclusion of a shRNA in the 3′ LTR of the original construct results in duplication of this transgene upon integration (Figure 3B). We therefore inserted the Gateway Destination cassette within the 3′ LTR (Figure 3C). All the vectors of the pLenti X2 series have the Gateway cassette inserted in the antisense orientation.

#### iii. pLenti CMV, CMV/TO and PGK Destination series

We generated two types of Destination vectors for expression of cDNAs under the control of the CMV promoter. The first type has a constitutive CMV promoter (Figure 3D, pLenti CMV), and the second has an inducible CMV/TO promoter (pLenti CMV/TO). The pLenti CMV/TO series is induced upon addition of doxycycline in a T-REx cell line that expresses the TetR tetracycline repressor protein. To allow generation of a wide variety of TetR-expressing cell lines, we also generated an Entry vector with the TetR cDNA (pENTR/TetR) and recombined it with the pLenti CMV Blast DEST vector to make pLenti TetR Blast (not shown). We transduced different cell lines with virus made from this vector, generating cells that express TetR for inducible transgene expression from TO-containing promoters. Because the TetR-expressing virus carries a blasticidin resistance gene, we did not generate an inducible CMV/TO lentiviral vector carrying the same drug resistance gene.

Loss of protein expression *in vivo* due to promoter silencing can occur with the CMV promoter, but silencing is much less frequent for transgenes driven by the PGK and EF-1α promoters [44], [45], [46]. Therefore, we generated Destination vectors with the PGK promoter (Figures 3D, 4A). The cellular EF-1αpromoter can also be used for this purpose, but the PGK promoter in the pLenti PGK series is smaller and therefore minimizes loss of viral titers when large cDNAs are inserted [47]. Also, the PGK Destination vectors are more versatile because they harbor more drug resistance genes, while the pEF-1α Entry vectors can only be recombined into the pLenti X1 Destination series (Figure 3A), which currently have only puromycin and zeocin resistance genes for selection.

#### iv) pLenti GFP DEST

We sought to create a vector that would allow detection of transduced cells via GFP expression while simultaneously delivering a transgene of choice. To do this, we created a vector in which GFP expression is under the control of the CMV promoter and either a cDNA, shRNA or miRNA can be placed under the control of the EF-1α, H1, H1/TO, U6, CMV or CMV/TO promoters (Figure 4A). GFP expression allows detection and/or isolation of transduced cells by either immunofluorescence or cell sorting. For this vector, we also inserted the Gateway Destination cassette after the WPRE (Figure 3E).

### Retroviral Destination vectors

In addition to the lentiviral vectors, we also generated retroviral Destination vectors (Figures 3F, G, H, and Figure 4B) to determine whether similar levels of expression or depletion could be attained. With the exception of the pEF-ENTR series and the pENTR/pSM2(CMV-GFP) vectors, all the other Entry vectors are compatible with these retroviral vectors, so we can easily recombine inserts into either retroviral and lentiviral vectors. Using the same strategy as the pLenti X2 series, we inserted the Gateway Destination cassette into the 3′ LTR of the retroviral backbone so we could obtain the expression of two shRNA/miRNA cassettes upon viral integration into the cellular genome (pQCXI X2 series, Figure 3F, 4B). The insertion of an shRNA cassette in the 3′ LTR of the pQCXIP plasmid was previously used successfully [48]. We generated two retroviral vectors with the neomycin and puromycin resistance genes for RNAi. For expression of cDNAs, we replaced the CMV promoter from the original pQCXI plasmid with the CMV/TO promoter with the Gateway Destination cassette (Figure 3G). We also inserted the zeocin and blasticidin resistance genes after the internal ribosome entry site (IRES) so we could use these drugs for selection. One possible drawback of these constructs is the control of the drug resistance gene by the CMV/TO promoter. In a T-REx cell line, both the cDNA and the drug resistance gene will be repressed, allowing selection of transduced cells only when the cDNA is induced. However, these vectors can be used to transduce non-T-REx cells for constitutive expression. A construct in which only the transgene and not the drug resistance gene is inducible was designed by replacing the IRES with a murine PGK promoter to control the puromycin resistance gene from an independent and non-inducible promoter (Figure 3H). Table 2 summarizes which vectors can be used to generate inducible expression of the cDNA/miRNA/shRNA in a T-Rex cell line. The vectors that cannot generate inducible expression will have constitutive expression in these cells.

**Table 2:** Capacity of vectors for inducible expression in a T-Rex cell line.

Vector	Inducible expression in a T-REx cell line
pENTR/pSUPER+	NO
pENTR/pTER +	YES
pENTR/pSM2(U6)	NO
pENTR/pSM2 (CMV)	NO
pENTR/pSM2 (CMV/TO)	YES
pLenti CMV Series	NO
pLenti CMV/TO Series	YES
pLenti PGK Series	NO
pQCXI CMV/TO Series	YES*
pQCXP	YES

* Both the cDNA and the drug resistance gene will be under the control of the CMV/TO promoter, except for pQCXP.

### Our vectors do not encode the HIV Tat protein

With each new generation of lentiviral packaging vectors, fewer HIV proteins are co-expressed with the recombinant viral genome, resulting in greater biological safety. In second generation vectors, most of the HIV proteins not required for viral replication are removed, with the exception of the HIV Tat protein which is required for transcription of the viral genome from the 5′ LTR. It is provided by the packaging plasmid [49], [50], [51] and the Tat protein is produced and secreted into the media by the packaging cell line. Transfection of a Tat-expressing plasmid results in the secretion of biologically active Tat [52], [53]. In third generation vectors, the 5′ HIV LTR was replaced by either a hybrid 5′ CMV/LTR or 5′ RSV/LTR to allow transcription of the viral genome in the absence of the HIV Tat protein without a decrease in viral titers [40]. Accordingly, third generation packaging plasmids do not express the HIV Tat protein. Because several studies have pointed out the toxicity of the HIV Tat protein and that it interferes with several cellular processes [54], [55], [56], [57], [58], [59], [60], [61], [62], [63], we used third generation vectors as the basis for our constructs and all of our lentiviral vectors have the hybrid 5′ RSV/HIV LTR and do not require the HIV Tat for transcription of the viral genome. We still recommend transduction with vectors encoding a control shRNA/cDNA or an empty vector at similar titers to monitor possible toxic effects of the transduction.

### Testing of the vectors

**Figure 5:**
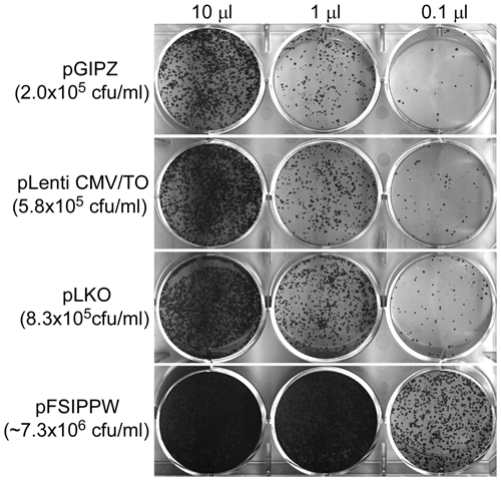
Comparison of titer efficiencies of various lentiviral vectors available to the scientific community. Twenty-five thousand HeLa cells were seeded in each well of a six-well plate and transduced with either 10, 1 or 0.1 µl of viral supernatants. Forty-eight hours post-transduction, puromycin was added at a final concentration of 0.5 µg/ml. Twelve days post-transduction, the cells were fixed and stained with crystal violet. The LKO.1, GIPZ and our viral vector (pLenti CMV/TO) yielded similar titers (2–8×10^5^ cfu/ml) whereas the FSIPPW yielded a higher titer (∼7×10^6^ cfu/ml).

We compared one of our vectors with other lentiviral vectors available to the scientific community to ensure that the insertion of the Gateway Destination and the various drug selection cassettes did not affect the efficiency of the virus. The pGIPZ (Open Biosystems), pLKO.1 (The RNAi Consortium) and the pFSIPPW [64] vectors were chosen because of their popularity and because they all use puromycin as a selection marker, thereby alleviating differences due to different drug selections. The empty versions of each vector were compared to the empty version of the pLenti CMV/TO Puro plasmid. Each virus was produced at the same time, using the same transfection protocol adjusting for second or third generation lentiviruses. As shown in Figure 5, the pLenti CMV/TO Puro, pGIPZ and pLKO vectors produced viruses with similar titers (2–8×10^5^ cfu/ml), indicating that our vectors have packaging efficiencies similar to several commercially available vectors. In contrast, the pFSIPPW vector produced higher viral titers (7×10^6^ cfu/ml). Both pFISPPW and pGIPZ are second generation vectors whereas pLKO and pLenti CMV/TO Puro are third generation, so this aspect does not explain the differences in the observed titers.

Each Destination vector was also tested to ensure it was functional and generated similar viral titers to other vectors (Table 3 and data not shown). However, we did not test every possible combination of Entry vector and Destination vectors due to the great number of such combinations (326 for the lentiviral vectors, 105 for the retroviral vectors). It is therefore possible that unforeseen Entry-Destination combinations might negatively affect the viral titers. We are maintaining a web site with up to date information of current as well as future vectors at http://ericcampeau.com.

**Table 3:** Viral titers for the different Entry to Destination vectors tested for RNAi.

Vector Series	pSUPER, pTER	pSM2-CMV, CMV/TO	pSM2 CMV-GFP	pEF-Series
Lenti X2 Blast	10^4^		N/A	N/A
Lenti X2 Hygro	10^5^	10^4^	N/A	N/A
Lenti X2 Neo	10^5^	10^5^	N/A	N/A
Lenti X2 Puro	10^5^		N/A	N/A
Lenti X2 Zeo	10^5^		N/A	N/A
Lenti X1 Puro	10^5^	10^5^	10^4^	
Lenti X1 Zeo	10^5^			
Lenti X1 GFP-Zeo	10^5^		N/A	
QCXIN X2	10^5^	10^3^	N/A	N/A
QCXIP X2	10^5^		N/A	N/A

N/A: Not applicable.

We titered nearly 250 different recombinant viruses, using either HT1080 or HeLa cells. For expression vectors, we normally obtain a titer of 10^5^ cfu/ml, regardless of the promoter or the drug resistance used (result not shown). Because viral titers generally decrease with increasing size of the inserted cDNA [47], both the size of the cDNA and the size of the drug resistance gene need to be accounted for when choosing a backbone. Some drug resistance cDNAs, e.g. for blasticidin and zeocin, are ∼400 bp whereas the hygromycin-resistance cDNA is ∼1000 bp. When expressing a large cDNA (>2.5 kb), the choice of the drug resistance backbone might influence the viral titer. In our hands, we did not notice any significant decrease in viral titers with cDNAs of less than 2.0 kb with any Destination vector. We have successfully expressed cDNAs ∼4 kb, with a reduced titer of 10^4^ cfu/ml. If needed, we would obtain higher viral titers by concentrating the virus by ultracentrifugation (see methods).

For the RNAi vectors, the results are summarized in Table 3. We typically obtain viral titers similar to the expression vectors (10^5^ cfu/ml). The notable exception was the retroviral pQCXIN X2 vector with a CMV-driven miRNA inserted in the 3′LTR, which yielded titers of 10^3^ cfu/ml. In contrast to vectors with a CMV promoter, the pQCXIN X2 and pQCXIP X2 vectors produced viral titers of 10^5^ cfu/ml when an H1 or H1/TO-driven shRNA was inserted in their 3′LTR. We observed that lentiviral vectors with either CMV-driven miRNAs or H1-driven shRNAs in their 3′LTR produced viruses with the expected titer of 10^5^ cfu/ml. Thus, it appears that lentiviruses are more tolerant than these retroviruses regarding the type or size of sequence that can be inserted at their 3′LTR. We therefore recommend the use of lentiviral rather than retroviral vectors when inserting a CMV-driven miRNA cassette in the 3′LTR. The various combinations of Entry to Destination vectors tested are indicated in Table 3. We will update our web site (http://ericcampeau.com) as new combinations will be generated through the course of our studies.

**Figure 6:**
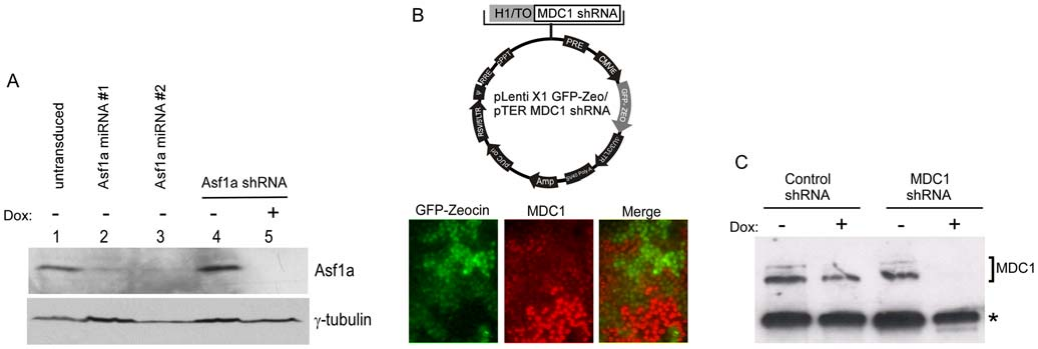
Depletion of Asf1a and MDC1 using various lentiviral vectors. A) Asf1a depletion in U2OS cells. Lane 1: U2OS T-REx cell extracts, untransduced. Lane 2 and 3: U2OS transduced with two different miRNAs against Asf1a in the pLenti X2 Hygro/pSM2(CMV) vector; Lane 4 and 5: U2OS T-REx cells transduced with the pLenti X2 Neo/pTER shAsf1a #1 plasmid either uninduced (lane 4) or induced for 96 h (lane 5). γ-tubulin levels were monitored as a loading control for protein levels. (B) Depletion of MDC1 using the pLenti X1 GFP-Zeo vector. TOP: Map of recombinant lentivirus used to deplete MDC1 with the GFP-Zeocin fusion protein. BOTTOM: Immunofluorescent detection of MDC1 knockdown. Transduced cells produce the GFP-Zeocin resistance fusion protein, and therefore can be detected by green fluorescence (left panel). MDC1 protein was detected using an MDC1 antibody and an Alexa568-labeled secondary antibody, shown in the red channel (middle panel). Merging of these two images (right panel) indicates that the cells expressing the GFP fusion protein are depleted of MDC1. (C) Inducible depletion of MDC1 in BJ T-REx fibroblasts using the pLenti X1 Zeo vector. The cells were induced for 96 h and collected for Western blot analysis. The same MDC1 antibody used in (B) was used for the detection of MDC1. A cross-reacting band (indicated by the asterisk, *) is used as a loading control. It is not depleted by the MDC1 shRNA and therefore, appears to be an endogenous cellular protein that is recognized fortuitously on immunoblots.

Each vector described in Figure 4 and Table 1 was also tested for functionality of the fusion protein (V5, FLAG, GFP, NTAP, FKBP12DD, V5-Ubc9, GST, myc and nuclear targeting), confirming epitope detection by immunoblotting or immunofluorescence (Figures 6–8 and Supplemental Figures S1 and S2). We also confirmed the ability of each promoter to drive expression of downstream cDNA, shRNA, miRNA or drug selection genes. Unless mentioned, we analyzed the entire cell population that survived the drug selection, as opposed to a population derived from a single cell clone. Because viral vectors can integrate into many areas of the genome, single cell-derived (clonal) populations may provide better inducibility/repression of the desired cDNA/shRNA/miRNA.

**Figure 7 pone-0006529-g007:**
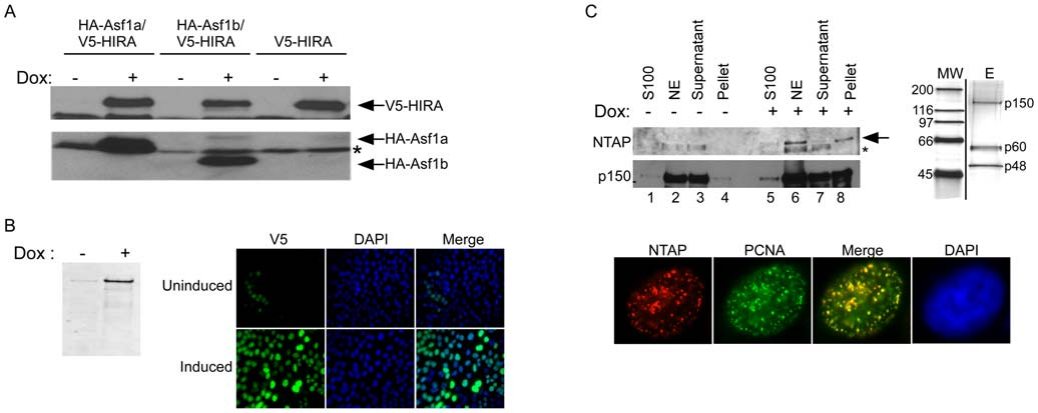
Use of the lentiviral Destination vectors to overexpress proteins. (A) Generation of three VA13 cell lines inducibly expressing either V5-HIRA, V5-HIRA and HA-Asf1a, or V5-HIRA and HA-Asf1b. (A) Cells were first transduced with the pLenti TetR Blast lentivirus and selected with blasticidin. These cells where then transduced with either the pLenti CMV/TO V5-HIRA Hygro virus alone or in combination with either the pLenti CMV/TO HA-Asf1a Puro or pLenti CMV/TO HA-Asf1b Puro virus. After appropriate drug selection, the cells were tested for the expression of the respective proteins before or after induction with Doxycycline for 48 h by anti-epitope Western blot. The * represents a cross-reacting band detected by the anti-HA antibody used as loading control. (B) Inducible expression of XPG-V5 from pLenti CMV/TO Zeo in U2OS cells monitored by Western blot (left) and immunofluorescence (right). In the whole population, very few cells (<1%) showed leakiness in the uninduced control; we photographed a field with some leaky uninduced cells. (C) LEFT: Western blot of Streptavidin pulldowns from HeLa S3 TREx-NTAP-p150 nuclear extracts (NE) without (lanes 1–4) or with (lanes 5–8) doxycycline induction (2 µg/ml) probed with anti-CBP (NTAP blot) or anti-p150. The distribution of NTAP-p150 during the nuclear extraction is the same as the endogenous p150, mainly absent from the cytosolic (S100) extract and present in the nuclear extract. The arrow represents NTAP-p150, where as the asterisk represents cross-reactivity of the antibody since it is in the uninduced samples and is not pelleted by the streptavidin beads (lane 8). RIGHT: Silver stain profile of purified NTAP-p150 (lane E) on a 5–20% gradient SDS-PAGE gel showing the p150, p60 and p48 subunits of CAF-1 migrating at their corresponding molecular weight (MW). BOTTOM: Immunofluorescence of NTAP-p150 shows that it is localized to replication foci as shown by its colocalization with PCNA.

**Figure 8:**
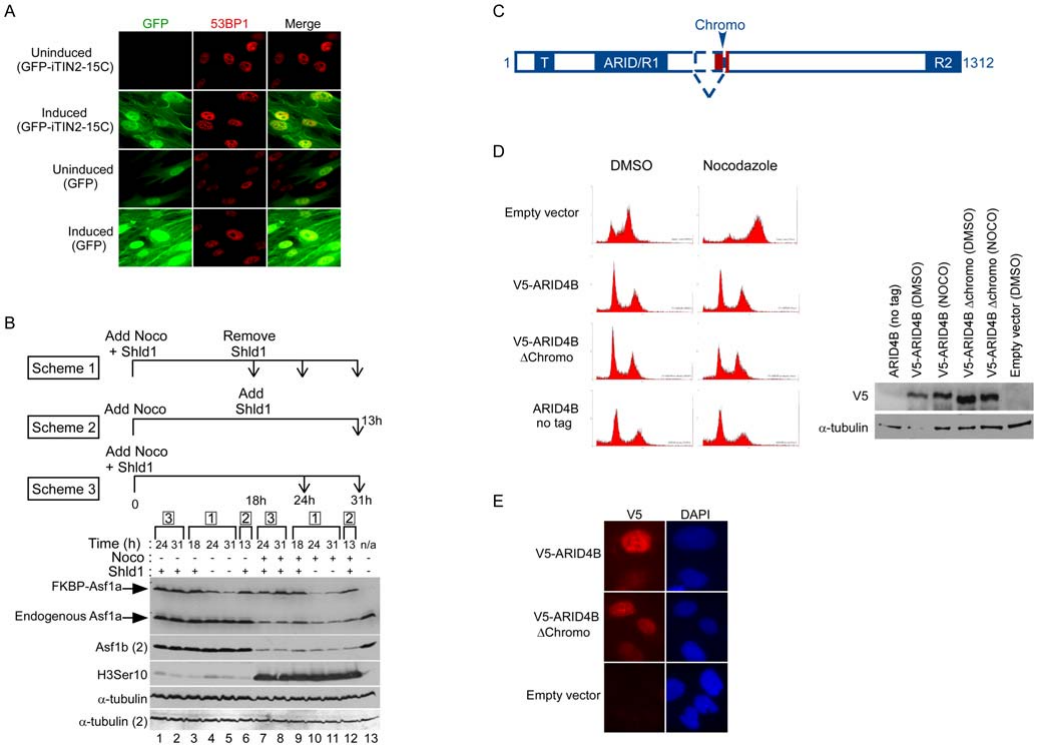
Functional studies using the viral vectors. (A) Inducible expression of GFP-TIN2-15C results in induction of DNA damage. GFP or GFP-iTIN2-15C were cloned into the pLenti CMV/TO Puro vector and transduced in HCA2 T-REx cells. Following a 96 h exposure to 0.1 ug/ml tetracycline, protein induction was monitored by GFP fluorescence and the presence of DNA damage was monitored by staining for 53BP1 foci. (B) Inducible expression or depletion of Asf1a in cells arrested in mitosis using the FKBP-DD system. Three experimental schemes are depicted with the corresponding lanes indicated by brackets. Lanes 1–6: Cells were treated with DMSO as a negative control. Lanes 7–12: Nocodazole treated cells. Lane 13: untreated cells. (C) Functional domains of the ARID4B protein. ARID4B is an 1312 amino acid protein with a Tudor domain (T) at its N-terminus, two repression domains ARID/R1 and R2 [74], and a chromo domain (shown in red). Two splice isoforms have been described and the chromo domain is spliced out in the shorter isoform. (D) The chromo domain of ARID4B is not required for G1-induced growth arrest. U2OS cells were transduced with either an untagged, V5-tagged or V5-tagged minus chromo version of ARID4B recombined in the pQCXP Destination vector. An empty vector was used as a negative control. G1 arrest was monitored by incubating the cells with nocodazole as described in Materials and Methods. The ARID4B Δchromo can still induce a G1 arrest indicating that the chromo domain is not required. On the right is shown the Western blot from the same experiment to monitor expression of each construct. (E) Localization of ARID4B and ARID4B Δchromo to the cell nucleus. The V5-tagged ARID4B isoforms were detected by indirect immunofluorescence using the V5 antibody and an anti-mouse Cy5 secondary antibody. Both isoforms are detected in the nucleus of U2OS cells.

### Examples of RNAi-mediated depletions

We generated an shRNA and two miRNAs against the human histone chaperone Asf1a and expressed them using both lenti- and retro-viral vectors in either U2OS or U2OS T-REx cells. As shown in Figure 6A, Asf1a can be depleted with similar efficiencies using either a miRNA (lanes 2, 3), or shRNA (lane 5), and using either a lentiviral or retroviral backbone (Figure S1A). The efficiency of depletion is affected by several factors, including the efficiency of the shRNA/miRNA, the mRNA levels, the half-life of the mRNA and protein of interest, and the cell line used. In the case of Asf1a, it took between 48 and 72 h to obtain maximal depletion in U2OS cells (Figure S1A) but some proteins can be maximally depleted in less than 24 h (result not shown). Stegmeier et al. [8] reported that more efficient depletions could be accomplished when a GFP cDNA was inserted between the CMV promoter and the miR-30 cassette compared to direct transcription of the miR-30 cassette from the CMV promoter. In our vector system, we found there was often better depletion when the GFP cDNA was present, although the effect was generally quite modest (Figure S1B, C). Whether this difference is due to the protein being depleted, the viral backbone, or other factors is currently unknown.

We also used our system to test the effectiveness of an shRNA to deplete cells of the DNA repair protein MDC1. The shRNA was cloned into the pENTR/pTER+ vector, recombined with the pLenti X1 GFP-Zeo and viral particles were used to transduce U2OS cells. After 48 h, some cells were transferred to a chamber slide and fixed for immunofluorescence. Fluorescence from the GFP-Zeocin fusion protein was used to identify the transduced cells while an antibody was used to detect MDC1. As shown in Figure 6B, cells transduced by the virus were green and depleted for MDC1 (red), whereas neighboring untransduced cells were not green and contained normal levels of MDC1. Additionally, inducible depletion of MDC1 was also accomplished in diploid fibroblasts as shown in Figure 6C, where MDC1 was depleted in the BJ T-REx cell strain. Therefore, both transformed and non-transformed cell types can be studied with these tools.

Although RNAi-mediated depletion is an extremely useful tool to study protein or RNA function, residual amounts of proteins targeted by RNAi were detected in many cases. For example, we generated an inducible VA13 cell line to test two shRNAs against the DNA repair protein XPG. As shown in Figure S1D, although the R6 shRNA was more efficient than the R4 shRNA to deplete XPG (lanes 2 and 4), residual XPG could be detected. We confirmed that the residual band is indeed XPG by demonstrating its absence in the XPG-null cell line 94RD270 (lane 6).

### Examples of overexpressed proteins

A major advantage of the various Destination vectors is that a combination of cDNAs/shRNAs/miRNAs can be expressed in the same cell, in either a constitutive or inducible manner. We generated VA13 cell lines that inducibly express the human histone chaperones Asf1a, Asf1b or HIRA or the combinations Asf1a/HIRA or Asf1b/HIRA. As shown in Figure 7A, very little expression of HA-Asf1a, HA-Asf1b and V5-HIRA was detected without induction, but abundant overexpression of each protein was detected 48 h after induction. In Figure 7B, inducible expression of XPG-V5 is compared by Western blotting and immunofluorescence. In each case, a minority of cells (<1%) showed constitutive expression or lack of induction.

We also tested whether overexpressed proteins from our lentiviral vectors could be assembled into functional multisubunit complexes. We fused the NTAP tag to the N-terminus of the p150 subunit of the human chromatin assembly factor 1 (CAF-1), a three subunit (p150, p60, and p48) complex that performs DNA replication-coupled histone deposition [65]. The pLenti CMV/TO NTAP-p150 puro plasmid was constructed, tagging p150 with a streptavidin binding peptide (SBP) and calmodulin binding peptide (CBP) for purification. We transduced HeLa T-REx cells and screened for clones that showed minimal leaky expression and good induction (result not shown). We generated cytosolic (S100) and nuclear extracts (NE) from uninduced and induced cells and purified NTAP-p150 from the NE fraction using streptavidin beads and calmodulin sepharose. As seen in Figure 7C, no expression of the NTAP-p150 is detected in the uninduced cells, even in the pellet from the affinity-purified sample, showing tight control of expression (lanes 1–4, upper band indicated by an arrow, NTAP blot). Upon induction with doxycycline, the majority of the NTAP-p150 is found in the nucleus (NE, lane 6) and >90% can be purified from the extracts (lane 8). By selecting for single colonies we isolated cell lines that express levels of NTAP-p150 that are substantially less than the endogenous p150 levels in the nucleus (p150 blot), minimizing the possibility that the normal function was perturbed. The tagged human CAF-1 was purified to homogeneity using streptavidin beads and calmodulin sepharose and all three subunits of the native CAF-1 complex were obtained, as shown by silver staining (Figure 7C, right). To monitor if the NTAP-p150 was functional, we used commercially available antibodies to the CBP domain of the NTAP tag for immunofluorescence analysis. Consistent with our fractionation result, the NTAP-p150 is found in the nucleus of the cells (Figure 7C, lower panel) where it colocalizes with the replication protein PCNA in S phase cells, as previously shown for endogenous CAF-1 [66]. In cells outside S phase, both NTAP-p150 and CAF-1 show a diffuse nuclear staining (result not shown). These data indicate that fusion to the NTAP tag does not disrupt the replication-linked function of CAF-1, and illustrates the usefulness of these reagents for biochemistry and cell biology studies.

The vectors expressing GFP can be used for live cell studies, as well as for fluorescence-mediated sorting of transduced cells. These vectors include pENTR4-GFP-C1, -C3, -N1, pENTR/pSM2 (CMV-GFP) and pLenti CMV GFP (Table 1, Figures 2 and 3). For example, Asf1a fused to GFP can be detected in live cells (Figure S2A), and live cells monitored for GFP fluorescence using lentiviruses derived from the pLenti X1 Puro/pSM2 (CMV-GFP) are shown in Figure S1B. Co-staining of cells expressing the XPG-V5 fusion protein with GFP in the pLenti CMV GFP backbone is shown in Figure S2A. The GFP-Zeocin fusion protein can also be detected in live cells, although at a lower intensity than unfused GFP (Figure S2E). Figures S1 and S2 show various Destination vector backbones we tested for the FLAG (Figure S2B), GST (Figure S2C), Ubc9 (Figure S1E) and nuclear localization fusions (Figures S2A, S2C).

### Functional Studies

We also tested whether our system could be used to inducibly generate senescence phenotypes in human diploid fibroblasts. Expression of TIN2-15C, a dominant-negative version of the telomere-associated protein TIN2, results in uncapping of telomeres, induction of the DNA damage response, and cellular senescence [67]. We generated the human fibroblast cell strain HCA2 T-REx, with an inducible eGFP-IRES-TIN2-15C (GFP-iTIN2-15C). As shown in Figure 8A, no expression of GFP-iTIN2-15C was detected in the uninduced cells, as determined by GFP fluorescence, and no DNA damage was found, as measured by immunofluorescence detection of foci containing the double-strand break repair protein 53BP1 [68], [69] (upper panels). 53BP1 and MDC1 show a diffuse nuclear staining in the absence of DNA damage, and they form foci at sites of DNA damage ([70], see also Figure 6B for MDC1 staining in undamaged cells). Four days after induction of GFP-TIN2-15C, cells arrest with dysfunctional telomeres [67] and we detect the presence of abundant (and presumably telomeric) DNA damage focal 53BP1 immunostaining. As a negative control, we inducibly expressed GFP alone, and failed to detect any effect on cell proliferation or DNA damage response indicators (Figure 8A, bottom panels). However, 10–15% of the uninduced cells showed constitutive expression of GFP, indicating leakiness of the T-REx system when GFP alone was expressed, something that we did not observe in GFP-iTIN2-15C expressing cells. We suspect that leaky cells were eliminated from the population by the TIN2-15C- induced growth arrest, such that only cells that had tight regulation of GFP-TIN2-15C continued to proliferate under uninduced conditions. We further explored growth-arresting proteins, examining the effects of the cyclin-dependent kinase inhibitors p16 and p21. In the case of p16, which induces a strong senescence growth arrest without causing DNA damage ([71] and Figure S1F), we found that HCA2-T-REx cells did not express any p16 in the uninduced state. In contrast, expression of p21, another senescence inducer normally expressed and tolerated at low levels by human fibroblasts, gave rise to an uninduced leaky population much like eGFP (Figure S1F). Therefore, in some cases, additional rounds of transduction or selection of clones might be required to obtain a cell strain with less leaky transgene expression.

### Proteins can be rapidly induced or depleted in cells arrested in mitosis

Because the inductions described above occur over a time course of hours or days, experiments that require shorter time scales require different strategies. For example, cells arrested with a mitotic inhibitor will often start dying from overexposure to the drug before RNAi can be effective (Eric Campeau, unpublished observation). Therefore, we tested whether we could provide faster temporal control using FKBP destabilization domain fusion proteins, because this degradation can be reversibly and rapidly halted by addition of the protective ligand termed Shld1 to the extracellular media [29], [30]. This method had already been validated in cycling cells, but had not been previously tested in mitotically arrested populations. To test this, we generated HeLa cells expressing FKBP-Asf1a and monitored for either expression or depletion of the fusion protein using two different experimental schemes and one experimental control (Figure 8B, TOP). In the first scheme, HeLa cells were incubated with Shld1 and arrested in mitosis with nocodazole for 18 hours. Shld1 was then removed, and the cells were incubated for an additional 6 or 13 hours (24 h and 31 h time points). In the second scheme, cells were arrested in mitosis for 18 hours and Shld1 was added for 13 hours (13 h time point). As a control, HeLa cells were incubated with Shld1 and nocodazole for the duration of the experiment and time points were taken at 18, 24 and 31 hours. Each time point was also compared to non-arrested cells to assess expression and depletion efficiencies. As shown in Figure 8B lane 9, the FKBP-Asf1a fusion protein could be detected eighteen hours after addition of Shld1 and nocodazole, and most of the cells were arrested in mitosis (Figure S3, panel 9). Removal of Shld1 induced degradation of FKBP-Asf1a with the same efficiency in cycling or mitotically-arrested cells (compare lanes 4, 5 with 10, 11). Next, we tested whether proteins can be inducibly expressed in mitotically-arrested cells using this strategy. Addition of Shld1 to nocodazole-treated cells induced expression of FKBP-Asf1a with the same efficiency as cycling cells (compare lanes 12 and 6). Cells remain arrested for the duration of the experiment with no significant cell death (Figure S3). Importantly, degradation of FKBP-Asf1a required removal of Shld1 from the media, because continuous expression of FKBP-Asf1a was detected in cells incubated in the presence of Shld1 for the duration of the experiment, regardless if they were cycling (lanes 1, 2, 3) or arrested in mitosis (lanes 7, 8, 9). Monitoring the endogenous levels of Asf1a and Asf1b revealed that both are normally down-regulated in mitosis (Figure 8B). With these tools, we also demonstrated that changes in FKBP-Asf1a levels did not interfere with the regulation or phosphorylation of histone H3 at serine 10 (H3Ser10) by the Aurora mitotic kinases [72]. We conclude that these reagents allow rapid control of protein stability, either in cycling or mitotically arrested cells.

### Both ARID4B isoforms can induce G1 arrest

The AT-rich interaction domain 4B protein (ARID4B) is part of the mSin3A histone deacetylase complex and is involved in gene regulation [73]. ARID4B harbors several protein-protein interaction domains: a tudor domain at its N-terminus, two repression domains (ARID/R1 and R2) [74] and one chromo domain (Figure 8C). Two splice isoforms of the ARID4B mRNA are described in EST databases where the chromo domain is spliced out in the shorter isoform (dashed lines). We could detect both mRNA splice isoforms in all cell lines we tested, regardless of the tissue of origin or if the cell was transformed or not (result not shown). Binda et al. [74], [75] demonstrated that overexpression of ARID4B (referred to as BCAA in their articles) results in a growth arrest that resembles cellular senescence. However, they did not determine whether both isoforms were capable of inducing growth arrest. Because chromo domains can bind methylated histones [76] and senescence-related growth arrest is often associated with formation of repressive chromatin structures [48], [77], [78], we tested whether the ARID4B chromo domain was required for the overexpression-associated growth arrest. The ARID4B cDNA was cloned into the pENTR-V5 plasmid and recombined into the pQCXP DEST vector. The shorter isoform was cloned by RT-PCR as described in materials and methods. Both ARID4B isoforms could induce a G1 growth arrest in U2OS cells as shown by the failure of the cells to accumulate in G2/M upon treatment with nocodazole (Figure 8D). We also tested an untagged version of ARID4B, to ensure that the V5 tag was not producing a dominant-negative effect, and an empty pQCXP vector, to monitor a possible toxic effect of the transduction. The untagged ARID4B but not the empty pQCXP vector could also induce growth arrest (Figure 8D). We conclude that both isoforms of ARID4B can arrest cell growth, and that the chromo domain is therefore not required for this activity.

It has been suggested that some breast cancer cells display a cytoplasmic localization of ARID4B, suggesting that perturbation of the normal nuclear localization of this protein [79], [80] is altered during tumorigenesis. We used immunofluorescence to determine the cellular localization of ARID4B isoforms expressed from V5-tagged cDNAs. As shown in Figure 8E, both isoforms of ARID4B are clearly localized to the nucleus in U2OS cells. We also obtained a nuclear localization of both V5-ARID4B isoforms in all cell lines we tested, including primary cells as well as breast and cervical cancer cell lines (result not shown). Together, our data confirm the ability of our vector system to be useful for a variety of functional studies.

In summary, we successfully depleted or overexpressed several proteins involved in cell-cycle regulation, DNA repair, telomere maintenance and chromatin homeostasis. We can now use these cell lines to study various aspects of chromatin dynamics during cell cycle, DNA repair and establishment of senescence. In our hands, the viral vectors efficiently transduced all cell lines tested (human U2OS, HT1080, MCF7, A549, WI38, IMR90, HCA2, BJ, HOP92, HOP62, OVCAR1, HeLa, K562, MDA468, MDA-MB-431, MCF10A and mouse 3T3) and we believe they will also be useful in a wide variety of applications.

### Future implementations and control vectors

The system we describe can easily be modified or integrated with other systems that use the Gateway technology. Other Entry vectors that contain attL1 and attL2 and do not encode ampicillin resistance are compatible with our Destination vectors. Likewise, other Destination vector with attR1 and attR2 sites and without the kanamycin resistance gene can be used in combination with the Entry vectors described here. Therefore, future vectors can easily be added to this collection – for example, if different drug selections, an alternative inducible system, or expression of multiple proteins from a polycistronic lentiviral backbone [6], [81] are required. Although we describe our system for use with mammalian cells, other groups have shown that VSV-G pseudotyped lentiviruses similar to ours can be used to transduce chicken (DT40) and insect cells (SF9) and that the CMV and SV40 promoter are also functional in these cells [6]. Therefore, constructs harboring CMV and SV40 promoters such as the pLenti CMV Blast or Zeo could conceivably be used to express proteins in these cells, although we have not tested that option. In sum, the system we describe is both immediately useful and accommodates the integration of future developments in the field without significant modifications to the system.

### Concluding remarks

Overexpression and depletion of proteins are powerful tools to study cellular processes. The viral vectors described here can certainly facilitate such studies. However, as for any other gene expression system, there are limitations and experiments must be carefully controlled. First, different cell lines can have different transduction efficiencies, therefore measuring transduction efficiencies in each cell line is critical, and can be done using either a GFP reporter or an empty vector and selecting for drug resistance. Second, overexpression or depletion of a protein can have immediate or delayed side-effects. For example, if overexpression of a protein is toxic to the cell, methylation of the promoter can happen and reduce the expression levels of the protein and, after a brief period of slow growth, the cells can recover with almost normal growth. Inducible expression of the toxic protein in a T-Rex cell line might be an alternative, which has worked in some, but not all cases (Eric Campeau unpublished result). Therefore, careful monitoring of the cell behavior and freezing of early passage cells could be critical. Furthermore, different cell lines might react differently to overexpression or depletion of a protein, depending on tissue of origin or the status of some stress response pathways such as p53/Rb/p16 for example. Finally, overexpressing or depleting a control protein such as GFP or Luciferase could help monitor side-effects unrelated to the protein of interest, such as overloading of the transcription/translation/shRNA machinery.

## Materials and Methods

### Generation and propagation of the vectors

General cloning techniques were used to generate each of the vectors. Detailed protocols are available upon request. All the lentiviral Destination vectors are derived from the p156RRL-sinPPT-CMV-GFP-PRE/Nhe I vector [82], [83] and all the retroviral Destination vectors are derived from the pQCXI series (Clontech). For the lentiviral vectors, a linker was inserted between the Kpn I and Eco RI sites to clone the drug selection cassettes following the Woodchuck post-transcriptional response element (WPRE). All Destination vectors were propagated in the *E.coli* strain DB3.1 (Invitrogen) under appropriate antibiotic selection (see plasmid maps for details). All Entry vectors were propagated in the *E.coli* strain TOP10F' (Invitrogen) under kanamycin selection. After the LR recombination reaction, lentiviral vectors were propagated in the *E.coli* strain Stbl3 (Invitrogen) and retroviral vectors in TOP10F', all under ampicillin selection, except for the pLenti CMV/TO Zeo DEST (zeocin).

### cDNAs, shRNAs and miRNAs

The HA epitope-tagged Asf1a (NCBI GeneID: 25842) and Asf1b (GeneID: 55723) cDNAs as well as the HIRA (GeneID: 7290) cDNA were a gift from Peter Adams (Beatson Institute, Glasgow). The HA-Asf1a and HA-Asf1b were subcloned in the pENTR1A no ccdB plasmid and the HIRA cDNA was cloned into the pENTR4-V5 plasmid. The XPG (GeneID: 2073) cDNA cloned in to the pENTR3C vector with a V5 epitope and a GFP-fusion at its C-terminus (XPG-V5-GFP) was a gift of Ely Kwoh. The XPG-V5 was subcloned into pENTR3C to remove the GFP fusion. The Ubc9 (GeneID: 7329) cDNA was a gift from Claude Gazin (CNRS, UMR217). The ARID4B (GeneID: 51742) cDNA was obtained from the Kazusa cDNA project (clone HH11923, Accession #AB210032). The ARID4B cDNA was cloned in the pENTR4-V5 plasmid. A region of the cDNA encoding the shorter isoform was amplified by RT-PCR from U2OS cells and subcloned into the pCR2.1 TA cloning plasmid (Invitrogen) and sequence verified. The fragment was excised using the *Bbv* CI*/Hin*d III restriction enzymes and inserted into the *Bbv* CI *+ Hin*d III-digested pENTR4-V5-ARID4B plasmid to generate pENTR4-V5 ARID4B Δchromo.

The shRNA for Asf1a was derived from an siRNA previously used [84]. We inserted the following annealed oligonucleotides between the *Bgl II/Hind III* sites of either pENTR/pTER+ or pENTR/pSUPER+:

For Asf1a:

5′GATCCCGTGAAGAATACGATCAAGTGTGTGCTGTCCACTTGATCGTATTCTTCACTTTTTGGAAA and 5′AGCTTTTCCAAAAAGTGAAGAATACGATCAAGTGGACAGCACACACTTGATCGTATTCTTCACGG, where the underlined sequence is specific for human Asf1a.

For MDC1: 5′GATCCCCCAACATGCAGAGATTGAAATTCAAGAGATTTCAATCTCTGCATGTTGTTTTTGGAAA and 5′AGCTTTTCCAAAAACAACATGCAGAGATTGAAATCTCTTGAATTTCAATCTCTGCATGTTGGGG

For XPG: 5′GATCCCAGAATACATGCGGTGGATTTTCAAGAGAAATCCACCGCATGTATTCTTTTTTGGAAA and AGCTTTTCCAAAAAAGAATACATGCGGTGGATTTCTCTTGAAAATCCACCGCATGTATTCTGG.

For the Asf1a shRNA, we also designed an miRNA-based loop in the shRNA because it was reported to result in better depletion efficiencies [85]. To design the Asf1a miRNA, we used the algorithm from Open Biosystems (www.openbiosystems.com). The following oligos were amplified with the Xho I and Eco RI amplification primers and subcloned into the pENTR/pSM2 vectors according to the manufacturer's protocol:

5′TGCTGTTGACAGTGAGCGAGGTCACAAGATTCCACATTAATAGTGAAGCCACAGATGTATTAATGTGGAATCTTGTGACCCTGCCTACTGCCTCGGA

5′TGCTGTTGACAGTGAGCGAAGGTAGAATACTTTCATTATTTAGTGAAGCCACAGATGTAAATAATGAAAGTATTCTACCTCTGCCTACTGCCTCGGA

where the underlined sequences are specific for the human Asf1a.

### LR recombination and purification of the plasmid DNA

The LR recombination was performed using the LR clonase mix (cat. #11791-019, Invitrogen) with 1 µl of miniprep DNA for each of the Entry and Destination vector, 4 µl of TE pH 8.0, 2 µl of LR buffer and 2 µl of LR clonase. Reactions were incubated at room temperature from 2 h to overnight. The proteinase K digestion step was omitted from the manufacturer's protocol and 2 µl of the reaction were used to transform 25 µl of competent *E.coli* Stbl3 or TOP10F' cells generated by the Zymo Research competent cell kit (cat #T-3002). Colonies were picked and inoculated in 50 ml of LB broth. 600 µl were used for a miniprep (Zippy, Zymo research) to confirm the identity of the clone and the remainder of the culture was used for a Qiagen Midi prep according to the manufacturer's instruction for transfecting 293T cells.

### Transfection of 293T cells to generate third generation lentiviruses

The day before the transfection, 5×10^6^ of 293T cells were seeded in a 10 cm dish. Transfection was done with 50 µl of Lipofectamine 2000 (Invitrogen) according to the manufacturer's instructions using 15 µg of the transfer vector, 15 µg of pLP1 (Invitrogen), 6 µg of pLP2 (Invitrogen), and 3 µg of pVSV-G (Invitrogen). The DNA:liposome complex (3 ml) was incubated with the cells in a final volume of 10 ml of OPTI-MEM (Invitrogen) overnight.

### Transfection of 293T cells to generate second generation lentiviruses

The same procedure as above was used except that 20 µg of pCMVΔ8.9 [50] was used instead of the pLP1 and pLP2 vectors.

### Transfection of 293gag/pol cells to generate retroviruses

The day before the transfection, 5×10^6^ of 293 gag/pol cells were inoculated in a 10 cm dish. Lipofectamine 2000 was used for the transfection following the manufacturer's recommendations using 12 µg of the transfer vector, 1.2 µg of pGAG/POL [86] and 1.2 µg of pVSV-G (Invitrogen).

### Collection of viral supernatant and titration of viruses

At 48 h post-transfection, viral supernatants were collected and fresh OPTI-MEM (10 ml) was added to the dish for another collection at 72 h post-transfection. For each collection, viral supernatants were filtered through a 0.2 µm syringe filter. The 48 and 72 h collections were pooled, aliquoted and stored at −80°C. Viral titers were determined by seeding 6-well plates with 2.5×10^4^ HT1080 or HeLa cells and transducing them with 10-fold dilutions (100-1 µl) of viral supernatant as described below. After 12–14 days, cells were fixed in cold methanol and stained with crystal violet solution (0.5% crystal violet, 25% methanol) and counted to determine their colony forming units (cfu). When necessary, viruses were concentrated in a Beckman SW28 rotor at 21,000 rpm for 2 h at 4°C and resuspended in 4 ml of Hank's Buffered Saline solution (HBS, Invitrogen). A second ultracentrifugation in a Beckman 55Ti rotor at 21,000 rpm for 90 minutes was performed and the viral pellet was resuspended in 100 µl of HBS and stored at −80°C in 10 µl aliquots. When measuring viral titers, we take into consideration several factors. First, the health of the 293T packaging cell is critical. If the cells are too confluent or not grown under optimal conditions, it will result in lower viral titers. Similarly, if the cells have been passaged for a long time, it will also yield lower titers. We always thaw a fresh vial of 293T cells when we are producing viruses. Second, the transfection procedure, liposome reagent, the quality of the media used as well as the amounts and quality of the vectors co-transfected are also critical. Third, the viral titers will also be proportional to the volume of media used to grow the cells in order to collect the viral supernatant. Finally, the cell line used for the titration, the amount of cells plated and the concentration of the selecting drug is also affecting viral titers. Other factors like the protein to be overexpressed or depleted might affect cell growth and viability and result in lower viral titers than expected. We recommend using a control vector expressing GFP to monitor transfection as well as transduction efficiencies.

### Transduction of cell lines and induction with doxycycline

Cells were transduced at a MOI between 0.5 and 1. Viruses and cells were incubated overnight in D-MEM media (Invitrogen) containing 6 µg/ml of polybrene (Sigma) in a final volume of 950 µl for 6-well plates, 5 ml for 10 cm dishes and 11 ml for 15 cm dishes. The next day, the viruses were removed, the cells were rinsed twice with PBS and fresh media was added. For primary cells, a second round of transduction was done. Drug selection was added at 48 h post-transduction. The following concentrations were used: blasticidin: 2.5 µg/ml for WI38, HCA2, BJ cells and 5 µg/ml for the other cells, hygromycin: 100 µg/ml for WI38, HCA2, BJ cells, 200 µg/ml for U2OS cells and 300 µg/ml for HT1080 cells, neomycin: 300 µg/ml for WI38, HCA2, BJ cells and 800 µg/ml for the other cells, puromycin: 0.5 µg/ml for HeLa cells and 2.0 µg/ml for other cells, zeocin: 400 µg/ml for HT1080 cells and 200 µg/ml for other cells. Induction of the cDNA/shRNA/miRNA was typically done by addition of doxycycline at a final concentration of 1.0 µg/ml for 48 h (cDNA) or 96 h (shRNA/miRNA) unless indicated otherwise.

### Generation of the MDC1 antibodies

The N-terminal 318 residues of MDC1 were expressed as a GST fusion in *E.coli* and separated from GST by cleavage with PreScission protease by glutathione sepharose (GE Healthcare) affinity. The domain was further purified by High Q (BioRad) ion exchange, and Superdex75 (GE Healthcare) size exclusion chromatography. Rabbits were immunized and antibodies were purified from sera by affinity chromatography. Both GST and GST-MDC1 N-terminus affinity columns were prepared by incubating glutathione sepharose with *E. coli* expression lysates overnight, washing the beads extensively with borate buffer, crosslinking the protein to the beads using dimethypimelimidate and ethanolamine, and washing with glycine-HCl and phosphate buffer. Sera was incubated with the GST affinity resin for 2 hours and then with the GST-MDC1 N-terminus affinity resin overnight. After extensive washes with PBS, antibody was eluted with 200 mM glycine-HCl pH 2.5 and immediately neutralized with 1 M K_2_HPO_4_. Antibody pools were dialyzed into PBS and stored at −80°C.

### Other antibodies, protein electrophoresis, immunofluorescence

The following antibodies were used: anti-V5 (Invitrogen, R96025) at 1∶1000 for Western blot and 1∶1200 for immunofluorescence; anti-Asf1a (C6E10, cat#2990, Cell Signaling Technology) at 1∶6000 for Western blot, anti-Asf1b (cat.#2769, Cell Signaling Technology) at 1∶500 for Western blot, anti-HA (mouse monoclonal 12CA5) at a final concentration of 1 µg/ml for Western blot, anti-XPG [87], at 1∶500 for Western blot, anti-p16 (Neomarkers, JC8) at 1∶500 for immunofluorescence, anti-p21 (BD Biosciences, 556430) at 1∶500 for immunofluorescence and anti-53BP1 (Bethyl Laboratories, BL182) at 1∶2000 for immunofluorescence anti-GFP antibody (Abcam, Ab1218) at 1∶1000 for Western blot, anti-GST (GE healthcare, 27-4577-01) at 1∶100 for Western blot, anti-CBP (ICL Labs, RCBP-45A at 1∶2000 for Western blot and 1∶500 for immunofluorescence, anti-p150 (ss48, [88]) at 1∶1000 for Western blot, anti-PCNA clone PC10 [89] at 1∶500 for immunofluorescence, α-tubulin hybridoma (DM1A, [90]), 1∶2000 for Western blot, anti-H3Ser10 clone 3H10 (Upstate, 05-806) at 1∶2000 for Western blot, anti-c-myc 9E10 (Santa Cruz biotechnologies, sc-40) at 1∶1000 for Western blot and 1∶400 for immunofluorescence. Generation of cell extracts, protein electrophoresis, Western transfer and antibody detection were done using standard procedures. For immunofluorescence, cells were fixed in 4% paraformaldehyde in the case where GFP fluorescence was examined and 100% cold methanol in all other cases. Immunofluorescence was done as described before [91], and images were acquired on an Olympus BX60 fluorescence microscope with the spotfire 3.2.4 software (Diagnostics Instruments) and further processed with Photoshop CS2 (Adobe). For the MDC1 immunofluorescence, images were acquired on a Zeiss Axioskop using a Kodak DC120 digital camera and its capture software and further processed with Photoshop CS2. Images for the NTAP/PCNA, c-myc, V5 were collected on a Zeiss Axioplan 2 microscope with the Axiovision 4.5 software and processed with Photoshop CS3.

### Live cell imaging

Bright field and fluorescent live cell pictures were taken on an Axiovert 200 microscope (Zeiss) using a Qicam fast camera (Qimaging) and the Openlab 4.0.4 software. The pictures were exported to Adobe Photoshop CS for brightness/contrast adjustments.

### CAF-1 purification from HeLa S3 T-Rex-NTAP-p150 cells

Nuclear extracts were prepared from cells induced with 1 µg/mL doxycycline as described [65] followed by a modified TAP method [92]. Briefly, nuclear extracts (400 mM NaCl) were diluted two fold with Buffer A (25 mM Tris-HCl pH 7.5, 1 mM EDTA, 0.1% NP-40, 10% glycerol, 1 mM DTT, 0.1 mM PMSF) to reduce the NaCl concentration to 200 mM final. Extracts were incubated with streptavidin sepharose (GE Healthcare) for three hours, rotating at 4°C. Beads were washed three times with large volumes of Buffer A+200 mM NaCl (A200) and eluted with A200+2 mM biotin [93]. Elutions were bound to calmodulin sepharose (GE Healthcare) for 2 hours in A200+2 mM CaCl_2_. Beads were washed and eluted with A200+5 mM EGTA. Samples were then TCA precipitated and loaded on 5–20% gradient gels and either silver stained or western blotted.

### Shld1 experiment

HeLa cells transduced with the FKBP-Asf1a virus were incubated for 18 hours in the presence of nocodazole (200 ng/ml) or DMSO (0.1%) and Shld1 (1 µM, [30]). Shld1 was removed and both suspension and adherent cells were washed twice with PBS followed by addition of fresh media containing nocodazole. At the 18, 24 and 31 hour time-points, suspension and adherent cells were collected, pooled, and divided in two aliquots for Western and FACS analysis. For reversal of the FKBP-Asf1a degradation, cells were incubated with nocodazole in the absence of Shld1 for 18 hours and Shld1 was added for 13 additional hours. Suspension and adherent cells were collected as above. For FACS, cells were resuspended in 200 µl of PBS and fixed with 800 µl of 70% ethanol. RNAse A (100 µg/ml) and propidium iodide (10 µg/ml) were added to the cells before processing on a FACScalibur system and CellQuest software. FACS profiles were displayed using WinMDI 2.9.

### FACS analysis and G2 trapping assay for ARID4B overexpression

Twenty-four hours after transduction, puromycin and either DMSO (0.1%) or nocodazole (200 ng/ml) were added to the cells and they were incubated for another 20 hours. Mitotic and cycling cells were harvested by collecting the media and trypsinizing the adherent cells. Cells were pooled and divided in two for Western and FACS analysis (performed as above).

#### Availability of the plasmids

All the sequences and maps of the plasmids described here are included in the supplementary materials (Table S1 and File S1). Plasmids will be made available through Addgene (www.addgene.org). We also provided to Addgene different control vectors to monitor transfection and transduction efficiencies and test for possible toxic effects of the viruses, including some insert-less vectors, or vectors encoding cDNAs or hairpins against GFP or luciferase (see Table S1 for details). Detailed protocols, sequences and maps of our vectors and future vectors will be described at http://www.ericcampeau.com.

## Supporting Information

Figure S1 Other vectors tested for RNAi-mediated depletion or cDNA overexpression. (A) Lanes 1–5): time course for the inducible depletion of Asf1a. U2OS T-REx cells transduced with the pLenti X2 Neo/pTER Asf1a shRNA vector were either uninduced (lane 1) or induced with doxycycline for 24, 48, 72 or 96 h (lanes 2–5). Lanes 6–9: comparison of shRNAs and miRNAs for the depletion of Asf1a using different viral backbones and different cell lines. HeLa cells transduced with the pLenti X1 Puro/pSM2(CMV) Asf1a miRNA (lane 6), pLenti X1 Puro/pSM2(CMV-GFP) Asf1a miRNA (lane 7) or U2OS T-REx cells transduced with the pLenti X2 Hygro/pSM2 (CMV) Asf1a miRNA (lane 8) or 293 G/P cells transfected with the pQCXIN X2/pTER Asf1a shRNA (lane 9, 72 h post-transfection) resulted in comparable levels of Asf1a depletion. (B) Depletion of Asf1a using a miRNA in either the SM2 (CMV) or SM2 (CMV-GFP) vector. Live cell images of U2OS cells transduced with either an miRNA against Asf1a (1, 3) or the same miRNA with the GFP protein co-expressed (2, 4). Panels 1 and 2 are brightfield whereas 3 and 4 are the green fluorescence channel. (C) Cells shown in (B) were analyzed by Western blotting to confirm Asf1a depletion. Lane 1: pLenti X1 Puro/pSM2(CMV) Asf1a miRNA; lane 2: pLenti X1 Puro/pSM2(CMV-GFP) Asf1a miRNA; lane 3: untransduced U2OS cell extract; lane 4: U2OS T-REx cells depleted of Asf1b. Lanes 5–6: Inducible depletion of Asf1a using the pLenti X2 Neo/pSM2(CMV/TO) Asf1a miRNA #1 vector. Lane 5 was uninduced whereas lane 6 was induced for 96 h with doxycycline. Lanes 5 and 6 were probed with a different Asf1a antibody (A88, ref) that can detect the various phosphorylated forms of Asf1a. The * indicates a cross-reacting band used as loading control. (D) Inducible and constitutive depletion of the XPG protein with two different shRNAs (R4 and R6) using the pLenti X1 Zeo/pTER backbone in VA13 T-REx cells. Lanes 1 and 3 are the uninduced R4 and R6 shRNA, respectively; lanes 2 and 4 are induced for 96 h. A constitutive depletion of XPG using the R6 shRNA in VA13 cells is shown in lane 5 whereas a cell extract derived from the XPG-null cell line 94RD270 is shown in lane 6 as a negative control. The * indicated a cross-reacting band used as loading control. E) The Asf1a cDNA was inserted into the pENTR-V5-Ubc9 plasmid and recombined into the pQCXP CMV/TO Destination vector. The resulting retrovirus was used to transduce U2OS cells. The blot was probed with an anti-V5 epitope antibody (Left panel), which detected a specific band in the transduced cells (lane 2) when compared to untransduced cells (lane 1). The unstripped blot was reprobed for Asf1a (Right panel) to show the shift in the molecular weight of the Asf1a-Ubc9 fusion protein (lane 4). F) Inducible expression of the p16 and p21 cyclin-dependent kinase inhibitors in HCA2 T-REx cells. Induction of p16 and p21 as in figure 6D were monitored by immunofluorescence using antibodies against p16 and p21 respectively. No cells show leaky p16 expression but a few cells do for p21. However, overexpression of neither protein induces DNA damage as revealed by the absence of 53BP1 foci, even though both can induce senescence (not shown).
(7.43 MB TIF)

Figure S2 (A) Testing of some fusion constructs by immunofluorescence. Panels 1–3: GFP-Asf1a is detected in the nucleus of live cells. Panel 1: GFP channel; panel 2: bright field; panel 3: merged pictures. Panels 4–9: Targeting of the ARID-R1 domain to the nucleus using the pENTR-nuc-myc vector recombined into the pLenti CMV/TO Puro Destination vector. Panel 4: detection of ARID4B-R1 in the nucleus using the myc epitope; panel 5: DAPI staining; panel 6: merged pictures; panel 7: ARID4B-R1 domain fused at the C-terminus to the V5-tag and recombined with the pLenti CMV Neo Destination vector is localized to the cytoplasm as detected using the V5 antibody; panel 8: DAPI staining; panel 9: merged pictures. Western blots of the GFP-Asf1a and the ARID-R1 domain constructs are shown in (B) and (C) respectively. Panels 10–12: Co-localization of the GFP and V5 signals from the pEF-XPG-V5 vector recombined with the pLenti CMV GFP DEST vector. Panel 10: Detection of XPG-V5 with the V5 antibody; panel 11: GFP fluorescence; panel 12: DAPI staining. (B) Western blots of the various Asf1a fusion constructs. Asf1a was cloned into the pENTR-GFP-C1 and pENTR4-FLAG plasmids and recombined with the pLenti CMV/TO and pLenti PGK Puro Destination vectors, respectively. Extracts were blotted incubated with antibodies against Asf1a, FLAG, GFP or α-tubulin. The two bands recognized by the FLAG antibody represent the phosphorylated and unphosphorylated forms of Asf1a where the former cannot be recognized by the Asf1a antibody from Cell Signaling Technologies. (C) Western blots showing expression of the ARID-R1 domain either fused to the nuc-myc (lane 2) or V5 (lane 4) tag at its C-terminus as described in (A). The ARID-R1-nuc-myc was also fused to GST at its N-terminus (lane 6) and the vector was recombined with the pLenti CMV Puro Destination vector. The untransduced HeLa cells are shown in lanes 1, 3 and 5 and were derived from the same blot but not in adjacent lanes to the tested sample. The antibody used for Western blot detection is shown for each blot. (D) Detection of the GFP-Zeocin fusion protein in live cells. Cells expressing the GFP-Zeocin protein can be detected by fluorescence, although the intensity is significantly lower than for GFP alone. TOP: HeLa cells transduced with an empty pLenti X1 GFP-Zeo vector either fluorescent channel (panel 1) or bright field (panel 2); BOTTOM: untransduced HeLa cells, fluorescent channel (panel 3) or bright field (panel 4). (E) Testing of various constructs to express a V5-tagged Luciferase in HeLa cells. The name of the Destination vector is shown on top of the figure. Untransduced HeLa cells, or HeLa cells transduced with a shRNA against Luciferase (shLUC), are used as negative controls. The luciferase is detected using an antibody that recognizes the V5 epitope only in cells transduced with the viruses. Alpha-tubulin is used as a loading control for lanes 1–5 and HP1 gamma for lanes 6–7.
(9.90 MB TIF)

Figure S3 FACS analysis of the depletion or expression of FKBP-Asf1a in mitosis. Samples were collected and analyzed as outlined in Materials and Methods. All samples treated with nocodazole show accumulation in mitosis and samples treated with DMSO show a normal cell cycle profile. No significant cell death occurred during the experiment. The numbers shown with each FACS profile corresponds to the lane number in Figure 8B.
(1.72 MB TIF)

Table S1 Plasmids submitted to Addgene.
(0.04 MB XLS)

File S1 Vector maps and sequences
(5.49 MB ZIP)
