# Supplementary figures and images for: A Versatile Viral System for Expression and Depletion of Proteins in Mammalian Cells
Source: PLoS One. 2009 Aug 6;4(8):e6529. doi: 10.1371/journal.pone.0006529 (PMC2717805; doi:10.1371/journal.pone.0006529)

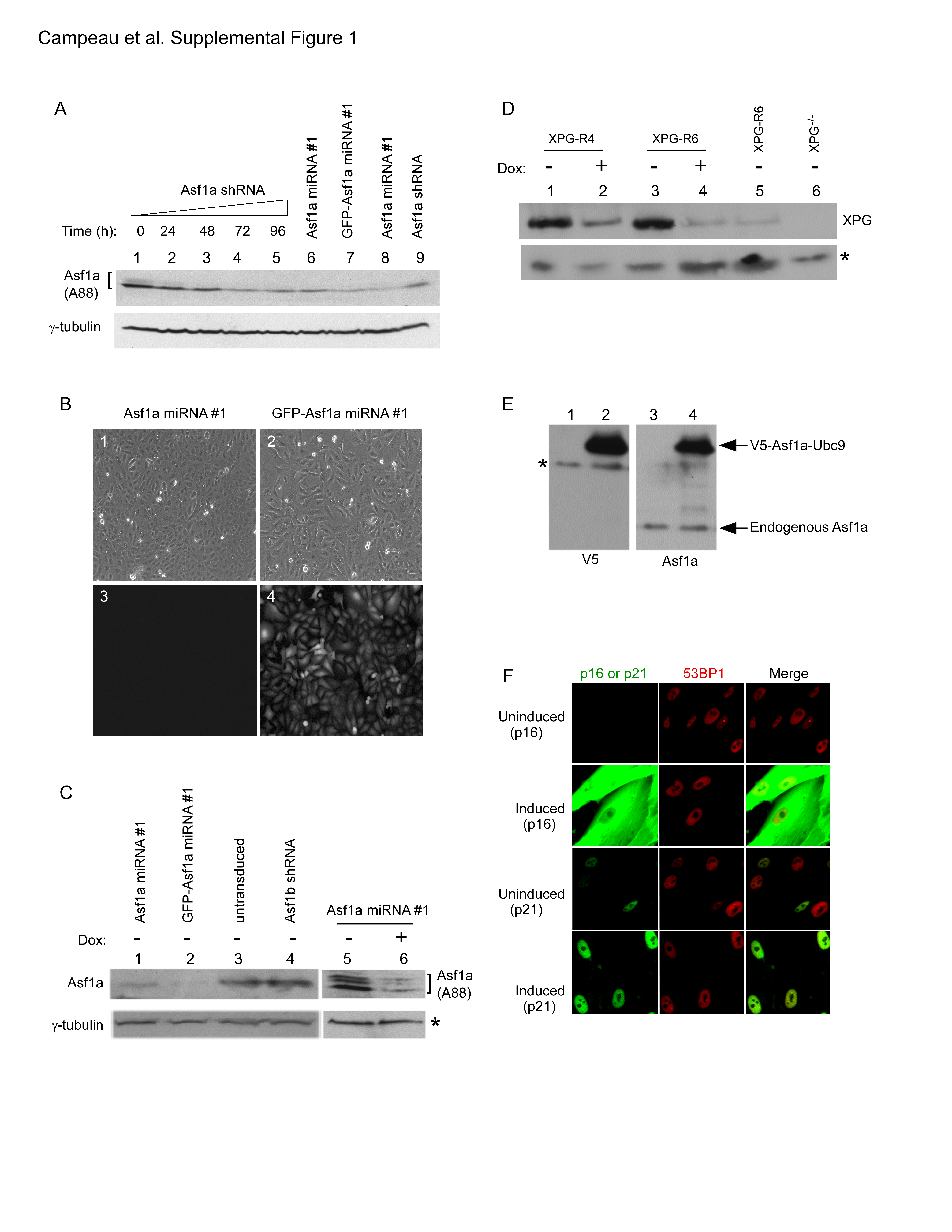

Supplement: Figure S1 — Other vectors tested for RNAi-mediated depletion or cDNA overexpression. (A) Lanes 1–5): time course for the inducible depletion of Asf1a. U2OS T-REx cells transduced with the pLenti X2 Neo/pTER Asf1a shRNA vector were either uninduced (lane 1) or induced with doxycycline for 24, 48, 72 or 96 h (lanes 2–5). Lanes 6–9: comparison of shRNAs and miRNAs for the depletion of Asf1a using different viral backbones and different cell lines. HeLa cells transduced with the pLenti X1 Puro/pSM2(CMV) Asf1a miRNA (lane 6), pLenti X1 Puro/pSM2(CMV-GFP) Asf1a miRNA (lane 7) or U2OS T-REx cells transduced with the pLenti X2 Hygro/pSM2 (CMV) Asf1a miRNA (lane 8) or 293 G/P cells transfected with the pQCXIN X2/pTER Asf1a shRNA (lane 9, 72 h post-transfection) resulted in comparable levels of Asf1a depletion. (B) Depletion of Asf1a using a miRNA in either the SM2 (CMV) or SM2 (CMV-GFP) vector. Live cell images of U2OS cells transduced with either an miRNA against Asf1a (1, 3) or the same miRNA with the GFP protein co-expressed (2, 4). Panels 1 and 2 are brightfield whereas 3 and 4 are the green fluorescence channel. (C) Cells shown in (B) were analyzed by Western blotting to confirm Asf1a depletion. Lane 1: pLenti X1 Puro/pSM2(CMV) Asf1a miRNA; lane 2: pLenti X1 Puro/pSM2(CMV-GFP) Asf1a miRNA; lane 3: untransduced U2OS cell extract; lane 4: U2OS T-REx cells depleted of Asf1b. Lanes 5–6: Inducible depletion of Asf1a using the pLenti X2 Neo/pSM2(CMV/TO) Asf1a miRNA #1 vector. Lane 5 was uninduced whereas lane 6 was induced for 96 h with doxycycline. Lanes 5 and 6 were probed with a different Asf1a antibody (A88, ref) that can detect the various phosphorylated forms of Asf1a. The * indicates a cross-reacting band used as loading control. (D) Inducible and constitutive depletion of the XPG protein with two different shRNAs (R4 and R6) using the pLenti X1 Zeo/pTER backbone in VA13 T-REx cells. Lanes 1 and 3 are the uninduced R4 and R6 shRNA, respectively; lanes 2 and 4 are induced for 96 h. [file pone.0006529.s001.tif]

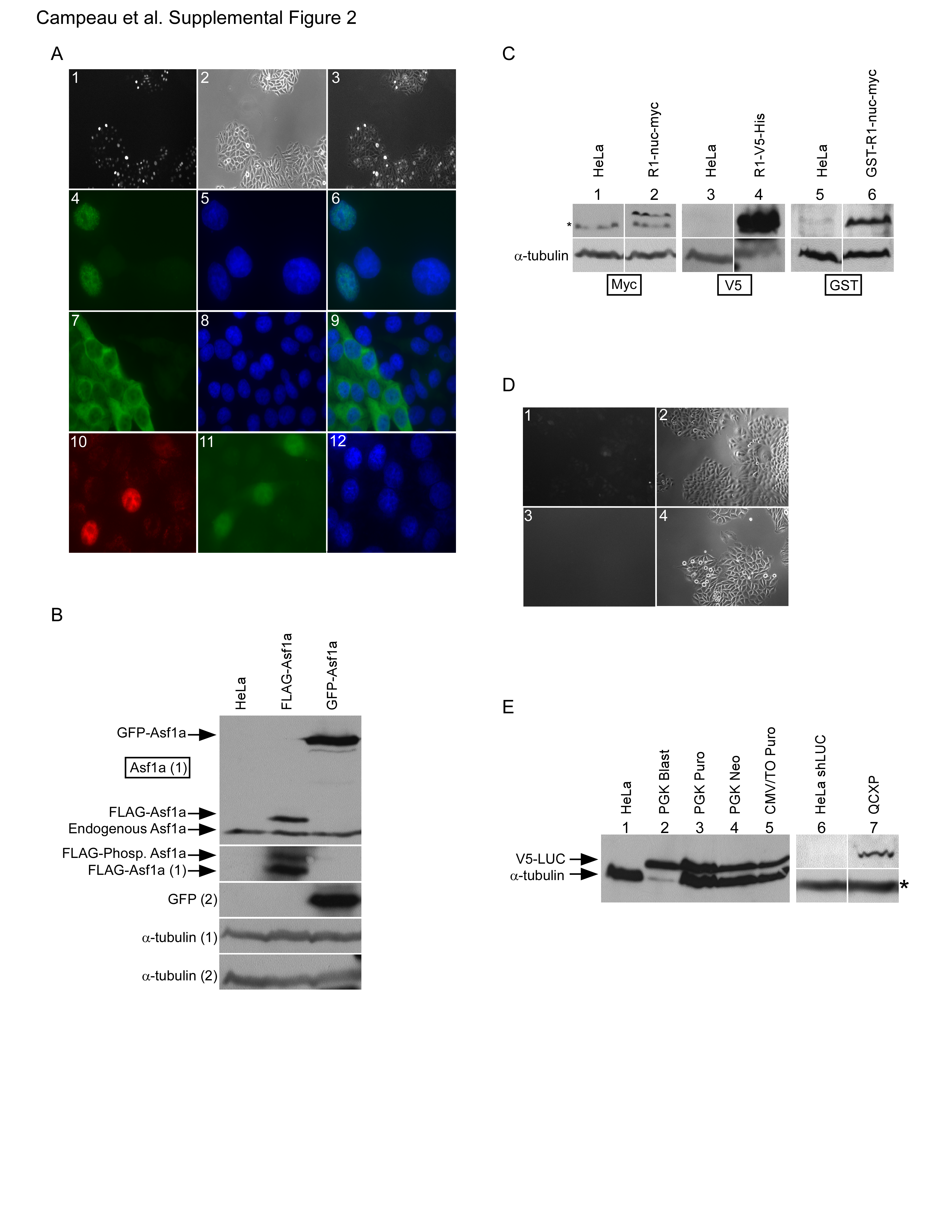

Supplement: Figure S2 — (A) Testing of some fusion constructs by immunofluorescence. Panels 1–3: GFP-Asf1a is detected in the nucleus of live cells. Panel 1: GFP channel; panel 2: bright field; panel 3: merged pictures. Panels 4–9: Targeting of the ARID-R1 domain to the nucleus using the pENTR-nuc-myc vector recombined into the pLenti CMV/TO Puro Destination vector. Panel 4: detection of ARID4B-R1 in the nucleus using the myc epitope; panel 5: DAPI staining; panel 6: merged pictures; panel 7: ARID4B-R1 domain fused at the C-terminus to the V5-tag and recombined with the pLenti CMV Neo Destination vector is localized to the cytoplasm as detected using the V5 antibody; panel 8: DAPI staining; panel 9: merged pictures. Western blots of the GFP-Asf1a and the ARID-R1 domain constructs are shown in (B) and (C) respectively. Panels 10–12: Co-localization of the GFP and V5 signals from the pEF-XPG-V5 vector recombined with the pLenti CMV GFP DEST vector. Panel 10: Detection of XPG-V5 with the V5 antibody; panel 11: GFP fluorescence; panel 12: DAPI staining. (B) Western blots of the various Asf1a fusion constructs. Asf1a was cloned into the pENTR-GFP-C1 and pENTR4-FLAG plasmids and recombined with the pLenti CMV/TO and pLenti PGK Puro Destination vectors, respectively. Extracts were blotted incubated with antibodies against Asf1a, FLAG, GFP or α-tubulin. The two bands recognized by the FLAG antibody represent the phosphorylated and unphosphorylated forms of Asf1a where the former cannot be recognized by the Asf1a antibody from Cell Signaling Technologies. (C) Western blots showing expression of the ARID-R1 domain either fused to the nuc-myc (lane 2) or V5 (lane 4) tag at its C-terminus as described in (A). The ARID-R1-nuc-myc was also fused to GST at its N-terminus (lane 6) and the vector was recombined with the pLenti CMV Puro Destination vector. The untransduced HeLa cells are shown in lanes 1, 3 and 5 and were derived from the same blot but not in adjacent lanes to the tested sample. The antibod [file pone.0006529.s002.tif]

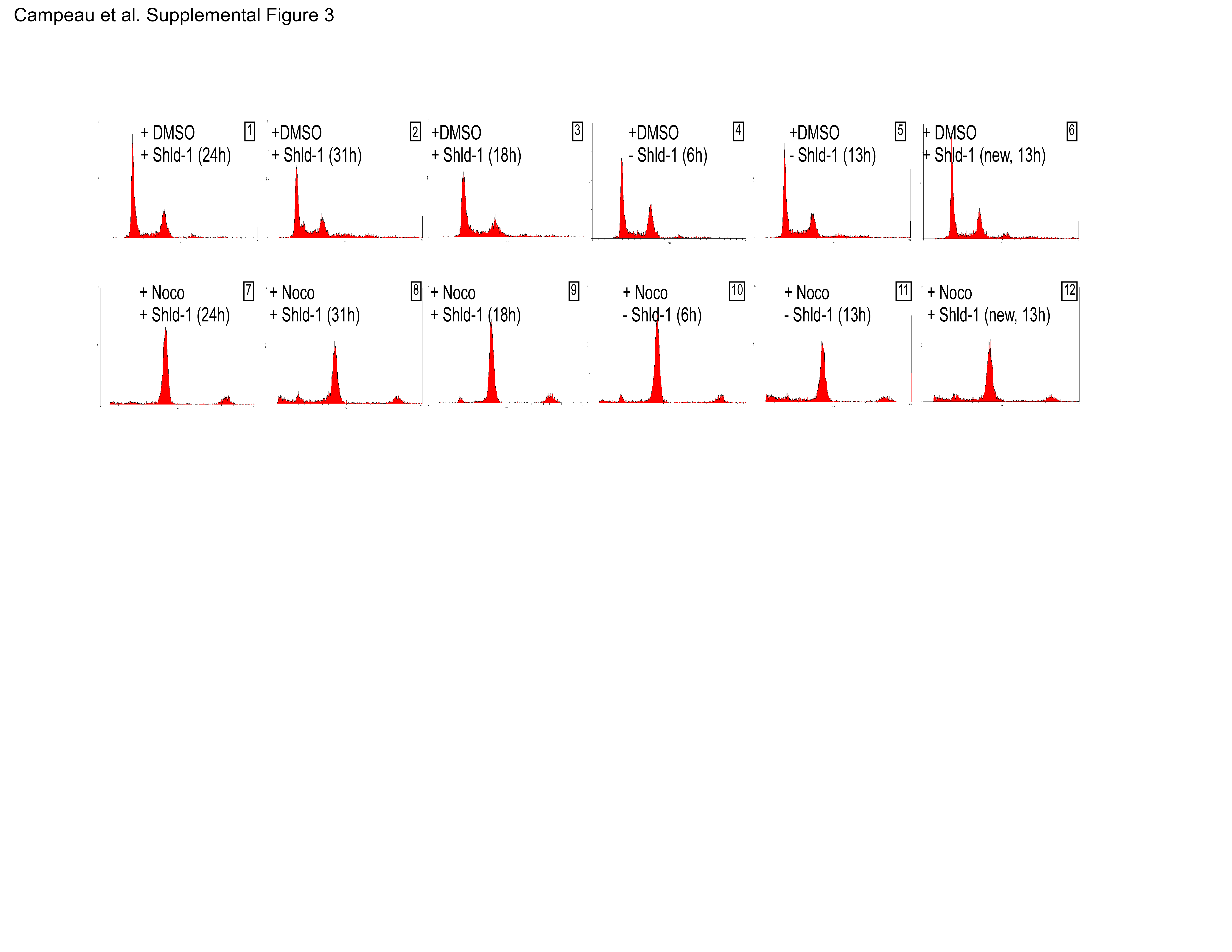

Supplement: Figure S3 — FACS analysis of the depletion or expression of FKBP-Asf1a in mitosis. Samples were collected and analyzed as outlined in Materials and Methods. All samples treated with nocodazole show accumulation in mitosis and samples treated with DMSO show a normal cell cycle profile. No significant cell death occurred during the experiment. The numbers shown with each FACS profile corresponds to the lane number in Figure 8B. (1.72 MB TIF) [file pone.0006529.s003.tif]
