# Supplementary material for: A Versatile Viral System for Expression and Depletion of Proteins in Mammalian Cells
Source: PLoS One. 2009 Aug 6;4(8):e6529. doi: 10.1371/journal.pone.0006529 (PMC2717805; doi:10.1371/journal.pone.0006529)
Supplement: File S1 — Vector maps and sequences (5.49 MB ZIP) [file pone.0006529.s005.zip › pEF-ENTR A, B, C map and MCS.pdf]

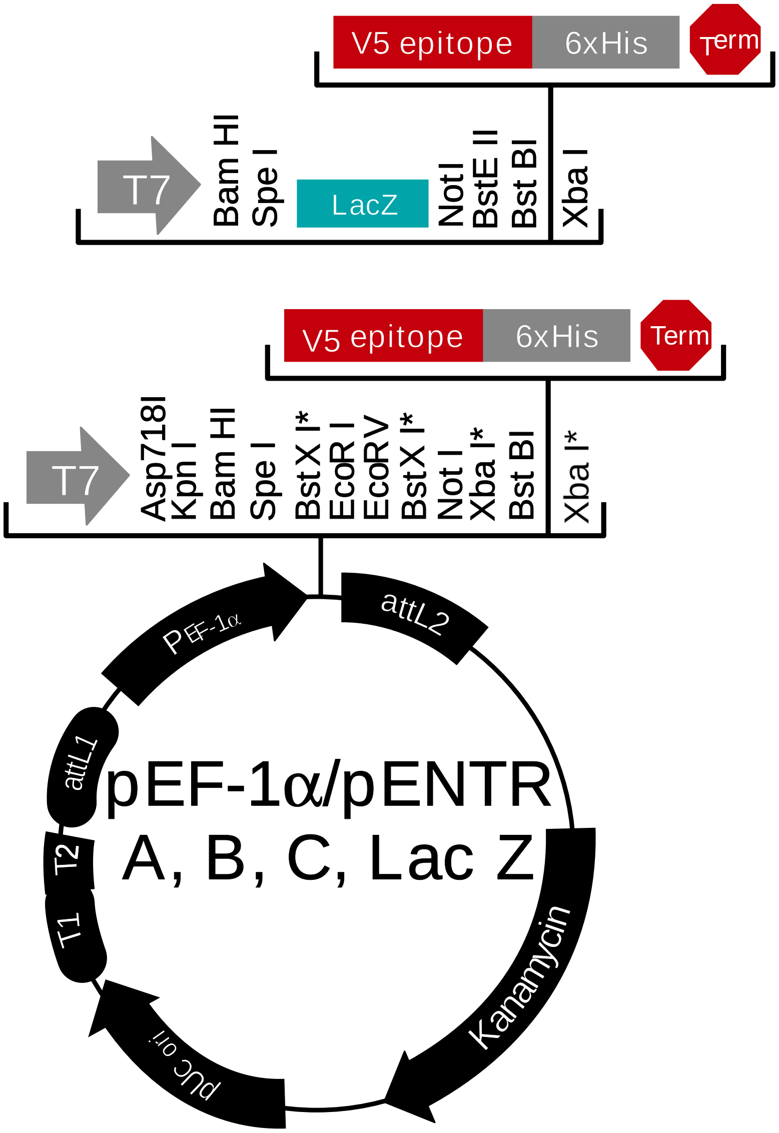

* Site not unique in pEF-1/pENTR A and B

Frame A: 696-6
Frame B: 708-1
Frame C: 709-1
Lac Z: 710-1

Recommended E.coli strain for propagation: TOP10F’

Eric Campeau February 2005

pEF/ENTR A MCS

EF-1α forward priming site; 3´end of hEF-1α Intron 1

GTTTGGATCT TGGTTCATTC TCAAGCCTCA GACAGTGGTT CAAAGTTTTT TTCTTCCATT TCAGGTGTCG TGAGGAATTA

5´ end of hEF-1α Exon 2

T7 promoter/priming site; Asp718 I; Kpn I; Bam HI; Spe I

GCTTGGTACT AATACGACTC ACTATAGGGA GACCCAAGCT GGCTAGTTAA GCT TGG TAC CGA GCT CGG ATC CAC
Trp Tyr Arg Ala Arg Ile His

Bst XI*; EcoR I; EcoR V; BstX I*; Not I; Xba I*

TAG TCC AGT GTG GTG GAA TTC TGC AGA TAT CCA GCA CAG TGG CGG CCG CTC GAG TCT AGA GGG CCC
*** Ser Ser Val Val Glu Phe Cys Arg Tyr Pro Ala Gln Trp Arg Pro Leu Glu Ser Arg Gly Pro

Bst BI; V5 epitope; Polyhistidine region

TTC GAA GGT AAG CCT ATC CCT AAC CCT CTC CTC GGT CTC GAT TCT ACG CGT ACC GGT CAT CAT CAC
Phe Glu Gly Lys Pro Ile Pro Asn Pro Leu Leu Gly Leu Asp Ser Thr Arg Thr Gly His His His

Xba I*; att L2

CAT CAC CAT TGA GTTTATCTAG ACCCAGCTTT CTTGTACAA AGTTGGCATTA TAAGAAAGCA
His His His ***

TTGCTTATCA ATTTGTTGCA ACGAACAGGT CACTATCAGTCAAAATAAAA TCATTATTTG CCATCCAGCT

pENTR reverse primer binding site

GCAGCTCTGG CCCGTGTCTC AAAATCTCTGATGTTACATT

Eric Campeau February 2005

pEF/ENTR B MCS

EF-1α forward priming site; 3´end of hEF-1α Intron 1

GTTTGGATCT TGGTTCATTC TCAAGCCTCA GACAGTGGTT CAAAGTTTTT TTCTTCCATT TCAGGTGTCG TGAGGAATTA

5´ end of hEF-1α Exon 2

T7 promoter/priming site; Asp718 I; Kpn I; Bam HI; Spe I

GCTTGGTACT AATACGACTC ACTATAGGGA GACCCAAGCT GGCTAGTTAA G CTT GGT ACC GAG CTC GGA TCC ACT
Leu Gly Thr Glu Leu Gly Ser Thr

Bst XI*; EcoR I; EcoR V; BstX I*; Not I; Xba I*

AGT CCA GTG TGG TGG AAT TCT GCA GAT ATC CAG CAC AGT GGC GGC CGC TCG AGT CTA GAG GGC CCG
Ser Pro Val Trp Trp Asn Ser Ala Asp Ile Gln His Ser Gly Gly Arg Ser Ser Leu Glu Gly Pro

Bst BI; V5 epitope; Polyhistidine region

CGG TTC GAA GGT AAG CCT ATC CCT AAC CCT CTC CTC GGT CTC GAT TCT ACG CGT ACC GGT CAT CAT CAC
Arg Phe Glu Gly Lys Pro Ile Pro Asn Pro Leu Leu Gly Leu Asp Ser Thr Arg Thr Gly His His His

Xba I*; att L2

CAT CAC CAT TGA GTTTATCTAG ACCCAGCTTT CTTGTACAA AGTTGGCATTA TAAGAAAGCA
His His His ***

TTGCTTATCA ATTTGTTGCA ACGAACAGGT CACTATCAGT CAAAATAAAA TCATTATTTG CCATCCAGCT

pENTR reverse primer binding site

GCAGCTCTGG CCCGTGTCTC AAAATCTCTG ATGTTACATT

Eric Campeau February 2005

pEF/ENTR C MCS

EF-1α forward priming site — 3´end of hEF-1α Intron 1

GTTTGGATCT TGGTTCATTC TCAAGCCTCA GACAGTGGTT CAAAGTTTTT TTCTTCCATT TCAGGTGTCG TGAGGAATTA

5´ end of hEF-1α Exon 2

T7 promoter/priming site — Asp718 I — Kpn I — Bam HI — Spe I

GCTTGGTACT AATACGACTC ACTATAGGGA GACCCAAGCT GGCTAGTTAA GC TTG GTA CCG AGC TCG GAT CCA CTA

Leu Val Pro Ser Ser Asp Pro Leu

Bst XI* — EcoR I — EcoR V — BstX I* — Not I — Bst EII — Bst BI

GTC CAG TGT GGT GGA ATT CTG CAG ATA TCC AGC ACA GTG GCG GCC GCT CGA GGT CAC CCA TTC GAA

Val Gln Cys Gly Gly Ile Leu Gln Ile Ser Ser Thr Val Ala Ala Ala Arg Gly His Pro Phe Glu

V5 epitope — Polyhistidine region

GGT AAG CCT ATC CCT AAC CCT CTC CTC GGT CTC GAT TCT ACG CGT ACC GGT CAT CAT CAC CAT CAC CAT TGA

Gly Lys Pro Ile Pro Asn Pro Leu Leu Gly Leu Asp Ser Thr Arg Thr Gly His His His His His His ***

Xba I* — att L2

GTTT ATCTAG ACCCAGCTTT CTTGTACAA AGTTGGCATTA TAAGAAAGCA

TTGCTTATCA ATTTGTTGCA ACGAACAGGT CACTATCAGTCAAAATAAAA TCATTATTTG CCATCCAGCT

pENTR reverse primer binding site

GCAGCTCTGG CCCGTGTCTC AAAATCTCTGATGTTACATT

Eric Campeau February 2005
