# Supplementary material for: A Versatile Viral System for Expression and Depletion of Proteins in Mammalian Cells
Source: PLoS One. 2009 Aug 6;4(8):e6529. doi: 10.1371/journal.pone.0006529 (PMC2717805; doi:10.1371/journal.pone.0006529)
Supplement: File S1 — Vector maps and sequences (5.49 MB ZIP) [file pone.0006529.s005.zip › pENTR FKBP12DD N and C-term maps and MCS.pdf]

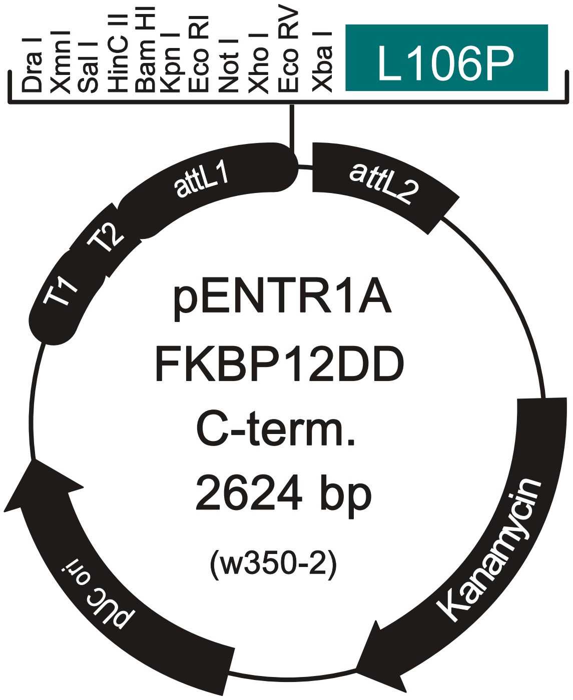

Comments for pENTR 1A FKBP12DD C-term 2624 nucleotides

rrnB T1 transcription termination sequence: bases 106-149
rrnB T2 transcription termination sequence: bases 281-308
att L1:bases 358-457 (complementary strand)
Multiple cloning site (MCS): bases 457-523
FKBP12DD (L106P) domain: 524-847
att L2: bases 853-953
Kanamycin resistance gene: bases 1075-1884
pUC origin: bases 1949-2621

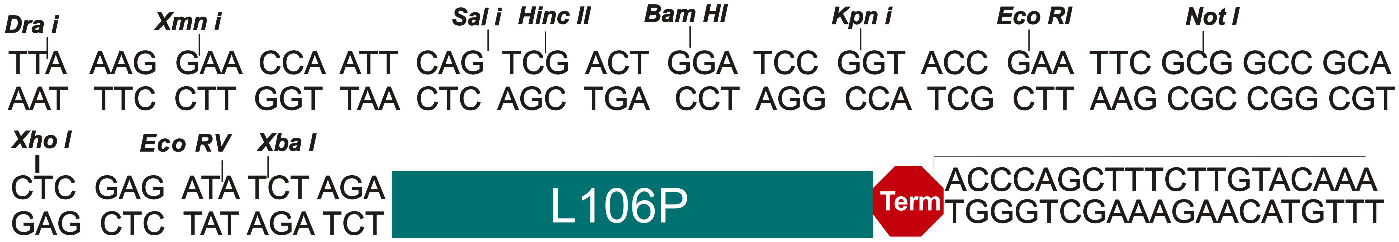

Eric Campeau April 2007

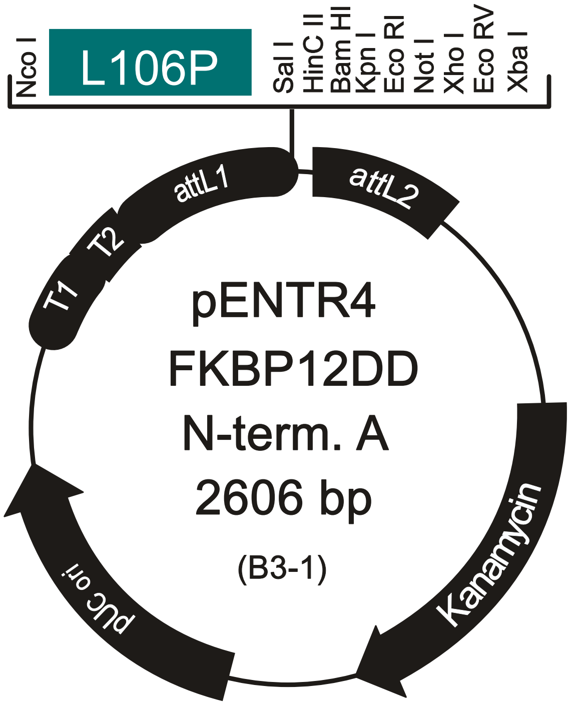

Comments for pENTR4 FKBP12DD N-term A 2606 nucleotides

rrnB T1 transcription termination sequence: bases 106-149
rrnB T2 transcription termination sequence: bases 281-308
att L1:bases 358-457 (complementary strand)
FKBP12DD (L106P) domain: 463-786
Multiple cloning site (MCS): bases 787-834
att L2: bases 835-935
Kanamycin resistance gene: bases 1057-1866
pUC origin: bases 1932-2603

Nco I — *Sal I* — *Hinc II* — *Bam HI* — *Kpn I* — *EcoR I* — *Not I*

ACC ATG G [L106P] GTC GAC TGG ATC CGG TAC CGA ATT CGC
TGG TAC C [L106P] CAG CTG ACC TAG GCC ATG GCT TAA GCG

XhoI — *Eco RV* — *Xba I*

GGC CGC ACT CGA GAT ATC TAG ACCAGCTTTCT TGTACAAA
CCG GCG TGA GCT CTA TAG ATC TGGTCGAAAGA ACATGTTT

Eric Campeau, Brittany Rahmberg July 2007

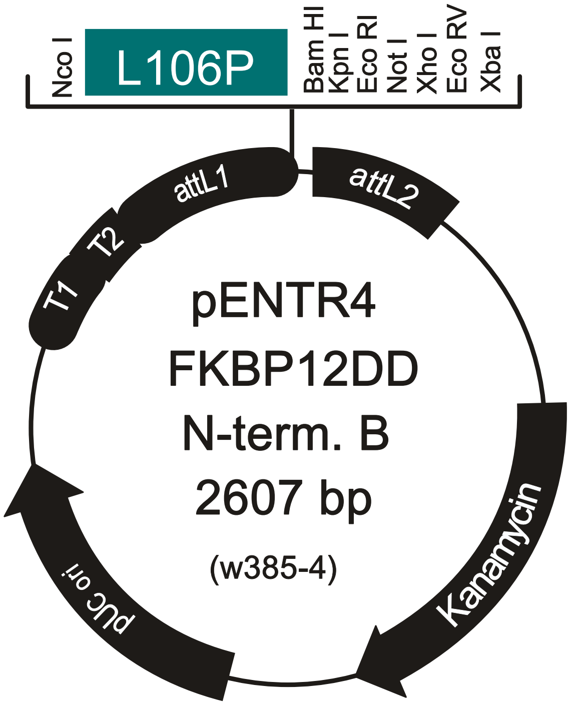

Comments for pENTR4 FKBP12DD N-term B 2607 nucleotides

rrnB T1 transcription termination sequence: bases 106-149
rrnB T2 transcription termination sequence: bases 281-308
attL1:bases 358-457 (complementary strand)
FKBP12DD (L106P) domain: 463-786
Multiple cloning site (MCS): bases 787-835
attL2:bases 836-936
Kanamycin resistance gene: bases 1058-1867
pUC origin: bases 1933-2604

Nco I — *Bam HI* — *Kpn I* — *EcoR I* — *Not I*

ACC ATG G [L106P] **GTC CGA CTG GAT CCG GTA CCG AAT TCG**
TGG TAC C [L106P] **CAG GCT GAC CTA GGC CAT GGC TTA AGC**

XhoI — *Eco RV* — *Xba I*

CGG CCG CAC TCG AGA TAT CTA GACCAGC
GCC GGC GTG AGC TCT ATA GAT CTGGTCG

Eric Campeau July 2007

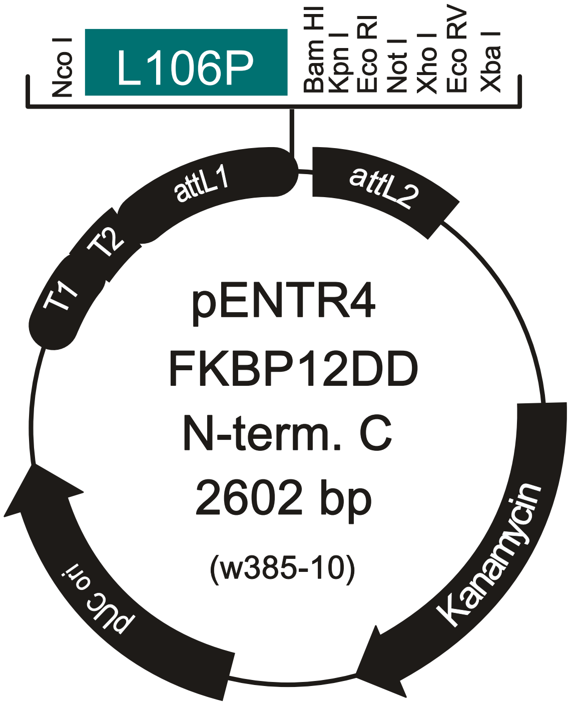

Comments for pENTR4 FKBP12DD N-term C 2602 nucleotides

rrnB T1 transcription termination sequence: bases 106-149
rrnB T2 transcription termination sequence: bases 281-308
*att*L1:bases 358-457 (complementary strand)
FKBP12DD (L106P) domain: 463-786
Multiple cloning site (MCS): bases 786-830
*att*L2:bases 831-931
Kanamycin resistance gene: bases 1053-1862
pUC origin: bases 1928-2599

```
Nco I                                 Bam HI        Kpn I  EcoR I      Not I
ACC ATG G [L106P] GCT GGA TCC GGT ACC GAA TTC GCG GCC
TGG TAC C [L106P] CGA CCT AGG CCA TGG CTT AAG CGC CGG

    XhoI    Eco RV Xba I
GCA CTC GAG ATA TCT AGA  CCAGC
CGT GAG CTC TAT AGA TCT  GGTCG
```

Eric Campeau July 2007
