# Supplementary material for: A Versatile Viral System for Expression and Depletion of Proteins in Mammalian Cells
Source: PLoS One. 2009 Aug 6;4(8):e6529. doi: 10.1371/journal.pone.0006529 (PMC2717805; doi:10.1371/journal.pone.0006529)
Supplement: File S1 — Vector maps and sequences (5.49 MB ZIP) [file pone.0006529.s005.zip › pENTR GFP N and C maps and MCS.pdf]

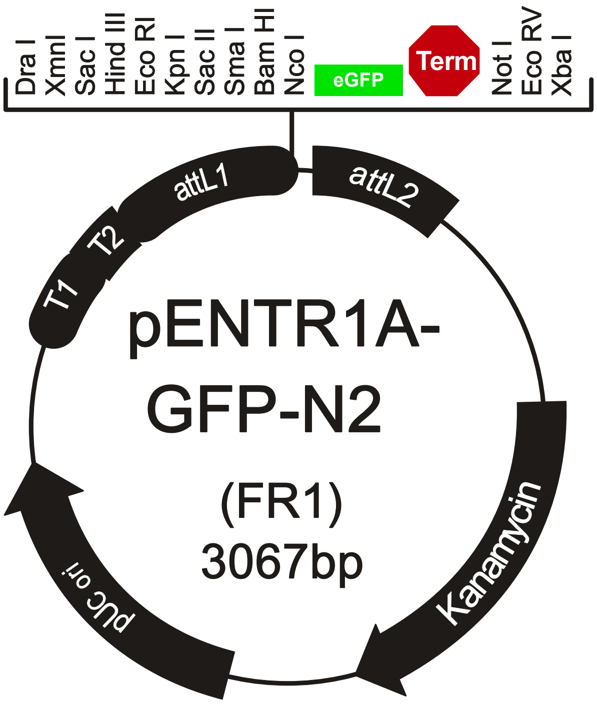

Comments for pENTR1A-GFP-N2
3067 nucleotides

rrnB T1 transcription termination sequence: bases 106-149
rrnB T2 transcription termination sequence: bases 281-308
att L1:bases 358-457 (complementary strand)
enhanced green fluorescent protein (eGFP): bases 552-1271
att L2: bases 1296-1396
Kanamycin resistance gene: bases 1518-2327
pUC origin: bases 2392-3064

```
Dra I          Xmn I          Sal I*          Xho I*      Sac I Hind III   Eco RI
 AAA GGA ACC AAT TCA GTC GAC TGG ATC TCG AGC TCA AGC TTC GAA TTC TGC
                         Tyr Lys Ser Gly Leu Arg Ser Arg Ala Gln Ala Ser

                                       Bam HI
Pst I* Sal I*      Kpn I   Sac II  Apa I Sma I                        Nco I
 AGT CGA CGG TAC CGC GGG CCC GGG ATC CAC CGG CCG GTC GCC ACC ATG GTG
 Asn Ser Ala Val Asp Gly Thr Ala Gly Pro Gly Ser Thr Gly Ser Met

                 Not I      Xho I*  Eco RV  Xba I          attL2
...eGFP  Term AGCGGCCGCACTCGAGATATCTAGACCAGCTTTCTTGTACAAA
```

Recommended *E.coli* strain for propagation: TOP10F'

The position of the genetic elements is approximative.

Made by Francis Rodier (Lawrence Berkeley National Laboratory)

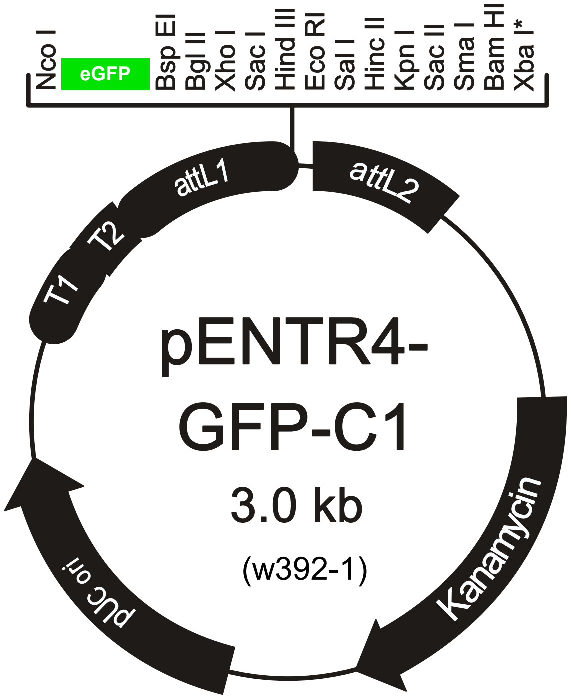

Comments for pENTR4-GFP-C1
3028 nucleotides

rrnB T1 transcription termination sequence: bases 106-149
rrnB T2 transcription termination sequence: bases 281-308
att L1: bases 358-457 (complementary strand)
enhanced green fluorescent protein (eGFP): bases 463-1179
multiple cloning site (MCS): bases 1180-1256
att L2: bases
Kanamycin resistance gene: bases
pUC origin: bases

Recommended *E.coli* strain for propagation: TOP10F’

*Xba I site is methylated. Use methylation deficient strain in order to clone into this site.

Eric Campeau September 2007

pENTR4 -eGFP C1 MCS

```
                              attL1
GGGCCCCAAA TAATGATTTT ATTTTGACTG ATAGTGACCT GTTCGTTGCA ACAAATTGAT
CCCGGGGTTT ATTACTAAAA TAAAACTGAC TATCACTGGA CAAGCAACGT TGTTTAACTA

AAG CAA TGC TTT TTT ATA ATG CCA ACT TTG TAC AAA AAA GCA GGC TCC
TTC GTT ACG AAA AAA TAT TAC GGT TGA AAC ATG TTT TTT CGT CCG AGG

 Nco I                             Bsp EI        Bgl II  Xho I       Sac I  Hind III
ACC ATG G ...eGFP... TAC AAG TCC GGA CTC AGA TCT CGA GCT CAA GCT TCG
    Met              Tyr Lys Ser Gly Leu Arg Ser Arg Ala Gln Ala Ser

Eco RI      Sal I         Kpn I  Sac II  Apa I, Sma I  Bam HI          Xba I
AAT TCT GCA GTC GAC GGT ACC GCG GGC CCG GGA TCC ACC GGA TCT AGA
Asn Ser Ala Val Asp Gly Thr Ala Gly Pro Gly Ser Thr Gly Ser Arg

                                A CCA GCT TTC TTG TAC AAA

                         attL2
GTTGGCATTA TAAGAAAGCA TTGCTTATCA ATTTGTTGCA ACGAACAGGT CACTATCAGT
CAACCGTAAT ATTCTTTCGT AACGAATAGT TAAACAACGT TGCTTGTCCA GTGATAGTCA
                                                 reverse primer binding site
CAAAATAAAA TCATTATTTG CCATCCAGCT GCAGCTCTGG CCCGTGTCTC AAAATCTCTG
GTTTTATTTT AGTAATAAAC GGTAGGTCGA CGTCGAGACC GGGCACAGAG TTTTAGAGAC

ATGTTACATT
TACAATGTAA
```

** Please note that there are no stop codons after the Xba I site.

Eric Campeau September 2007

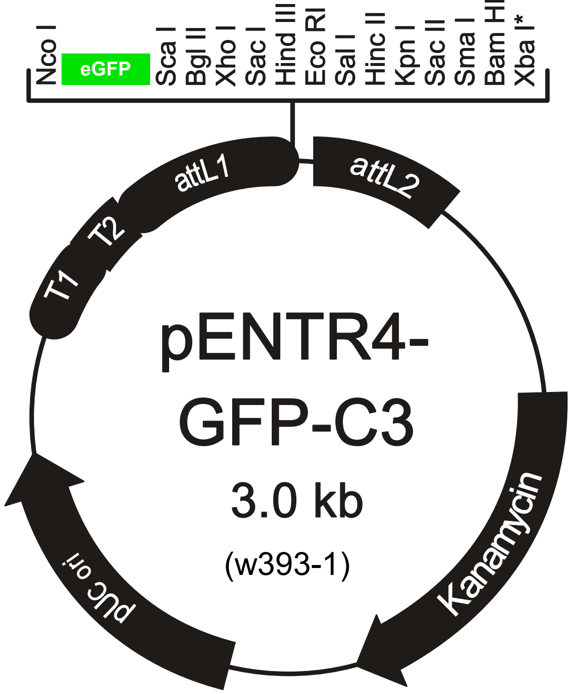

Comments for pENTR4-GFP-C3
3024 nucleotides

rrnB T1 transcription termination sequence: bases 106-149
rrnB T2 transcription termination sequence: bases 281-308
att L1:bases 358-457 (complementary strand)
enhanced green fluorescent protein (eGFP): bases 463-1179
multiple cloning site (MCS): bases 1180-1256
att L2: bases
Kanamycin resistance gene: bases
pUC origin: bases

Recommended *E.coli* strain for propagation: TOP10F’

*Xba I site is methylated. Use methylation deficient strain in order to clone into this site.

Eric Campeau September 2007

pENTR4 -eGFP-C3 MCS

*att*L1

```
GGGCCCCAAA TAATGATTTT ATTTTGACTG ATAGTGACCT GTTCGTTGCA ACAAATTGAT
CCCGGGGTTT ATTACTAAAA TAAAACTGAC TATCACTGGA CAAGCAACGT TGTTTAACTA

AAG CAA TGC TTT TTT ATA ATG CCA ACT TTG TAC AAA AAA GCA GGC TCC
TTC GTT ACG AAA AAA TAT TAC GGT TGA AAC ATG TTT TTT CGT CCG AGG
```

Nco I, Sca I, Bgl II, Xho I, Sac I, Hind III, Eco RI

```
ACC ATG G ...eGFP... TAC AAG TAC TCA GAT CTC GAG CTC AAG CTT CGA
    Met              Tyr Lys Tyr Ser Asp Leu Glu Leu Lys Leu Arg
```

Sal I, Kpn I, Sac II, Apa I, Sma I, Bam HI, Xba I

```
ATT CTG CAG TCG ACG GTA CCG CGG GCC CGG GAT CCA CCG GAT CTA GA
Ile Leu Gln Ser Thr Val Pro Arg Ala Arg Asp Pro Pro Asp Leu

                                A CCA GCT TTC TTG TAC AAA
```

*att*L2

```
GTTGGCATTA TAAGAAAGCA TTGCTTATCA ATTTGTTGCA ACGAACAGGT CACTATCAGT
CAACCGTAAT ATTCTTTCGT AACGAATAGT TAAACAACGT TGCTTGTCCA GTGATAGTCA
```

reverse primer binding site

```
CAAAATAAAA TCATTATTTG CCATCCAGCT GCAGCTCTGG CCCGTGTCTC AAAATCTCTG
GTTTTATTTT AGTAATAAAC GGTAGGTCGA CGTCGAGACC GGGCACAGAG TTTTAGAGAC

ATGTTACATT
TACAATGTAA
```

*** Please note that there are no stop codons after the Xba I site.

Eric Campeau September 2007
