# Supplementary material for: A Versatile Viral System for Expression and Depletion of Proteins in Mammalian Cells
Source: PLoS One. 2009 Aug 6;4(8):e6529. doi: 10.1371/journal.pone.0006529 (PMC2717805; doi:10.1371/journal.pone.0006529)
Supplement: File S1 — Vector maps and sequences (5.49 MB ZIP) [file pone.0006529.s005.zip › pENTR1A no ccdb (w48-1) map and MCS.pdf]

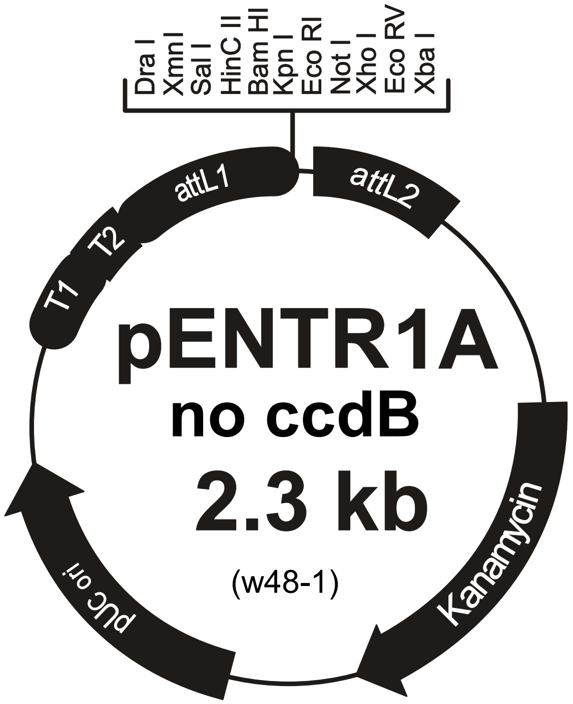

Comments for pENTR 1A no ccdB
2294 nucleotides

rrnB T1 transcription termination sequence: bases 106-149
rrnB T2 transcription termination sequence: bases 281-308
att L1:bases 358-457 (complementary strand)
multiple cloning site (MCS): bases 457-522
att L2: bases
Kanamycin resistance gene: bases
pUC origin: bases

Recommended *E.coli* strain for propagation: TOP10F’

Eric Campeau October 2005

pENTR 1A no ccDB MCS

*att*L1

352 GGGCCCCAAA TAATGATTTT ATTTTGACTG ATAGTGACCT GTTCGTTGCA ACAAATTGAT
CCCGGGGTTT ATTACTAAAA TAAAACTGAC TATCACTGGA CAAGCAACGT TGTTTAACTA

Dra I

412 AAG CAA TGC TTT TTT ATA ATG CCA ACT TTG TAC AAA AAA GCA GGC TTT
TTC GTT ACG AAA AAA TAT TAC GGT TGA AAC ATG TTT TTT CGT CCG AAA

Xmn I　*Sal* I *Hinc II*　*Bam* H I　*Kpn* I　*Eco* R I

460 AAA GGAACC AAT TCA GTC GAC TGG ATC CGG TAC CGA ATT C
TTT CCT TGG TTA AGT CAG CTG ACC TAG GCC ATG GCT TAA G

Not I　*Xho* I　*Eco* RV *Xba* I

GC GGC CGC CGC ACT CGA GAT ATC TAG ACC CAG CTT TCT TGT ACA AA
CG CCG GCG GCG TGA GCT CTA TAG ATC TGG GTC GAA AGA ACA TGT TT

*att*L2

GTTGGCATTA TAAGAAAGCA TTGCTTATCA ATTTGTTGCA ACGAACAGGT CACTATCAGT
CAACCGTAAT ATTCTTTCGT AACGAATAGT TAAACAACGT TGCTTGTCCA GTGATAGTCA

reverse primer binding site

CAAAATAAAA TCATTATTTG CCATCCAGCT GCAGCTCTGG CCCGTGTCTC AAAATCTCTG
GTTTTATTTT AGTAATAAAC GGTAGGTCGA CGTCGAGACC GGGCACAGAG TTTTAGAGAC

ATGTTACATT
TACAATGTAA
