# Supplementary material for: A Versatile Viral System for Expression and Depletion of Proteins in Mammalian Cells
Source: PLoS One. 2009 Aug 6;4(8):e6529. doi: 10.1371/journal.pone.0006529 (PMC2717805; doi:10.1371/journal.pone.0006529)
Supplement: File S1 — Vector maps and sequences (5.49 MB ZIP) [file pone.0006529.s005.zip › pENTR1A NTAP-A,B,C map and MCS.pdf]

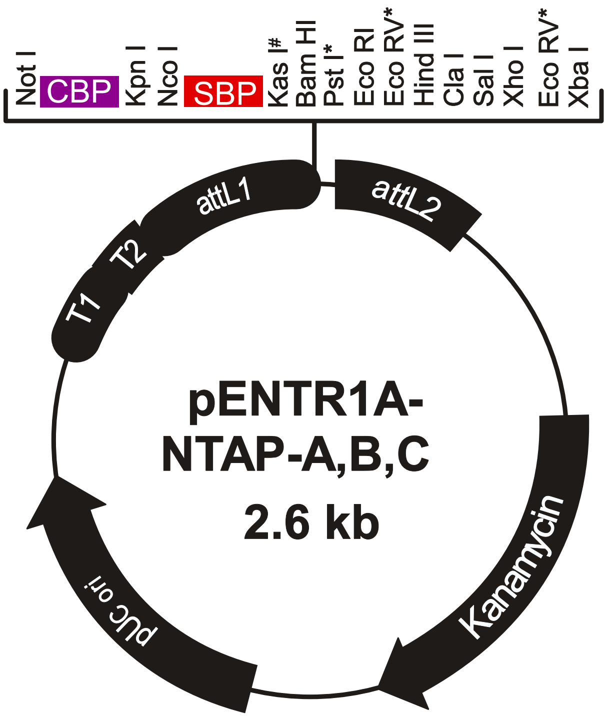

rrnB T1 transcription termination sequence: bases 106-149
rrnB T2 transcription termination sequence: bases 281-308
att L1:bases 358-457 (complementary strand)
CBP tag: 489-566
SBP tag: 585-719
Multiple cloning site (MCS): bases 720-793
att L2: bases 793-892
Kanamycin resistance gene: bases
pUC origin: bases

Recommended *E.coli* strain for propagation: TOP10F’

* Site is not unique
# Site present only in pENTR1A-NTAP A

Eric Campeau April 2007

pENTR 1A NTAP-A MCS (w322-1)

***att*L1**

352 GGGCCCCAAA TAATGATTTT ATTTTGACTG ATAGTGACCT GTTCGTTGCA ACAAATTGAT
CCCGGGGTTT ATTACTAAAA TAAAACTGAC TATCACTGGA CAAGCAACGT TGTTTAACTA

***Dra* I**

412 AAG CAA TGC TTT TTT ATA ATG CCA ACT TTG TAC AAA AAA GCA GGC TTT
TTC GTT ACG AAA AAA TAT TAC GGT TGA AAC ATG TTT TTT CGT CCG AAA

Not* I** ***Kpn I, Nco I

460 AAA GGA ACC CBP SBP
TTT CCT TGG

SBP tag (3´ end)

Kas I BamH I Pst I* EcoR I

P S G G C K L G
CCC TCC GGC GGC TGC AAG CTG GGC GCC CGG GCG GAT CCC CCG GGC TGC AGG AAT TC...

EcoR V* Hind III Cla I Acc I/Sal I Xho I EcoR V* Xba I

.. .G ATA TCA AGC TTA TCG ATA CCG TCG ACC TCG AGA TAT CTA GA ...

CC CAG CTT TCT TGT ACA AA
GG GTC GAA AGA ACA TGT TT

***att*L2**

GTTGGCATTA TAAGAAAGCA TTGCTTATCA ATTTGTTGCA ACGAACAGGT CACTATCAGT
CAACCGTAAT ATTCTTTCGT AACGAATAGT TAAACAACGT TGCTTGTCCA GTGATAGTCA

reverse primer binding site

CAAAATAAAA TCATTATTTG CCATCCAGCT GCAGCTCTGG CCCGTGTCTC AAAATCTCTG
GTTTTATTTT AGTAATAAAC GGTAGGTCGA CGTCGAGACC GGGCACAGAG TTTTAGAGAC

ATGTTACATT
TACAATGTAA

* Site is not unique

# pENTR 1A NTAP-B MCS (w323-1)

***att*L1**

352 GGGCCCCAAA TAATGATTTT ATTTTGACTG ATAGTGACCT GTTCGTTGCA ACAAATTGAT
CCCGGGGTTT ATTACTAAAA TAAAACTGAC TATCACTGGA CAAGCAACGT TGTTTAACTA

***Dra* I**

412 AAG CAA TGC TTT TTT ATA ATG CCA ACT TTG TAC AAA AAA GCA GGC TTT
TTC GTT ACG AAA AAA TAT TAC GGT TGA AAC ATG TTT TTT CGT CCG AAA

Not* I** ***Kpn I, Nco I

460 AAA GGA ACC CBP SBP
TTT CCT TGG

SBP tag (3´ end)

P S G G C K L G

CCC TCC GGC GGC TGC AAG CTG GGC TGC CCG GGC GGA TCC CCC GGG CTG CAG GAA TTC..

BamHI, Pst I*, EcoR I

.. .GAT ATC AAG CTT ATC GAT ACC GTC GAC CTC GAG ATA TCT AGA ...

EcoR V*, Hind III, Cla I, Acc I/Sal I, Xho I, EcoR V*, Xba I

CC CAG CTT TCT TGT ACA AA
GG GTC GAA AGA ACA TGT TT

***att*L2**

GTTGGCATTA TAAGAAAGCA TTGCTTATCA ATTTGTTGCA ACGAACAGGT CACTATCAGT
CAACCGTAAT ATTCTTTCGT AACGAATAGT TAAACAACGT TGCTTGTCCA GTGATAGTCA

reverse primer binding site

CAAAATAAAA TCATTATTTG CCATCCAGCT GCAGCTCTGG CCCGTGTCTC AAAATCTCTG
GTTTTATTTT AGTAATAAAC GGTAGGTCGA CGTCGAGACC GGGCACAGAG TTTTAGAGAC

ATGTTACATT
TACAATGTAA

* Site is not unique

# pENTR 1A NTAP-C MCS (w324-1)

*att*L1

352 GGGCCCCAAA TAATGATTTT ATTTTGACTG ATAGTGACCT GTTCGTTGCA ACAAATTGAT
CCCGGGGTTT ATTACTAAAA TAAAACTGAC TATCACTGGA CAAGCAACGT TGTTTAACTA

Dra I

412 AAG CAA TGC TTT TTT ATA ATG CCA ACT TTG TAC AAA AAA GCA GGC TTT
TTC GTT ACG AAA AAA TAT TAC GGT TGA AAC ATG TTT TTT CGT CCG AAA

Not I *Kpn I, Nco I*

460 AAA GGA ACC CBP SBP
TTT CCT TGG

SBP tag (3´ end)

P S G G C K L G

BamH I Pst I* EcoR I

CCC TCC GGC GGC TGC AAG CTG GGC TTG CCC GGG CGG ATC CCC CGG GCT GCA GGA ATT...

EcoR V* Hind III Cla I Acc I/Sal I Xho I EcoR V* Xba I

.. .CGA TAT CAA GCT TAT CGA TAC CGT CGA CCT CGA GAT ATC TAG A

CC CAG CTT TCT TGT ACA AA
GG GTC GAA AGA ACA TGT TT

*att*L2

GTTGGCATTA TAAGAAAGCA TTGCTTATCA ATTTGTTGCA ACGAACAGGT CACTATCAGT
CAACCGTAAT ATTCTTTCGT AACGAATAGT TAAACAACGT TGCTTGTCCA GTGATAGTCA

reverse primer binding site

CAAAATAAAA TCATTATTTG CCATCCAGCT GCAGCTCTGG CCCGTGTCTC AAAATCTCTG
GTTTTATTTT AGTAATAAAC GGTAGGTCGA CGTCGAGACC GGGCACAGAG TTTTAGAGAC

ATGTTACATT
TACAATGTAA

* Site is not unique
