# Supplementary material for: A Versatile Viral System for Expression and Depletion of Proteins in Mammalian Cells
Source: PLoS One. 2009 Aug 6;4(8):e6529. doi: 10.1371/journal.pone.0006529 (PMC2717805; doi:10.1371/journal.pone.0006529)
Supplement: File S1 — Vector maps and sequences (5.49 MB ZIP) [file pone.0006529.s005.zip › pENTR4 no ccdb (686-1) map and MCS.pdf]

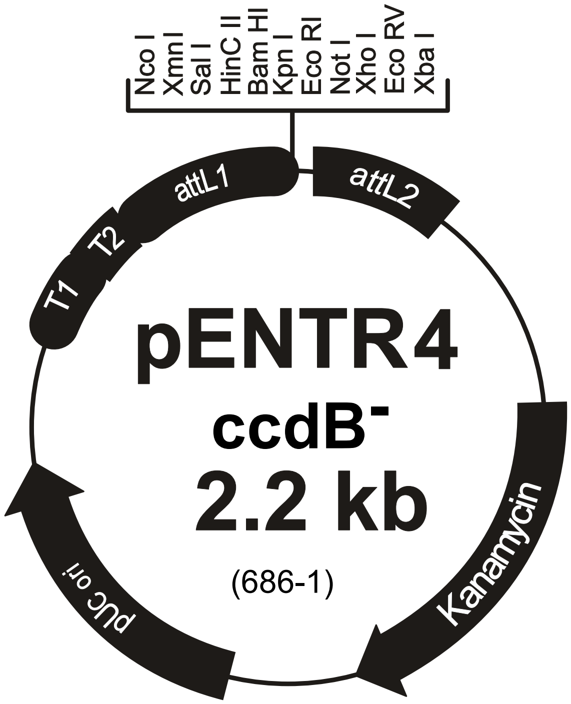

Comments for pENTR4 ccdB$^-$
nucleotides

rrnB T1 transcription termination sequence: bases 106-149
rrnB T2 transcription termination sequence: bases 281-308
*att*L1: bases 358-457 (complementary strand)
multiple cloning site (MCS): bases
*att*L2: bases
Kanamycin resistance gene: bases
pUC origin: bases

Recommended *E.coli* strain for propagation: TOP10F'

Eric Campeau February 2005

pENTR4 -ccDB MCS

***att*L1**

```
GGGCCCCAAA TAATGATTTT ATTTTGACTG ATAGTGACCT GTTCGTTGCA ACAAATTGAT
CCCGGGGTTT ATTACTAAAA TAAAACTGAC TATCACTGGA CAAGCAACGT TGTTTAACTA

AAG CAA TGC TTT TTT ATA ATG CCA ACT TTG TAC AAA AAA GCA GGC TCC
TTC GTT ACG AAA AAA TAT TAC GGT TGA AAC ATG TTT TTT CGT CCG AGG
```

***Nco I* *Xmn*I *Sal*I *HinC II* *Bam*HI *Kpn*I *Eco*R I *Not*I**

```
ACC ATG GGA ACC AAT TCA GTC GAC TGG ATC CGG TAC CGA ATT CGC GGC CGC
TGG TAC CCT TGG TTA AGT CAG CTG ACC TAG GCC ATG GCT TAA GCG CCG GCG
```

Xho*I *Eco RV* *Xba I

```
ACT CGA GAT ATC TAG ACC AGC TTT CTT GTA CAA A
TGA GCT CTA TAG ATC TGG TCG AAA GAA CAT GTT T
```

***att*L2**

```
GTTGGCATTA TAAGAAAGCA TTGCTTATCA ATTTGTTGCA ACGAACAGGT CACTATCAGT
CAACCGTAAT ATTCTTTCGT AACGAATAGT TAAACAACGT TGCTTGTCCA GTGATAGTCA
```

reverse primer binding site

```
CAAAATAAAA TCATTATTTG CCATCCAGCT GCAGCTCTGG CCCGTGTCTC AAAATCTCTG
GTTTTATTTT AGTAATAAAC GGTAGGTCGA CGTCGAGACC GGGCACAGAG TTTTAGAGAC

ATGTTACATT
TACAATGTAA
```
