# Supplementary material for: A Versatile Viral System for Expression and Depletion of Proteins in Mammalian Cells
Source: PLoS One. 2009 Aug 6;4(8):e6529. doi: 10.1371/journal.pone.0006529 (PMC2717805; doi:10.1371/journal.pone.0006529)
Supplement: File S1 — Vector maps and sequences (5.49 MB ZIP) [file pone.0006529.s005.zip › pENTR4-FLAG (w210-2) map and MCS.pdf]

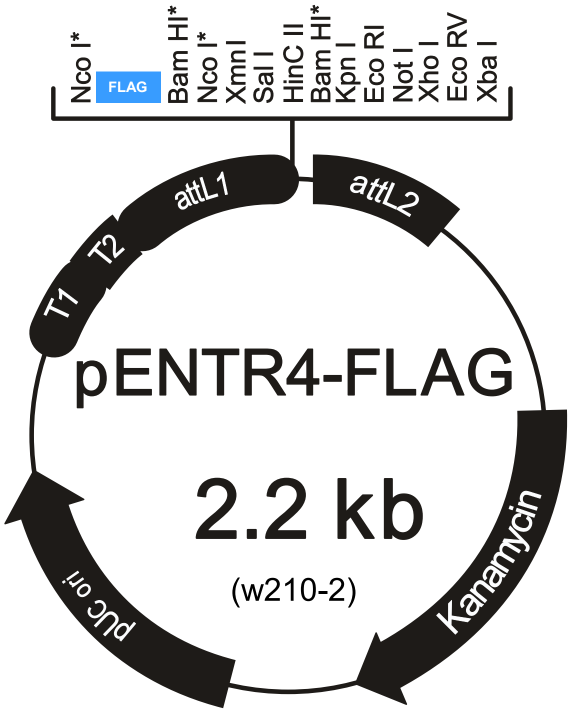

Comments for pENTR4-FLAG
nucleotides

rrnB T1 transcription termination sequence: bases 106-149
rrnB T2 transcription termination sequence: bases 281-308
att L1:bases 358-457 (complementary strand)
multiple cloning site (MCS): bases
att L2: bases
Kanamycin resistance gene: bases
pUC origin: bases

Recommended *E.coli* strain for propagation: TOP10F'

* Sites are not unique

Eric Campeau February 2005

pENTR4 -FLAG MCS

*att*L1

GGGCCCCAAA TAATGATTTT ATTTTGACTG ATAGTGACCT GTTCGTTGCA ACAAATTGAT
CCCGGGGTTT ATTACTAAAA TAAAACTGAC TATCACTGGA CAAGCAACGT TGTTTAACTA

AAG CAA TGC TTT TTT ATA ATG CCA ACT TTG TAC AAA AAA GCA GGC TCC
TTC GTT ACG AAA AAA TAT TAC GGT TGA AAC ATG TTT TTT CGT CCG AGG

*Nco I**　FLAG epitope　BamHI*　*Nco I**

ACC ATG GAT TAC AAG GAC GAT GAC GAC AAA CTC GGA TCC ATG
Met Asp Tyr Lys Asp Asp Asp Asp Lys Leu Gly Ser Met

*Xmn*I　*Sal*I　*HinC II*　BamHI*　*Kpn*I　*Eco*R I　*Not*I

GGA ACC AAT TCA GTC GAC TGG ATC CGG TAC CGA ATT CGC GGC
Gly Thr Asn Ser Val Asp Trp Ile Arg Tyr Arg Ile Arg Gly

*Xho*I　*Eco RV*　*Xba I*

CGC ACT CGA GAT ATC TAG ACC AGC TTT CTT GTA CAA A
Arg Thr Arg Asp Ile *****

*att*L2

GTTGGCATTA TAAGAAAGCA TTGCTTATCA ATTTGTTGCA ACGAACAGGT CACTATCAGT
CAACCGTAAT ATTCTTTCGT AACGAATAGT TAAACAACGT TGCTTGTCCA GTGATAGTCA

reverse primer binding site

CAAAATAAAA TCATTATTTG CCATCCAGCT GCAGCTCTGG CCCGTGTCTC AAAATCTCTG
GTTTTATTTT AGTAATAAAC GGTAGGTCGA CGTCGAGACC GGGCACAGAG TTTTAGAGAC

ATGTTACATT
TACAATGTAA

* Sites not unique

Eric Campeau December 2005
