# Supplementary material for: A Versatile Viral System for Expression and Depletion of Proteins in Mammalian Cells
Source: PLoS One. 2009 Aug 6;4(8):e6529. doi: 10.1371/journal.pone.0006529 (PMC2717805; doi:10.1371/journal.pone.0006529)
Supplement: File S1 — Vector maps and sequences (5.49 MB ZIP) [file pone.0006529.s005.zip › pENTR4-GST-6P-1 (w487-1)map and MCS.pdf]

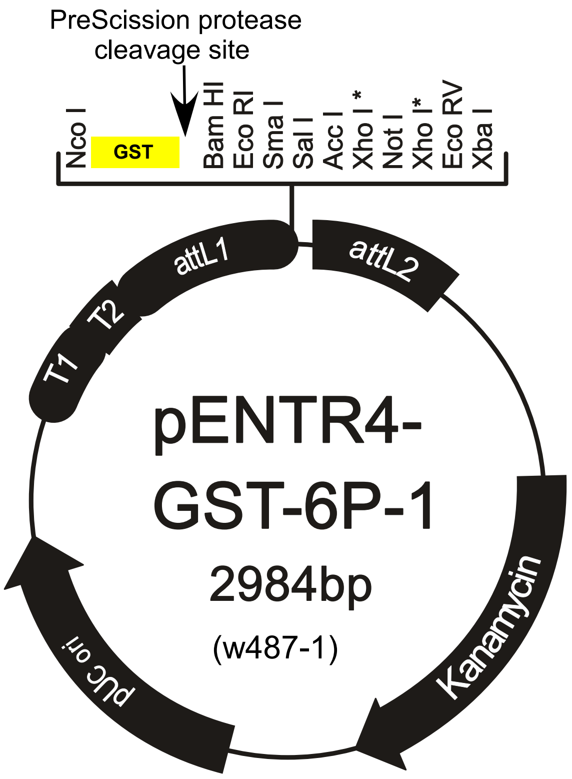

Nco I · PreScission protease cleavage site · Bam HI · Eco RI · Sma I · Sal I, Hinc II

```
ACC ATG .GST. TTC CAG GGG CCC CTG GGA TCC CCG GAA TTC CCG GGT CGA
    Met       Phe Gln Gly Pro Leu Gly Ser Pro Glu Phe Pro Gly Arg
```

Xho I* · Not I · Xho I* · Eco RV · Xba I

```
CTC GAG CGG CCG CAC TCG AGA TAT CTA GACCCAGCTTTCTTGTACAAA
Leu Glu Arg Pro His Ser Arg Tyr Leu
```

Comments for pENTR4-GST-6P-1
2984 nucleotides

rrnB T1 transcription termination sequence: bases 106-149
rrnB T2 transcription termination sequence: bases 281-308
att L1:bases 358-457 (complementary strand)
glutathione S-transferase (GST): bases 463-1149
PreScission protease cleavage site: between bases 1155 and 1156
Multiple cloning site (MCS) bases 1163-1214
att L2: bases 1214-1314
Kanamycin resistance gene: bases 1436-2245
pUC origin: bases 2311-2983

Eric Campeau May 2007
