# Supplementary material for: A Versatile Viral System for Expression and Depletion of Proteins in Mammalian Cells
Source: PLoS One. 2009 Aug 6;4(8):e6529. doi: 10.1371/journal.pone.0006529 (PMC2717805; doi:10.1371/journal.pone.0006529)
Supplement: File S1 — Vector maps and sequences (5.49 MB ZIP) [file pone.0006529.s005.zip › pENTR4-HA-nuc-myc (w557-1) map and MCS.pdf]

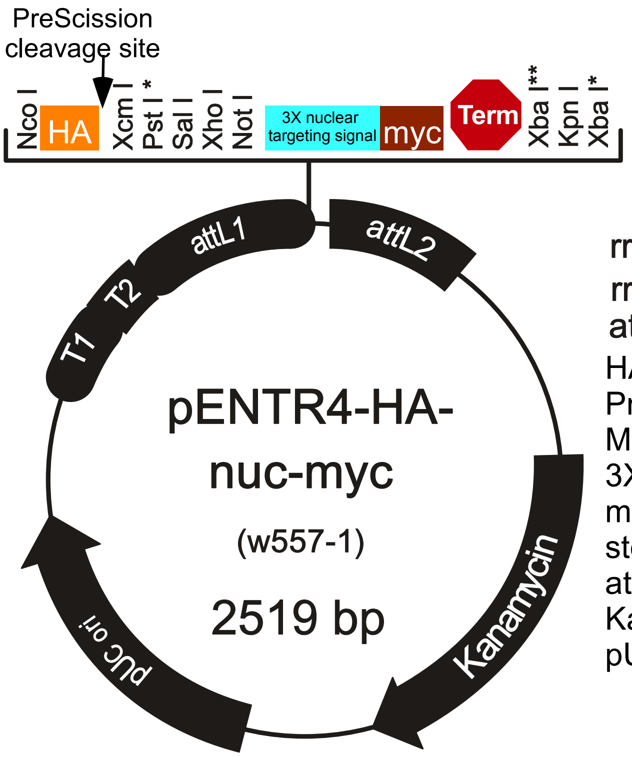

rrnB T1 transcription remination sequence: bases 106-149
rrnB T2 transcription remination sequence: bases 281-308
att L1:bases 358-457 (complementary strand)
HA tag: bases 463-495
PreScission cleavage site: bases 516-517
Multiple cloning site (MCS): bases 523-581
3X nuclear localization signal (SV40): bases 583-654
myc tag: bases 667-696
stop codon: bases 709-711
att L2: bases 746-846
Kanamycin resistance gene: bases 970-1779
pUC origin: bases 1846-2516

HA epitope; PreScission site
Nco I; *Eco 109 I*, *Apa I*
ACC **ATG** GCC TAC CCC TAC GAC GTG CCC GAC TAT GCC AAA CTG GAA GTT CTG TTC CAG GGG
Met Ala Tyr Pro Tyr Asp Val Pro Asp Tyr Ala Lys Leu Glu Val Leu Phe Gln Gly

*Pci I**; *Xcm I*; *Pst I**; *Sal* I; *Xho*
CCC ACA TGT AAC ATG GCC CAG GTG CAG CTG CAG GTC GAC CTC GAG ATC AAA CGG
Pro Thr Cys Asn Met Ala Gln Val Gln Leu Gln Val Asp Leu Glu Ile Lys Arg

Not I; NLS #1; NLS #2
GCG GCC GCA GAT CCA AAA AAG AAG AGA AAG GTA GAT CCA AAA AAG AAG AGA AAG
Ala Ala Ala Asp Pro Lys Lys Lys Arg Lys Val Asp Pro Lys Lys Lys Arg Lys

NLS #3; *myc* epitope
GTA GAT CCA AAA AAG AAG AGA AAG GTA GAT ACG GCC GCA GAA CAA AAA CTC ATC TCA
Val Asp Pro Lys Lys Lys Arg Lys Val Asp Thr Ala Ala Glu Gln Lys Leu Ile Ser

*Xba I***; *Bcl I*; ***Kpn I***
GAA GAG GAT CTG AAT GGG GCC GCA TAG TCTAGAAGCT CGCTGATCCGGTACCGAATT
Glu Glu Asp Leu Asn Gly Ala Ala ***

*Site is not unique
** Site is methylated in original vector (pCMV/myc/nuc)
The position of the genetic elements is approximative
Recommended *E.coli* strain for propagation: TOP10F'

Eric Campeau December 2008
