# Supplementary material for: A Versatile Viral System for Expression and Depletion of Proteins in Mammalian Cells
Source: PLoS One. 2009 Aug 6;4(8):e6529. doi: 10.1371/journal.pone.0006529 (PMC2717805; doi:10.1371/journal.pone.0006529)
Supplement: File S1 — Vector maps and sequences (5.49 MB ZIP) [file pone.0006529.s005.zip › pENTR4-nuc-myc (w556-1)map and MCS.pdf]

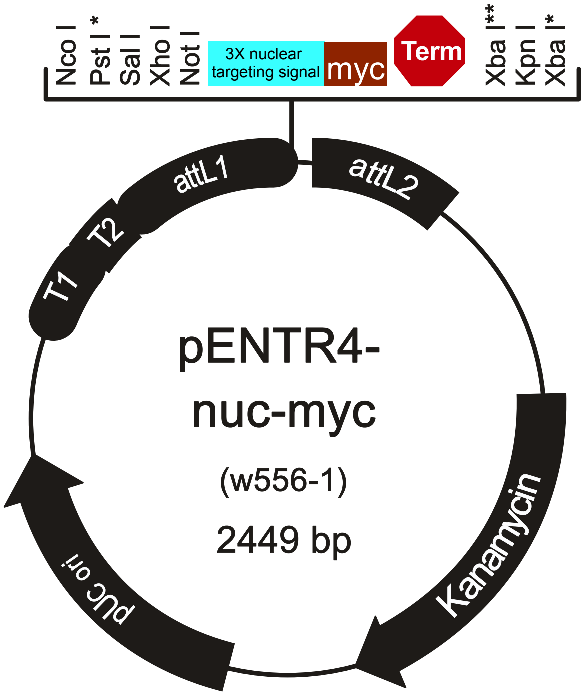

rrnB T1 transcription rermination sequence: bases 106-149
rrnB T2 transcription rermination sequence: bases 281-308
attL1:bases 358-457 (complementary strand)
Multiple cloning site (MCS): bases 461-512
3X nuclear localization signal (SV40): bases 514-585
myc tag: bases 598-627
stop codon: bases 640-642
att L2: bases 679-779
Kanamycin resistance gene: bases 900-1709
pUC origin: bases 1774-2446

Nco I — *Pst I** — *Sal* I — *Xho*

ACC **ATG** GCC CAG GTG CAG CTG CAG GTC GAC CTC GAG ATC AAA CGG
Met Ala Gln Val Gln Leu Gln Val Asp Leu Glu Ile Lys Arg

Not I — NLS #1 — NLS #2

GCG GCC GCA GAT CCA AAA AAG AAG AGA AAG GTA GAT CCA AAA AAG AAG AGA AAG
Ala Ala Ala Asp Pro Lys Lys Lys Arg Lys Val Asp Pro Lys Lys Lys Arg Lys

NLS #3 — *myc* epitope

GTA GAT CCA AAA AAG AAG AGA AAG GTA GAT ACG GCC GCA GAA CAA AAA CTC ATC TCA
Val Asp Pro Lys Lys Lys Arg Lys Val Asp Thr Ala Ala Glu Gln Lys Leu Ile Ser

*Xba I*** — *Bcl I* — ***Kpn I***

GAA GAG GAT CTG AAT GGG GCC GCA TAG TCTAGAAGCT CGCTGATCCGGTACCGAATT
Glu Glu Asp Leu Asn Gly Ala Ala ***

*Site is not unique
** Site is methylated in original vector (pCMV/myc/nuc)
The position of the genetic elements is approximative
Recommended *E.coli* strain for propagation: TOP10F’

Eric Campeau December 2008
