# Supplementary material for: A Versatile Viral System for Expression and Depletion of Proteins in Mammalian Cells
Source: PLoS One. 2009 Aug 6;4(8):e6529. doi: 10.1371/journal.pone.0006529 (PMC2717805; doi:10.1371/journal.pone.0006529)
Supplement: File S1 — Vector maps and sequences (5.49 MB ZIP) [file pone.0006529.s005.zip › pENTR4-V5 (w71-3) map and MCS.pdf]

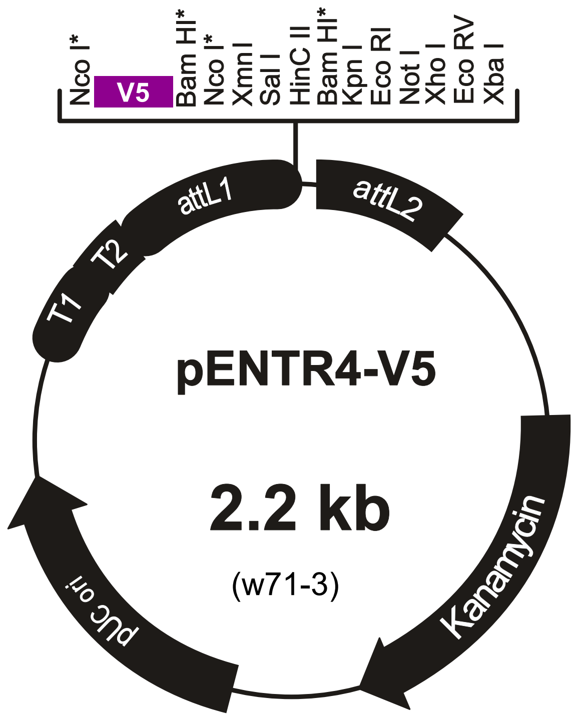

*rrn*B T1 transcription termination sequence: bases 106-149
*rrn*B T2 transcription termination sequence: bases 281-308
*att*L1:bases 358-457 (complementary strand)
multiple cloning site (MCS): bases
*att*L2: bases
Kanamycin resistance gene: bases
pUC origin: bases

Recommended *E.coli* strain for propagation: TOP10F’

* Sites are not unique

Eric Campeau January 2006

pENTR4 -V5 MCS

attL1

GGGCCCCAAA TAATGATTTT ATTTTGACTG ATAGTGACCT GTTCGTTGCA ACAAATTGAT
CCCGGGGTTT ATTACTAAAA TAAAACTGAC TATCACTGGA CAAGCAACGT TGTTTAACTA

AAG CAA TGC TTT TTT ATA ATG CCA ACT TTG TAC AAA AAA GCA GGC TCC
TTC GTT ACG AAA AAA TAT TAC GGT TGA AAC ATG TTT TTT CGT CCG AGG

Nco I* V5 epitope

ACC ATG GAA GGT AAG CCT ATC CCT AAC CCT CTC CTC GGT CTC GAT TCT ACG
Met Glu Gly Lys Pro Ile Pro Asn Pro Leu Leu Gly Leu Asp Ser Thr

BamHI* Nco I* XmnI SalI HinC II BamHI* KpnI EcoR I NotI

GGA TCC ATG GGA ACC AAT TCA GTC GAC TGG ATC CGG TAC CGA ATT CGC GGC
Gly Ser Met Gly Thr Asn Ser Val Asp Trp Ile Arg Tyr Arg Ile Arg Gly

XhoI Eco RV Xba I

CGC ACT CGA GAT ATC TAG ACC AGC TTT CTT GTA CAA A
Arg Thr Arg Asp Ile *

attL2

GTTGGCATTA TAAGAAAGCA TTGCTTATCA ATTTGTTGCA ACGAACAGGT CACTATCAGT
CAACCGTAAT ATTCTTTCGT AACGAATAGT TAAACAACGT TGCTTGTCCA GTGATAGTCA

reverse primer binding site

CAAAATAAAA TCATTATTTG CCATCCAGCT GCAGCTCTGG CCCGTGTCTC AAAATCTCTG
GTTTTATTTT AGTAATAAAC GGTAGGTCGA CGTCGAGACC GGGCACAGAG TTTTAGAGAC

ATGTTACATT
TACAATGTAA

* Sites not unique

Eric Campeau January 2006
