# Supplementary material for: A Versatile Viral System for Expression and Depletion of Proteins in Mammalian Cells
Source: PLoS One. 2009 Aug 6;4(8):e6529. doi: 10.1371/journal.pone.0006529 (PMC2717805; doi:10.1371/journal.pone.0006529)
Supplement: File S1 — Vector maps and sequences (5.49 MB ZIP) [file pone.0006529.s005.zip › pENTR4-V5_2 Ubc9 (w345-2) map and MCS.pdf]

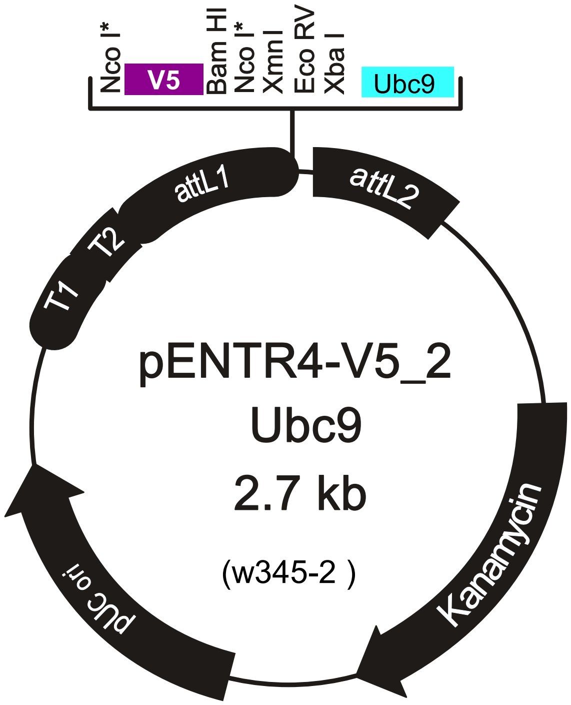

rrnB T1 transcription termination sequence: bases 106-149
rrnB T2 transcription termination sequence: bases 281-308
*att*L1:bases 358-457 (complementary strand)
multiple cloning site (MCS): bases
*att*L2: bases
Kanamycin resistance gene: bases
pUC origin: bases

Recommended *E.coli* strain for propagation: TOP10F'

* Site is not unique

Eric Campeau May 2007

pENTR4 -V5_2 Ubc9 MCS

*att*L1

GGGCCCCAAA TAATGATTTT ATTTTGACTG ATAGTGACCT GTTCGTTGCA ACAAATTGAT
CCCGGGGTTT ATTACTAAAA TAAAACTGAC TATCACTGGA CAAGCAACGT TGTTTAACTA

AAG CAA TGC TTT TTT ATA ATG CCA ACT TTG TAC AAA AAA GCA GGC TCC
TTC GTT ACG AAA AAA TAT TAC GGT TGA AAC ATG TTT TTT CGT CCG AGG

Nco I* V5 epitope

ACC ATG GAA GGT AAG CCT ATC CCT AAC CCT CTC CTC GGT CTC GAT TCT ACG
Met Glu Gly Lys Pro Ile Pro Asn Pro Leu Leu Gly Leu Asp Ser Thr

BamHI ***Nco I**** ***Xmn*I** ***Eco RV*** ***Xba I***

GGA TCC ATG GGA ACC AAT TCA GTC GAG ATA TCT AGA GGT GGC TCT GGT GGC
Gly Ser Met Gly Thr Asn Ser Val Glu Ile Ser Arg Gly Gly Ser Gly Gly

Ubc9 Term CTAGACCCAG CTTTCTTGTA CAAA
GATCTGGGTC GAAAGAACAT GTTT

*att*L2

GTTGGCATTA TAAGAAAGCA TTGCTTATCA ATTTGTTGCA ACGAACAGGT CACTATCAGT
CAACCGTAAT ATTCTTTCGT AACGAATAGT TAAACAACGT TGCTTGTCCA GTGATAGTCA

reverse primer binding site

CAAAATAAAA TCATTATTTG CCATCCAGCT GCAGCTCTGG CCCGTGTCTC AAAATCTCTG
GTTTTATTTT AGTAATAAAC GGTAGGTCGA CGTCGAGACC GGGCACAGAG TTTTAGAGAC

ATGTTACATT
TACAATGTAA

* Site not unique

Eric Campeau May 2007
