# Supplementary material for: A Versatile Viral System for Expression and Depletion of Proteins in Mammalian Cells
Source: PLoS One. 2009 Aug 6;4(8):e6529. doi: 10.1371/journal.pone.0006529 (PMC2717805; doi:10.1371/journal.pone.0006529)
Supplement: File S1 — Vector maps and sequences (5.49 MB ZIP) [file pone.0006529.s005.zip › pENTR_pSM2 series.pdf]

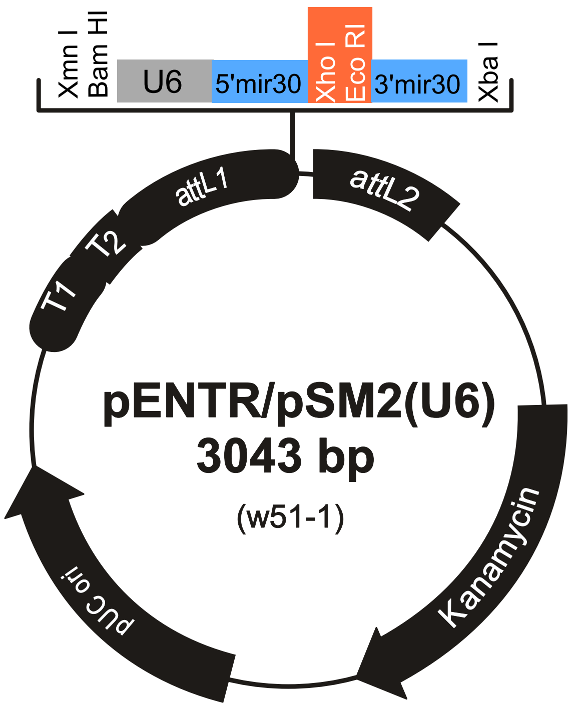

Comments for pENTR/pSM2(U6)
3043 nucleotides

rrnB T1 transcription termination sequence: bases 106-149
rrnB T2 transcription termination sequence: bases 281-308
attL1:bases 358-457 (complementary strand)
U6 RNA promoter: bases 488-989
5'miR30 context: 999-1112
shRNA cloning sites (Xho I/Eco RI):1113-1130
3'miR30 context: 1131-1237
Pol III transcription termination: 1238-1243
attL2: bases 1272-1372
Kanamycin resistance gene: bases 1494-2303
pUC origin: bases 2368-3040

Eric Campeau November 2005

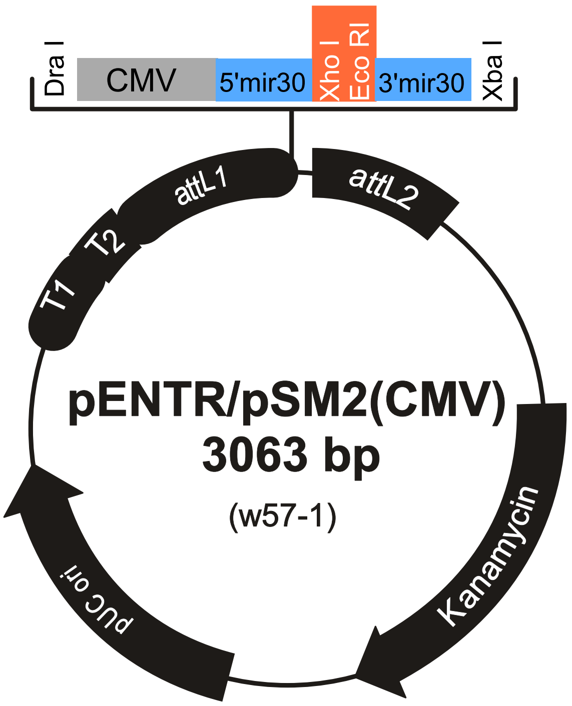

Comments for pENTR/pSM2(CMV)
3063 nucleotides

rrnB T1 transcription termination sequence: bases 106-149
rrnB T2 transcription termination sequence: bases 281-308
attL1:bases 358-457 (complementary strand)
CMV/TO promoter: bases 474-1013
5'miR30 context: 1014-1132
shRNA cloning sites (Xho I/Eco RI):1133-1150
3'miR30 context: 1151-1253
attL2: bases 1292-1392
Kanamycin resistance gene: bases 1514-2335
pUC origin: bases 2407-3060

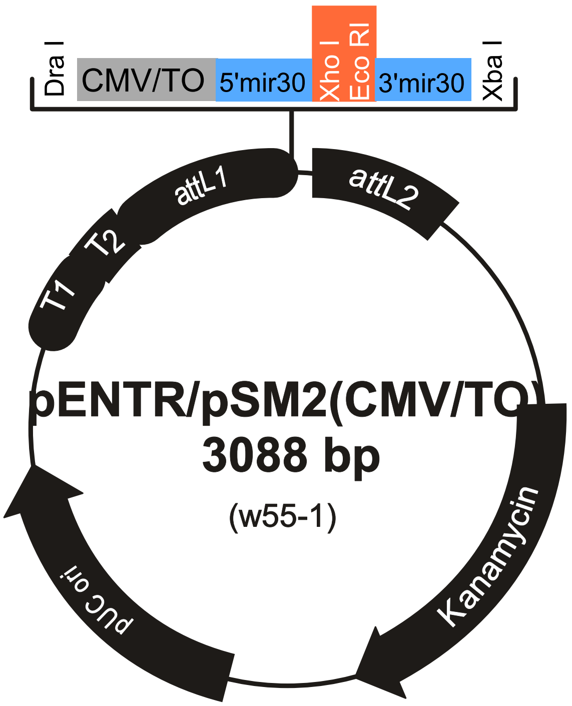

Comments for pENTR/pSM2(CMV/TO)
3088 nucleotides

*rrn*B T1 transcription termination sequence: bases 106-149
*rrn*B T2 transcription termination sequence: bases 281-308
*att*L1:bases 358-457 (complementary strand)
CMV/TO promoter: bases 474-1035
5'miR30 context: 1039-1157
shRNA cloning sites (Xho I/Eco RI):1158-1175
3'miR30 context: 1176-1273
*att*L2: bases 1317-1417
Kanamycin resistance gene: bases 1539-2361
pUC origin: bases 2431-3086

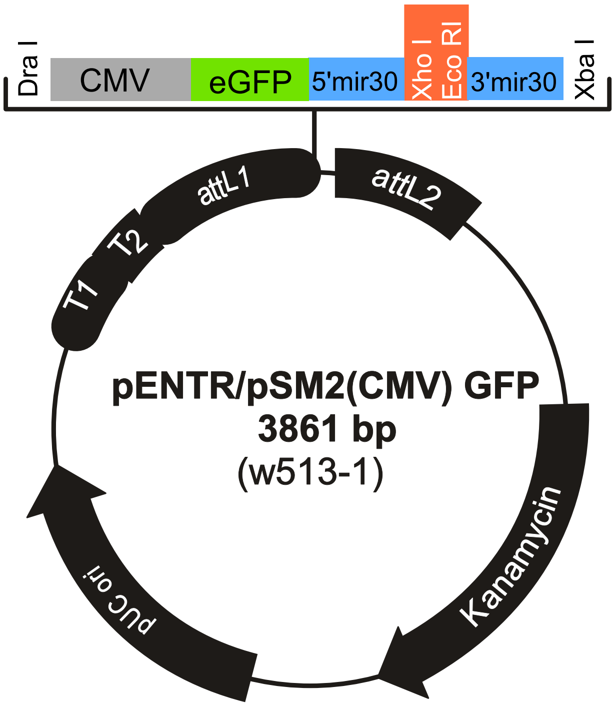

Comments for pENTR/pSM2(CMV) GFP
3861 nucleotides

rrnB T1 transcription termination sequence: bases 106-149
rrnB T2 transcription termination sequence: bases 281-308
attL1:bases 358-457 (complementary strand)
CMV promoter: bases 474-1013
Enhanced green fluorescent protein (eGFP): bases 1083-1802
5'miR30 context: 1813-1930
shRNA cloning sites (Xho I/Eco RI):1931-1948
3'miR30 context: 1949-2051
attL2: bases 2090-2190
Kanamycin resistance gene: bases 2312-3135
pUC origin: bases 3204-3859

Recommended E.coli strain for propagation: TOP10F'

Eric Campeau July 2008
