# Supplementary material for: A Versatile Viral System for Expression and Depletion of Proteins in Mammalian Cells
Source: PLoS One. 2009 Aug 6;4(8):e6529. doi: 10.1371/journal.pone.0006529 (PMC2717805; doi:10.1371/journal.pone.0006529)
Supplement: File S1 — Vector maps and sequences (5.49 MB ZIP) [file pone.0006529.s005.zip › pENTR_pSUPER+ (575-1).pdf]

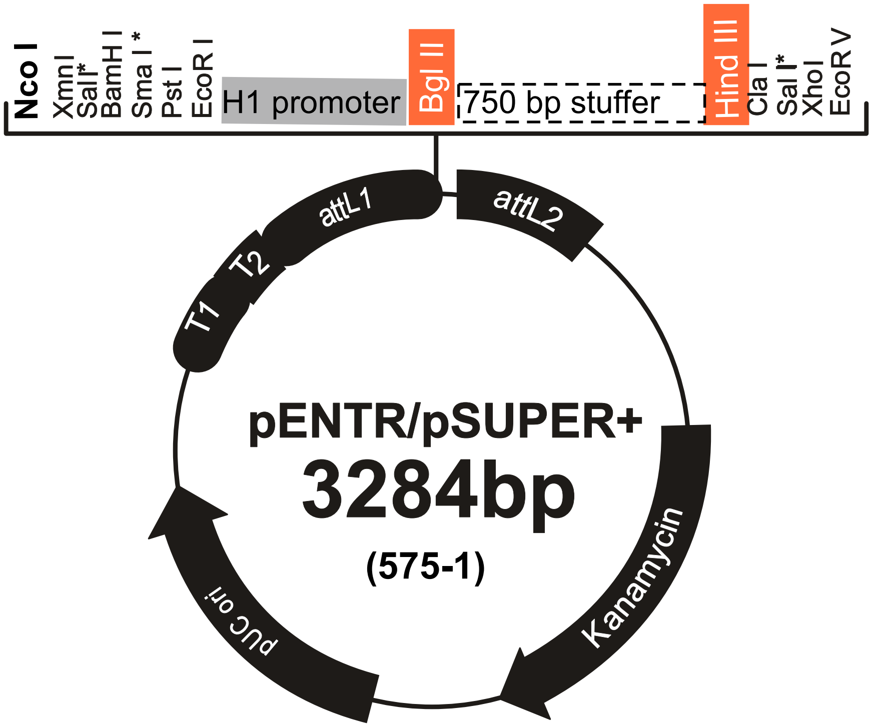

Comments for pENTR/pSUPER+
3313 nucleotides

*rrn*B T1 transcription termination sequence: bases 106-149
*rrn*B T2 transcription termination sequence: bases 281-308
*att*L1: bases 358-457 (complementary strand)
H1 RNA promoter: bases 494-715
750 bp stuffer: bases 716-1472
*att*L2: bases 1542-1640
Kanamycin resistance gene: bases 1764-2573
pUC origin: bases 2635-3284

*Sites are not unique

Recommended *E.coli* strain for propagation: TOP10F'

Eric Campeau July 2004
