# Supplementary material for: A Versatile Viral System for Expression and Depletion of Proteins in Mammalian Cells
Source: PLoS One. 2009 Aug 6;4(8):e6529. doi: 10.1371/journal.pone.0006529 (PMC2717805; doi:10.1371/journal.pone.0006529)
Supplement: File S1 — Vector maps and sequences (5.49 MB ZIP) [file pone.0006529.s005.zip › pENTR_pTER+ (430-1).pdf]

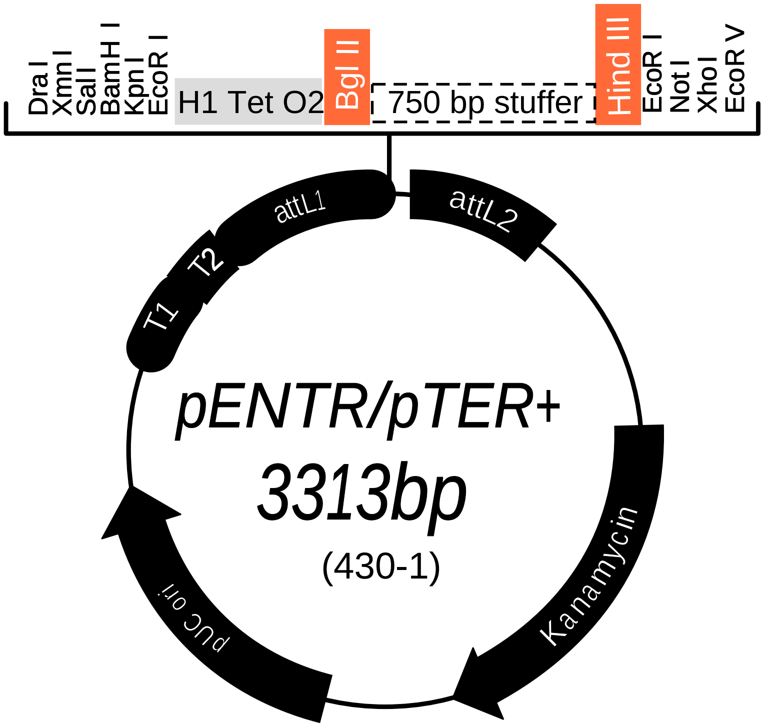

Comments for pENTR/pTER+
3313 nucleotides

rrnB T1 transcription termination sequence: bases 106-149
rrnB T2 transcription termination sequence: bases 281-308
attL1: bases 358-457 (complementary strand)
H1 RNA promoter: bases 494-715
750 bp stuffer: bases 716-1472
attL2: bases 1542-1640
Kanamycin resistance gene: bases 1764-2573
pUC origin: bases 2635-3308

Eric Campeau March 2004
