# Supplementary material for: A Versatile Viral System for Expression and Depletion of Proteins in Mammalian Cells
Source: PLoS One. 2009 Aug 6;4(8):e6529. doi: 10.1371/journal.pone.0006529 (PMC2717805; doi:10.1371/journal.pone.0006529)
Supplement: File S1 — Vector maps and sequences (5.49 MB ZIP) [file pone.0006529.s005.zip › pLenti CMV GFP DEST (736-1).pdf]

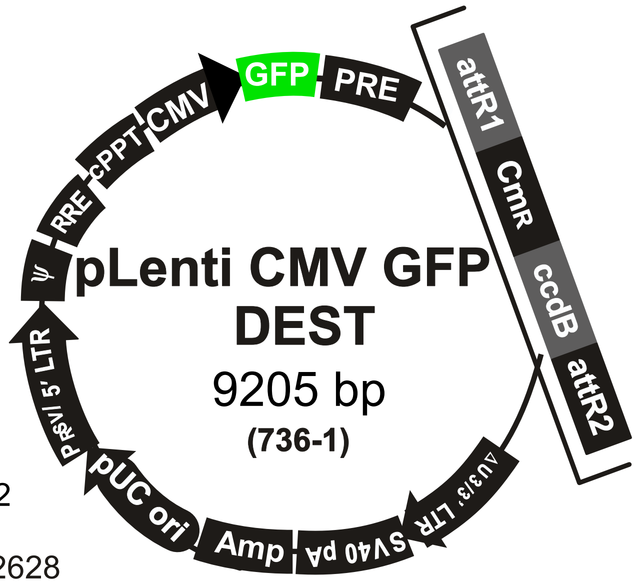

Comments for pLenti GFP DEST
9205 nucleotides

bla promoter: bases 32-131
ampicillin resistance gene: bases 132-992
pUC origin: bases 1138-1810
RSV/5LTR hybrid promoter: bases 2218-2628
HIV-1 psi (ψ)packaging signal: bases 2736-2781
HIV-1 Rev response element (RRE): bases 3272-3525
3 splice acceptor: base 3862
3 splice acceptor: base 3901
Central polypurine tract (cPPT): bases 4011-4073
CMV promoter: bases 4145-4718
Green Fluorescent Protein (GFP): bases 4755-5474
Woodchuck post-transcriptional element (PRE): bases 5493-6082
*att*R1 site: bases 6134-6259
Chloramphenicol resistance gene (Cm): bases 6368-7027
*ccd*B gene: bases 7368-7674
*att*R2 site: bases 7713-7840
ΔU3/3‘LTR: bases 7945-8180
SV40 polyadenylation signal: bases 8250-8403

NOTE: This is not an Invitrogen plasmid. The position of the genetic elements is approximative.

Eric Campeau March 2005
