# Supplementary material for: A Versatile Viral System for Expression and Depletion of Proteins in Mammalian Cells
Source: PLoS One. 2009 Aug 6;4(8):e6529. doi: 10.1371/journal.pone.0006529 (PMC2717805; doi:10.1371/journal.pone.0006529)
Supplement: File S1 — Vector maps and sequences (5.49 MB ZIP) [file pone.0006529.s005.zip › pLenti CMV TetR Blast (716-1).pdf]

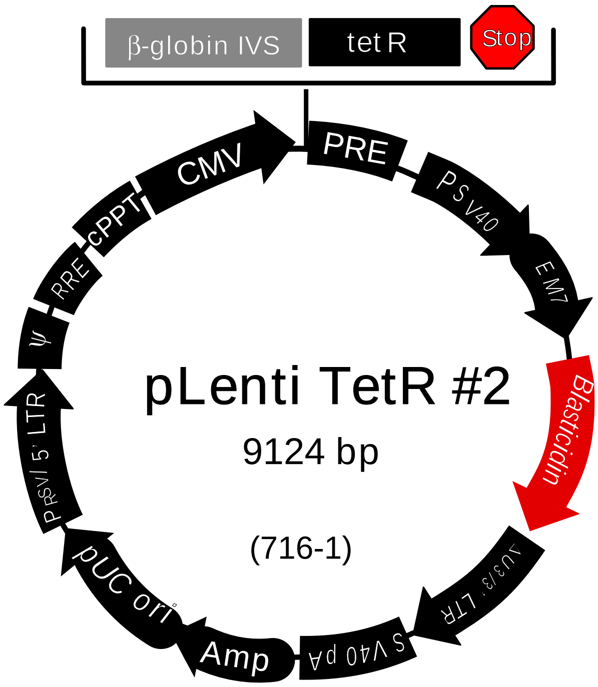

Comments for pLenti TetR #2
9124 nucleotides

bla promoter: bases 32-131
ampicillin resistance gene: bases 132-982
pUC origin: bases 1138-1810
RSV/5'LTR hybrid promoter: bases 2218-2628
HIV-1 psi (ψ)packaging signal: bases 2736-2781
HIV-1 Rev response element (RRE): bases 3272-3525
3' splice acceptor: base 3862
3' splice acceptor: base 3901
Central polypurine tract (cPPT): bases 4011-4073
CMV promoter: bases 4145-4730
Rabbit β-globin intron II (IVS): bases
tet R gene: bases
Woodchuck post-transcriptional element (PRE): bases
SV40 early promoter and origin: bases
EM7 promoter: bases
Blasticidin resistance gene: bases
ΔU3/3'LTR: bases
SV40 polyadenylation signal: bases

Recommended E.coli strain for propagation: Stbl3

NOTE: This is not an Invitrogen plasmid. The position of the genetic elements is approximative.

Eric Campeau February 2005
