# Supplementary material for: A Versatile Viral System for Expression and Depletion of Proteins in Mammalian Cells
Source: PLoS One. 2009 Aug 6;4(8):e6529. doi: 10.1371/journal.pone.0006529 (PMC2717805; doi:10.1371/journal.pone.0006529)
Supplement: File S1 — Vector maps and sequences (5.49 MB ZIP) [file pone.0006529.s005.zip › pLenti CMV_TO GFP-Zeo DEST (719-1).pdf]

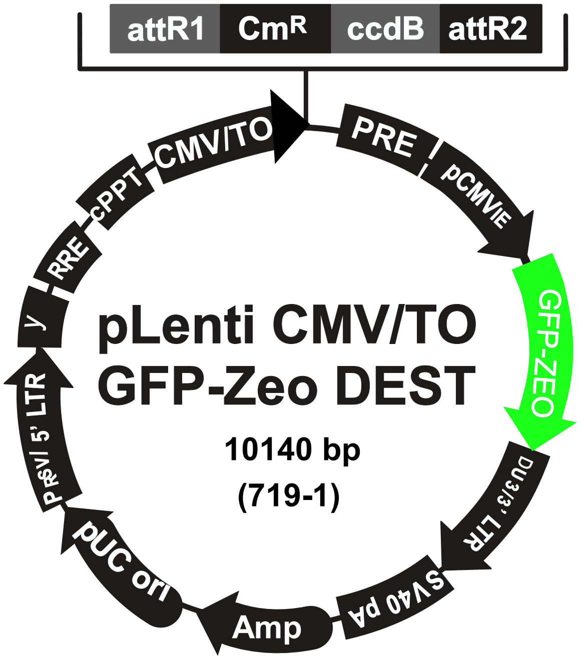

Comments for pLenti CMV/TO GFP-Zeo DEST
10140 nucleotides

bla promoter: bases 32-131
ampicillin resistance gene: bases 132-982
pUC origin: bases 1138-1808
RSV/5LTR hybrid promoter: bases 2212-2632
5' splice donor: base
HIV-1 psi (y)packaging signal: bases 2736-2782
HIV-1 Rev response element (RRE): bases 3272-3526
Central polypurine tract (cPPT): bases 4010-4074
CMV/TO promoter: bases
*att*R1 site: bases
Chloramphenicol resistance gene (Cm): bases
*ccd*B gene: bases
*att*R2 site: bases
Woodchuck post-transcriptional element (PRE): bases
Cytomegalovirus immediate early promoter (CMVie): bases
GFP-Zeocin resistance gene: bases
ΔU3/3'LTR: bases
SV40 polyadenylation signal: bases

Recommended E.coli strain for propagation: DB3.1, ccDB survival under ampicillin and chloramphinecol selection.

Eric Campeau February 2005
