# Supplementary material for: A Versatile Viral System for Expression and Depletion of Proteins in Mammalian Cells
Source: PLoS One. 2009 Aug 6;4(8):e6529. doi: 10.1371/journal.pone.0006529 (PMC2717805; doi:10.1371/journal.pone.0006529)
Supplement: File S1 — Vector maps and sequences (5.49 MB ZIP) [file pone.0006529.s005.zip › pLenti CMV_TO Neo DEST (685-3).pdf]

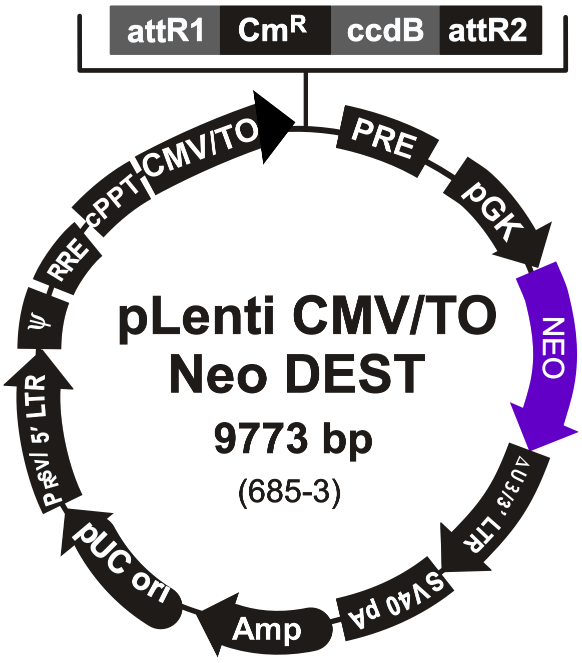

Comments for pLenti CMV/TO Neo DEST
9773 nucleotides

bla promoter: bases 32-131
ampicillin resistance gene: bases 132-992
pUC origin: bases 1138-1810
RSV/5LTR hybrid promoter: bases 2218-2628
HIV-1 psi (ψ)packaging signal: bases 2736-2781
HIV-1 Rev response element (RRE): bases 3272-3525
3' splice acceptor: base 3862
3' splice acceptor: base 3901
Central polypurine tract (cPPT): bases 4011-4073
CMV/TO promoter: bases 4145-4702
*att*R1 site: bases 4740-4864
Chloramphenicol resistance gene (Cm): bases 4974-5633
*ccd*B gene: bases 5975-6280
*att*R2 site: bases 6320-6446
Woodchuck post-transcriptional element (PRE): bases 6475-7067
Murine phosphoglycerate kinase (pGK) promoter: bases 7068-7607
Neomycin resistance gene: bases 7620-8423
ΔU3/3'LTR: bases 8515-8747
SV40 polyadenylation signal: bases 8820-8973

For propagation in *E.coli*, use either DB3.1 or ccdB survival strain under ampicillin and chloramphinecol selection.

Eric Campeau January 2005
