# Supplementary material for: A Versatile Viral System for Expression and Depletion of Proteins in Mammalian Cells
Source: PLoS One. 2009 Aug 6;4(8):e6529. doi: 10.1371/journal.pone.0006529 (PMC2717805; doi:10.1371/journal.pone.0006529)
Supplement: File S1 — Vector maps and sequences (5.49 MB ZIP) [file pone.0006529.s005.zip › pLenti CMV_TO Zeo DEST (644-1).pdf]

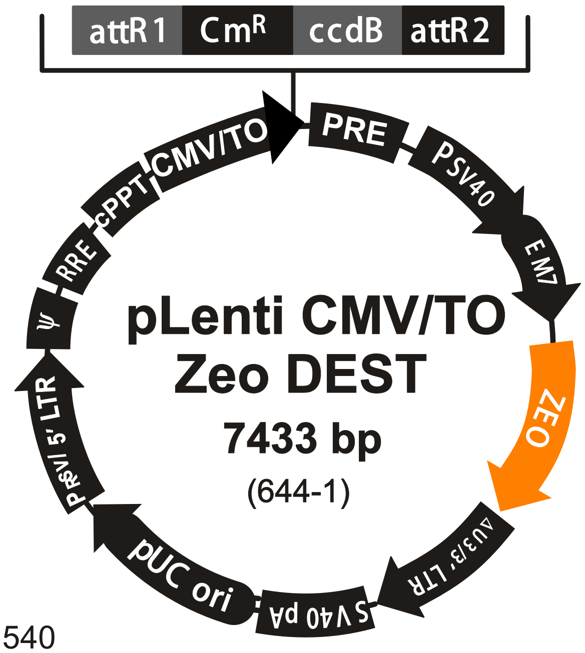

Comments for pLenti CMV/TO Zeo DEST
7433 nucleotides

pUC origin: bases 28-716
RSV/5LTR hybrid promoter: bases 1121-1540
5' splice donor: base
HIV-1 psi (ψ)packaging signal: bases 1643-1691
HIV-1 Rev response element (RRE): bases 2180-2438
3' splice acceptor: base 2772
3' splice acceptor: base 2800
Central polypurine tract (cPPT): bases 2916-2980
CMV/TO promoter: bases 3054-3608
*att*R1 site: bases 3648-3774
Chloramphenicol resistance gene (Cm): bases 3883-4542
*ccd*B gene: bases 4883-5189
*att*R2 site: bases 5229-5360
Woodchuck post-transcriptional element (PRE): bases 5384-5977
SV40 early promoter and origin: bases 6037-6353
EM7 promoter: bases 6369-6435
Zeocin resistance gene: bases 6436-6811
ΔU3/3‘LTR: bases 6902-7143
SV40 polyadenylation signal: bases 7207-7371

Recommended *E.coli* strain for propagation: DB3.1 oe ccDB survival' under zeocin and chloramphinecol selection.

Eric Campeau January 2005
