# Supplementary material for: A Versatile Viral System for Expression and Depletion of Proteins in Mammalian Cells
Source: PLoS One. 2009 Aug 6;4(8):e6529. doi: 10.1371/journal.pone.0006529 (PMC2717805; doi:10.1371/journal.pone.0006529)
Supplement: File S1 — Vector maps and sequences (5.49 MB ZIP) [file pone.0006529.s005.zip › pLenti PGK Puro DEST (w529-2).pdf]

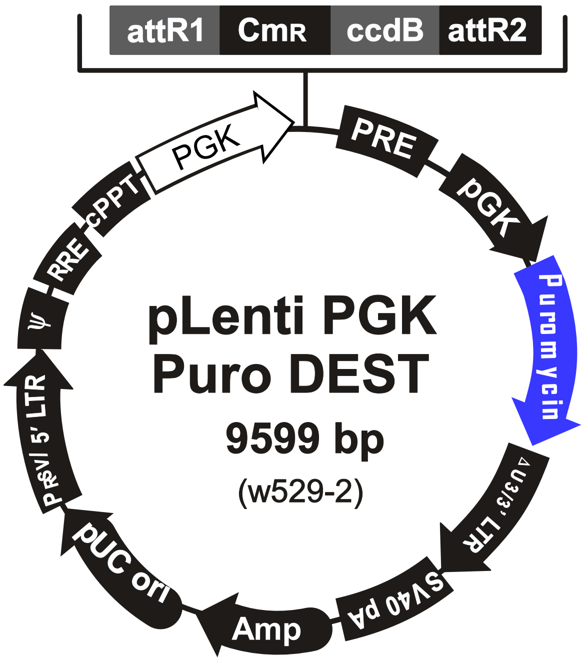

Comments for pLenti PGK Puro DEST
9599 nucleotides

bla promoter: bases 32-131
ampicillin resistance gene: bases 132-992
pUC origin: bases 1138-1810
RSV/5LTR hybrid promoter: bases 2218-2628
HIV-1 psi (ψ)packaging signal: bases 2736-2781
HIV-1 Rev response element (RRE): bases 3272-3525
3' splice acceptor: base 3862
3' splice acceptor: base 3901
Central polypurine tract (cPPT): bases 4011-4073
Human phosphoglycerate kinase (PGK) promoter: bases 4179-4694
*att*R1 site: bases 4745-4870
Chloramphenicol resistance gene (Cm): bases 4979-5638
*ccd*B gene: bases 5980-6285
*att*R2 site: bases 6325-6451
Woodchuck post-transcriptional element (PRE): bases 6480-7072
Murine phosphoglycerate kinase (pGK) promoter: bases 7079-7600
Puromycin resistance gene: bases 7603-8202
ΔU3/3'LTR: bases 8339-8577
SV40 polyadenylation signal: bases 8645-8779

Recommended *E.coli* strain for propagation: DB3.1 or ccDB survival'

NOTE: This is not an Invitrogen plasmid. The position of the genetic elements is approximative.

Eric Campeau July 2008
