# Supplementary material for: A Versatile Viral System for Expression and Depletion of Proteins in Mammalian Cells
Source: PLoS One. 2009 Aug 6;4(8):e6529. doi: 10.1371/journal.pone.0006529 (PMC2717805; doi:10.1371/journal.pone.0006529)
Supplement: File S1 — Vector maps and sequences (5.49 MB ZIP) [file pone.0006529.s005.zip › pLenti X1 GFP-Zeo DEST (720-2).pdf]

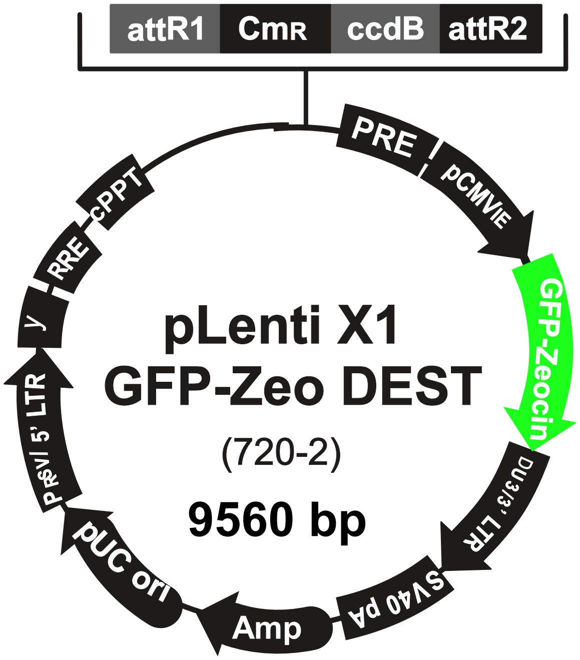

Comments for pLenti x1 GFP-Zeo DEST
9560 nucleotides

bla promoter: bases 32-131
ampicillin resistance gene: bases 132-982
pUC origin: bases 1138-1808
RSV/5LTR hybrid promoter: bases 2212-2632
5' splice donor: base
HIV-1 psi (y)packaging signal: bases 2736-2782
HIV-1 Rev response element (RRE): bases 3272-3526
Central polypurine tract (cPPT): bases 4010-4074
*att*R1 site: bases 4159-4284
Chloramphenicol resistance gene (Cm): bases 4394-5053
*ccd*B gene: bases 5395-5700
*att*R2 site: bases 5740-5866
Woodchuck post-transcriptional element (PRE): bases 5896-6488
Cytomegalovirus immediate early promoter (CMVie): bases 6491-7090
GFP-Zeocin resistance gene: bases 7111-8208
∆U3/3‘LTR: bases 8298-8543
SV40 polyadenylation signal: bases 8604-8779

Use DB3.1 or ccDB survival for propagation under ampicillin and chloramphinecol selection.

NOTE: The position of the genetic elements is approximative.

Eric Campeau February 2005
