# Supplementary material for: A Versatile Viral System for Expression and Depletion of Proteins in Mammalian Cells
Source: PLoS One. 2009 Aug 6;4(8):e6529. doi: 10.1371/journal.pone.0006529 (PMC2717805; doi:10.1371/journal.pone.0006529)
Supplement: File S1 — Vector maps and sequences (5.49 MB ZIP) [file pone.0006529.s005.zip › pLenti X1 Puro DEST (694-6).pdf]

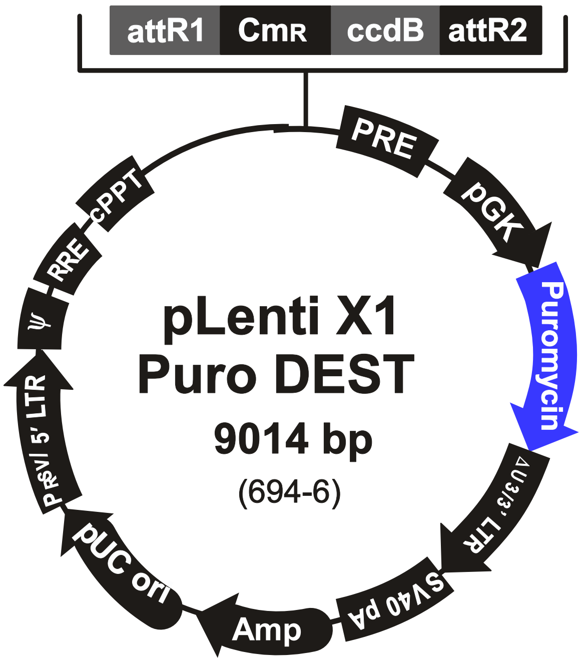

Comments for pLenti X1 Puro DEST
9014 nucleotides

bla promoter: bases 32-131
ampicillin resistance gene: bases 132-992
pUC origin: bases 1138-1810
RSV/5LTR hybrid promoter: bases 2218-2628
HIV-1 psi (ψ)packaging signal: bases 2736-2781
HIV-1 Rev response element (RRE): bases 3272-3525
3' splice acceptor: base 3862
3' splice acceptor: base 3901
Central polypurine tract (cPPT): bases 4011-4073
*att*R1 site: bases 4159-4285
Chloramphenicol resistance gene (Cm): bases 4394-5053
*ccd*B gene: bases 5395-5700
*att*R2 site: bases 5741-5866
Woodchuck post-transcriptional element (PRE): bases 5896-6487
Murine phosphoglycerate kinase (pGK) promoter: bases 6490-6998
Puromycin resistance gene: bases 7018-7617
ΔU3/3'LTR: bases 7756-7988
SV40 polyadenylation signal: bases 8060-8212

Suggested E.coli strain: DB3.1 or ccDB survival

NOTE: The position of the genetic elements is approximative.

Eric Campeau February 2005
