# Supplementary material for: A Versatile Viral System for Expression and Depletion of Proteins in Mammalian Cells
Source: PLoS One. 2009 Aug 6;4(8):e6529. doi: 10.1371/journal.pone.0006529 (PMC2717805; doi:10.1371/journal.pone.0006529)
Supplement: File S1 — Vector maps and sequences (5.49 MB ZIP) [file pone.0006529.s005.zip › pLenti X1 Zeo DEST (668-1).pdf]

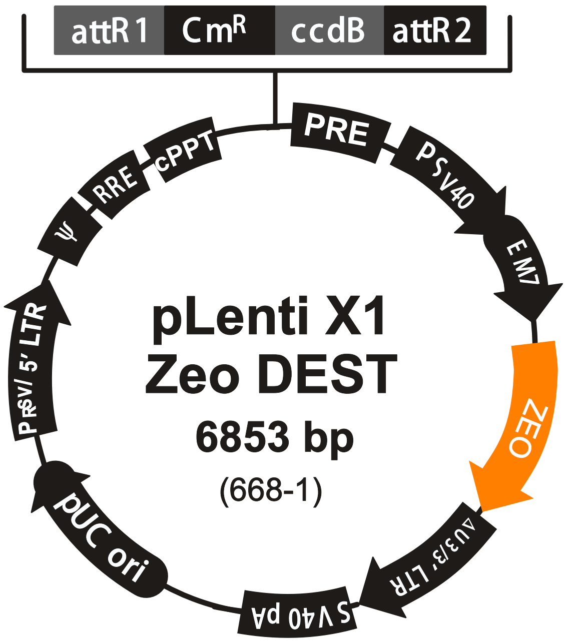

Comments for pLenti X1 Zeo DEST
6853 nucleotides

pUC origin: bases 28-716
RSV/5LTR hybrid promoter: bases 1121-1540
5' splice donor: base
HIV-1 psi (ψ)packaging signal: bases 1643-1691
HIV-1 Rev response element (RRE): bases 2180-2438
3' splice acceptor: base 2772
3' splice acceptor: base 2800
Central polypurine tract (cPPT): bases 2916-2980
*att*R1 site: bases 3068-3195
Chloramphenicol resistance gene (Cm): bases 3230-3962
*ccd*B gene: bases 4304-4609
*att*R2 site: bases 4649-4781
Woodchuck post-transcriptional element (PRE): bases 4804-5397
SV40 early promoter and origin: bases 5456-5780
EM7 promoter: bases 5788-5856
Zeocin resistance gene: bases 5857-6231
ΔU3/3'LTR: bases 6322-6564
SV40 polyadenylation signal: bases 6628-6792

NOTE: Use Zeocin and chloramphinecol to propagate the plasmid in *E.coli*. Use strain DB3.1 or ccdB survival.

Location of the genetic elements is approximative.

Eric Campeau February 2005
