# Supplementary material for: A Versatile Viral System for Expression and Depletion of Proteins in Mammalian Cells
Source: PLoS One. 2009 Aug 6;4(8):e6529. doi: 10.1371/journal.pone.0006529 (PMC2717805; doi:10.1371/journal.pone.0006529)
Supplement: File S1 — Vector maps and sequences (5.49 MB ZIP) [file pone.0006529.s005.zip › pLenti X2 Blast DEST (w46-2).pdf]

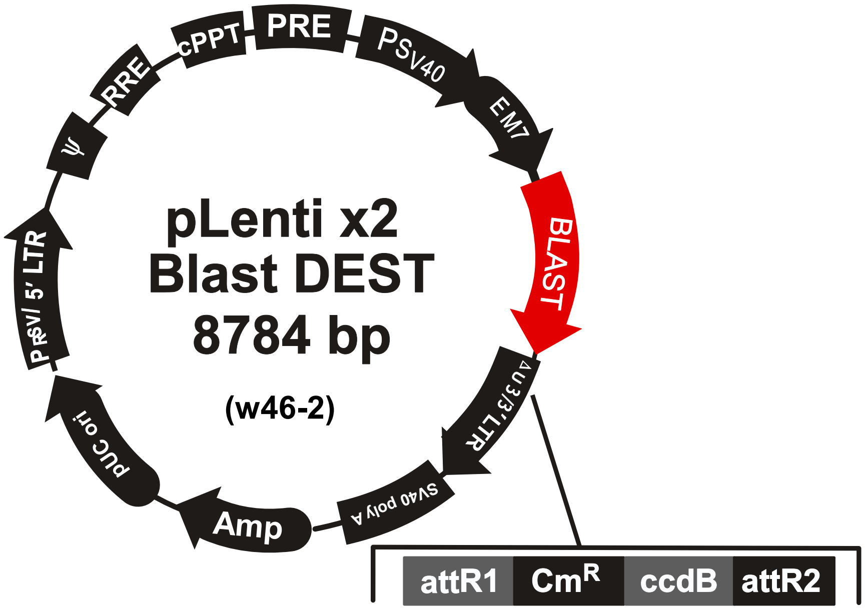

Comments for pLenti X2 Blast DEST
8784 nucleotides

bla promoter: bases 32-131
ampicillin resistance gene: bases 132-992
pUC origin: bases 1138-1810
RSV/5LTR hybrid promoter: bases 2218-2628
HIV-1 psi (ψ)packaging signal: bases 2736-2781
HIV-1 Rev response element (RRE): bases 3272-3525
3' splice acceptor: base 3862
3' splice acceptor: base 3901
Central polypurine tract (cPPT): bases 4011-4073
Woodchuck post-transcriptional element (PRE): bases 4182-4774
SV40 promoter: bases 4823-5164
EM7 promoter: bases 5205-5270
Blasticidin resistance gene: bases 5271-5669
ΔU3/3'LTR: bases 5690-7757
*att*R2 site: bases 5923-5799
*ccd*B gene: bases 6269-5964
Chloramphenicol resistance gene (Cm): bases 7270-6611
*att*R1 site: bases 7504-7380
SV40 polyadenylation signal: bases 7829-7983

Recommended *E.coli* strain for propagation: DB3.1 or ccDB survival.

Eric Campeau October 2005
