# Supplementary material for: A Versatile Viral System for Expression and Depletion of Proteins in Mammalian Cells
Source: PLoS One. 2009 Aug 6;4(8):e6529. doi: 10.1371/journal.pone.0006529 (PMC2717805; doi:10.1371/journal.pone.0006529)
Supplement: File S1 — Vector maps and sequences (5.49 MB ZIP) [file pone.0006529.s005.zip › pLenti X2 Hygro DEST (w17-1).pdf]

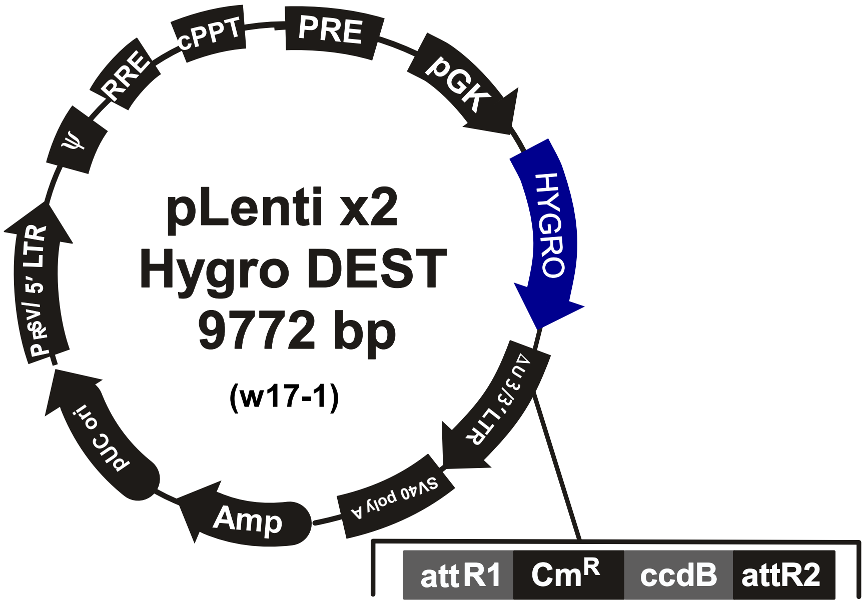


**Comments for pLenti X2 Hygro DEST**
**9772 nucleotides**

bla promoter: bases 32-131
ampicillin resistance gene: bases 132-992
pUC origin: bases 1138-1810
RSV/5LTR hybrid promoter: bases 2218-2628
HIV-1 psi (ψ)packaging signal: bases 2736-2781
HIV-1 Rev response element (RRE): bases 3272-3525
3′ splice acceptor: base 3862
3′ splice acceptor: base 3901
Central polypurine tract (cPPT): bases 4011-4073
Woodchuck post-transcriptional element (PRE): bases 4182-4774
Murine phosphoglycerate kinase (pGK) promoter: bases 4780-5284
Hygromycin resistance gene: bases 5344-6370
ΔU3/3‘LTR: bases 6680-8743
*att*R2 site: bases 6912-6787
*ccd*B gene: bases 7257-6952
Chloramphenicol resistance gene (Cm): bases 8258-7599
*att*R1 site: bases 8492-8368
SV40 polyadenylation signal: bases 8817-8971

Recommended *E.coli* strain for propagation: DB3.1 or ccDB survival.

**NOTE: This is not an Invitrogen plasmid. The position of the genetic elements is approximative.**

Eric Campeau September 2005
