# Supplementary material for: A Versatile Viral System for Expression and Depletion of Proteins in Mammalian Cells
Source: PLoS One. 2009 Aug 6;4(8):e6529. doi: 10.1371/journal.pone.0006529 (PMC2717805; doi:10.1371/journal.pone.0006529)
Supplement: File S1 — Vector maps and sequences (5.49 MB ZIP) [file pone.0006529.s005.zip › pLenti X2 Neo DEST (w18-1).pdf]

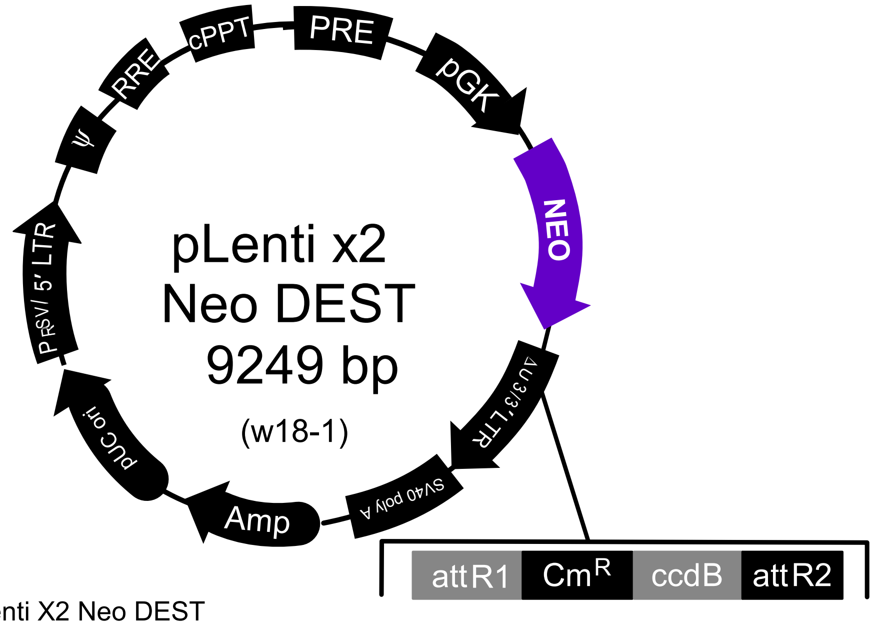

Comments for pLenti X2 Neo DEST
9249 nucleotides

bla promoter: bases 32-131
ampicillin resistance gene: bases 132-992
pUC origin: bases 1138-1810
RSV/5LTR hybrid promoter: bases 2218-2628
HIV-1 psi (ψ)packaging signal: bases 2736-2781
HIV-1 Rev response element (RRE): bases 3272-3525
3' splice acceptor: base 3862
3' splice acceptor: base 3901
Central polypurine tract (cPPT): bases 4011-4073
Woodchuck post-transcriptional element (PRE): bases 4182-4774
Murine phosphoglycerate kinase (pGK) promoter: bases 4805-5313
Neomycin resistance gene: bases 5327-6130
ΔU3/3'LTR: bases 6155-8222
attR2 site: bases 6388-6264
ccdB gene: bases 6734-6429
Chloramphenicol resistance gene (Cm): bases 7735-7076
attR1 site: bases 7969-7845
SV40 polyadenylation signal: bases 8294-8449

Recommended E.coli strain for propagation: DB3.1 or ccDB survival.

NOTE: This is not an Invitrogen plasmid. The position of the genetic elements is approximative.

Eric Campeau September 2005
