# Supplementary material for: A Versatile Viral System for Expression and Depletion of Proteins in Mammalian Cells
Source: PLoS One. 2009 Aug 6;4(8):e6529. doi: 10.1371/journal.pone.0006529 (PMC2717805; doi:10.1371/journal.pone.0006529)
Supplement: File S1 — Vector maps and sequences (5.49 MB ZIP) [file pone.0006529.s005.zip › pLenti X2 Zeo DEST (749-3).pdf]

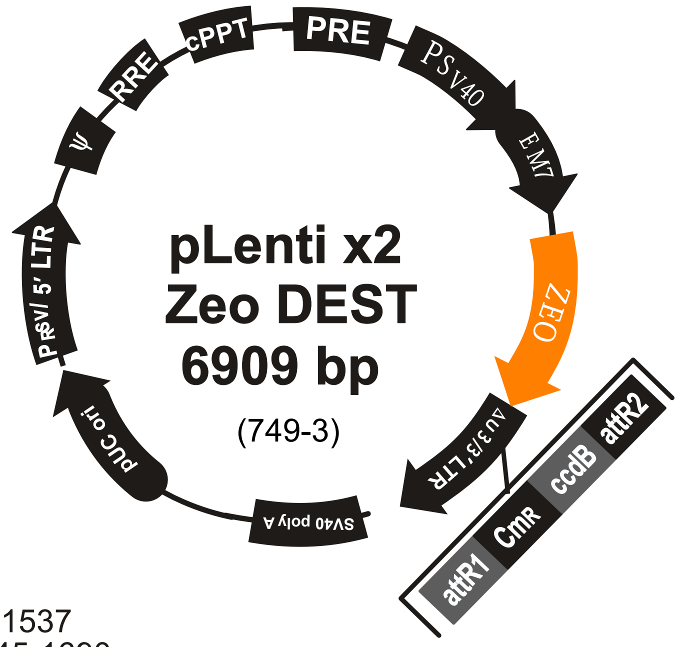

Comments for pLenti X2 Zeo DEST
6909 nucleotides

pUC origin: bases 48-714
RSV/5LTR hybrid promoter: bases 1127-1537
HIV-1 psi (ψ)packaging signal: bases 1645-1690
HIV-1 Rev response element (RRE): bases 2181-2436
3' splice acceptor: base 2779
3' splice acceptor: base 2807
Central polypurine tract (cPPT): bases 2920-2982
Woodchuck post-transcriptional element (PRE): bases 3091-3684
SV40 early promoter and origin: bases 3748-4074
EM7 promoter: bases 4078-4143
Zeocin resistance gene: bases 4144-4518
ΔU3/3'LTR: bases 4612-6613
*att*R2 site: bases 4779-4654
*ccd*B gene: bases 5124-4819
Chloramphenicol resistance gene (Cm): bases 6125-5466
*att*R1 site: bases 6360-6235
SV40 polyadenylation signal: bases 6684-6837

NOTE: This vector does not carry the ampicillin resistance gene and is selected with Zeocin (50 ug/ml in low salt LB) in *E.coli.*

Recommended *E.coli* strain for propagation: DB3.1 or ccDB survival.
The position of the genetic elements is approximative.

Eric Campeau May 2005
