# Supplementary material for: A Versatile Viral System for Expression and Depletion of Proteins in Mammalian Cells
Source: PLoS One. 2009 Aug 6;4(8):e6529. doi: 10.1371/journal.pone.0006529 (PMC2717805; doi:10.1371/journal.pone.0006529)
Supplement: File S1 — Vector maps and sequences (5.49 MB ZIP) [file pone.0006529.s005.zip › pQCXIN CMV_TO DEST (w383-1).pdf]

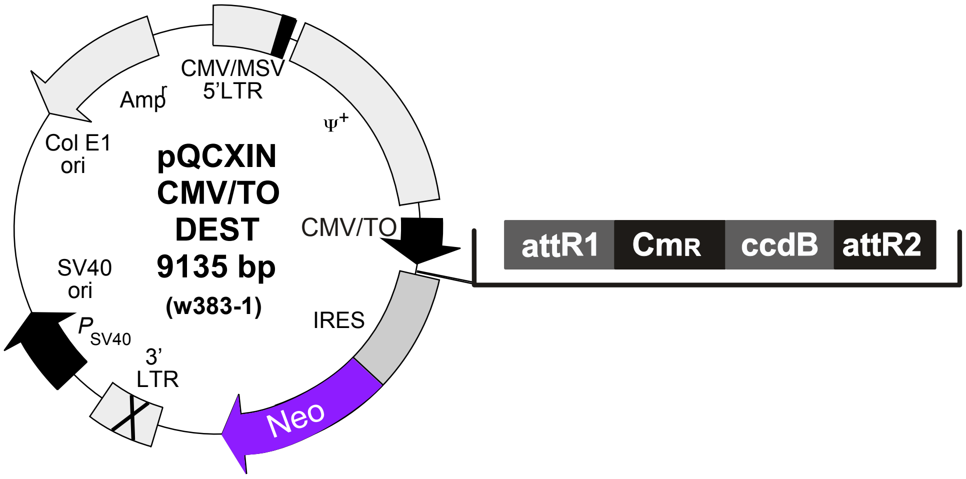

Comments on pQCXIN CMV/TO DEST, 9135 nucleotides:

5' LTR (CMV/MSV): 1–728
Cytomegalovirus (CMV)/ mouse sarcoma virus (MSV) hybrid promoter: 1–511
R region: 584–654
U5 region: 655–728
ψ+ (extended packaging signal): 758–1567
CMV/TO promoter: 1601-2170
attR1:2198-2322
Chloramphinecol resistance gene:2431-3090
ccdB gene:3432-3737
attR2:3778-3900
Internal ribosome entry site (IRES): 4043-4617
Neomycin resistance gene (Neo): 4630-5424
3' MoMuLV LTR (deletion in U3): 5841-6265
SV40 promoter: 6546-6812
SV40 ori: 6767-6832
Col E1 ori (Site of replication initiation): 7153
Ampicillin resistance gene (reverse orientation):8773-7913

Use *E.coli* DB3.1 or ccDB survival strain for propagation.

NOTE: Use of this plasmid in a T-Rex cell line will prevent expression of the Blasticidin selection gene.

Eric Campeau April 2007
