# Supplementary material for: A Versatile Viral System for Expression and Depletion of Proteins in Mammalian Cells
Source: PLoS One. 2009 Aug 6;4(8):e6529. doi: 10.1371/journal.pone.0006529 (PMC2717805; doi:10.1371/journal.pone.0006529)
Supplement: File S1 — Vector maps and sequences (5.49 MB ZIP) [file pone.0006529.s005.zip › pQCXIN X2 DEST (w310-1).pdf]

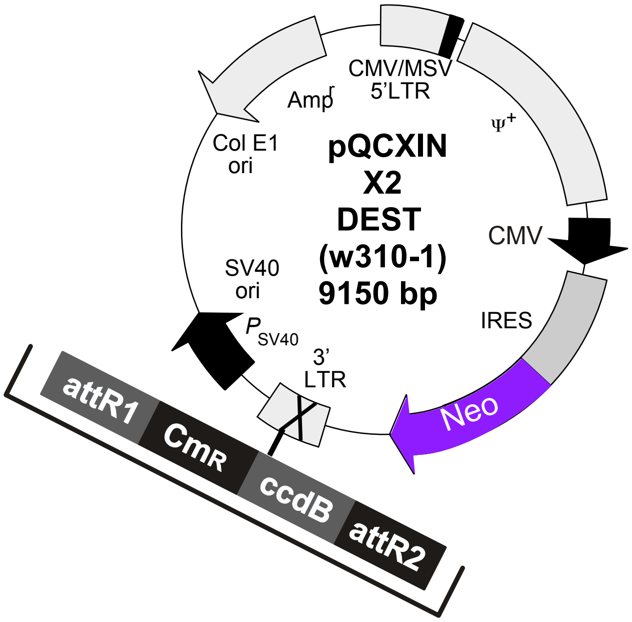

Comments on pQCXIN CMV/TO DEST, 9150 nucleotides:

5‘ LTR (CMV/MSV): 1–728
Cytomegalovirus (CMV)/ mouse sarcoma virus (MSV) hybrid promoter: 1–511
R region: 584–654
U5 region: 655–728
y+ (extended packaging signal): 758–1567
CMV promoter: 1601-2170
Internal ribosome entry site (IRES): 2289-2862
Neomycin resistance gene (NEO): 2876-3670
3' MoMuLV LTR (deletion in U3): 4077-6281
attR2:4277-4152
ccdB gene:4622-4318
Chloramphinecol resistance gene:5624-4965
attR1:5858-5734
SV40 promoter: 6560-6827
SV40 ori: 6782-6846
Col E1 ori (Site of replication initiation): 7168
Ampicillin resistance gene (reverse orientation):8788-7928

Use *E.coli* DB3.1 or ccDB survival strain for propagation.

NOTE: Use of this plasmid in a T-Rex cell line will prevent expression of the Neomycin selection gene.

Eric Campeau April 2007
