# Supplementary material for: A Versatile Viral System for Expression and Depletion of Proteins in Mammalian Cells
Source: PLoS One. 2009 Aug 6;4(8):e6529. doi: 10.1371/journal.pone.0006529 (PMC2717805; doi:10.1371/journal.pone.0006529)
Supplement: File S1 — Vector maps and sequences (5.49 MB ZIP) [file pone.0006529.s005.zip › pQCXIP X2 DEST (w270-1).pdf]

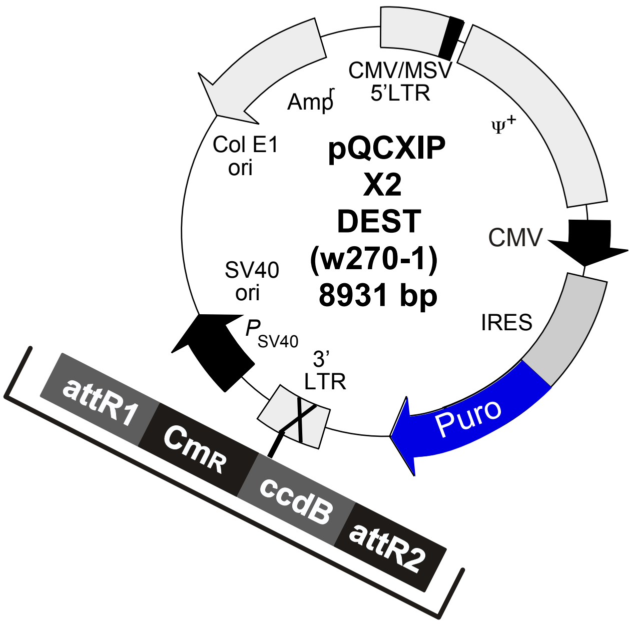

Comments on pQCXIP X2 DEST, 8931 nucleotides:

5‘ LTR (CMV/MSV): 1–728
Cytomegalovirus (CMV)/ mouse sarcoma virus (MSV) hybrid promoter: 1–511
R region: 584–654
U5 region: 655–728
y+ (extended packaging signal): 758–1567
CMV promoter: 1601-2170
Internal ribosome entry site (IRES): 2289-2862
Puromycin resistance gene (Puro): 2895-3494
3' MoMuLV LTR (deletion in U3): 3868-6062
attR2: 4060-3934
ccdB gene: 4404-4099
Chloramphinecol resistance gene:5405-4746
attR1: 5638-5515
SV40 promoter: 6341-6608
SV40 ori: 6563-6629
Col E1 ori (Site of replication initiation): 6950
Ampicillin resistance gene (reverse orientation):8569-7707

Use *E.coli* DB3.1 or ccDB survival strain for propagation.

NOTE: Use of this plasmid in a T-Rex cell line will prevent expression of the Neomycin selection gene.

Eric Campeau April 2007
