# Supplementary material for: A Versatile Viral System for Expression and Depletion of Proteins in Mammalian Cells
Source: PLoS One. 2009 Aug 6;4(8):e6529. doi: 10.1371/journal.pone.0006529 (PMC2717805; doi:10.1371/journal.pone.0006529)
Supplement: File S1 — Vector maps and sequences (5.49 MB ZIP) [file pone.0006529.s005.zip › pQCXP CMV_TO DEST (w360-1).pdf]

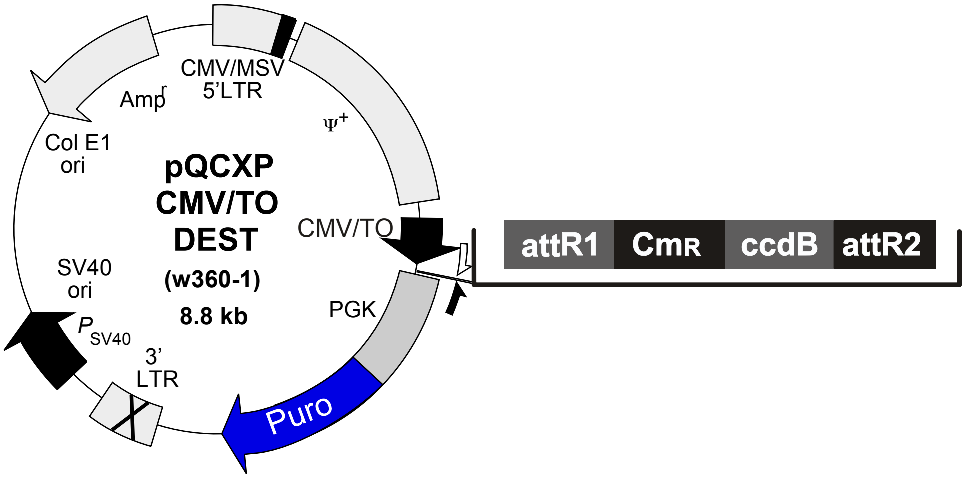

Comments on pQCXP CMV/TO DEST, 8832 nucleotides:

5' LTR (CMV/MSV): 1–728
Cytomegalovirus (CMV)/ mouse sarcoma virus (MSV) hybrid promoter: 1–511
R region: 584–654
U5 region: 655–728
ψ+ (extended packaging signal): 758–1567
CMV/TO promoter: 1601-2159
attR1:2198-2322
Chloramphinecol resistance gene:2431-3090
ccdB gene:3432-3737
attR2:3778-3900
Murine phosphoglycerate kinase (PKG) promoter:4041-4542
Puromycin resistance gene (Puro): 4564-5164
3' MoMuLV LTR (deletion in U3): 5538-5963
SV40 promoter: 6242-6509
SV40 ori: 6464-6529
Col E1 ori (Site of replication initiation): 6851
Ampicillin resistance gene (reverse orientation):8470-7609

Use *E.coli* DB3.1 or ccDB survival strain for propagation.

Eric Campeau April 2007
